# Peptidomimetic Phenoxymethyl Ketone Warheads as Potent Dual-Mode Inhibitors against SARS-CoV‑2 M^pro^ and Cathepsin

**DOI:** 10.1021/acs.jmedchem.4c03147

**Published:** 2025-05-26

**Authors:** Miriam R.B. Porzberg, G.J. Mirjam Groenewold, Heyrhyoung Lyoo, Alexander K.M.H. Jakob, Willem H.C. Titulaer, Lorenzo Cavina, Katrien C.K. Poelaert, Marleen Zwaagstra, Cindy E.J. Dieteren, Jaap G.H. Lemmers, S. Hakim Hamdani, Bernd N.M. van Buuren, Bart Ackerschott, Johannes Platteeuw, Joey Michorius, Byron E.E. Martina, Martin C. Feiters, Daniel Gironés, Frank J.M. van Kuppeveld, Martijn J. van Hemert, Floris P.J.T. Rutjes

**Affiliations:** 1 Institute for Molecules and Materials, 6029 Radboud University, Heyendaalseweg 135, Nijmegen 6525 AJ, The Netherlands; 2 Molecular Virology Laboratory, Leiden University Center for Infectious Diseases, 4501 Leiden University Medical Center, Albinusdreef 2, Leiden 2333ZA, The Netherlands; 3 Virology Section, Division of Infectious Diseases & Immunology, Department of Biomolecular Health Sciences, Faculty of Veterinary Medicine, 8125 Utrecht University, Yalelaan 1, Utrecht 3584 CL, The Netherlands; 4 Protinhi Therapeutics, Transistorweg 5, Nijmegen 6534 AT, The Netherlands; 5 Avivia BV, Transistorweg 5, Nijmegen 6534 AT, The Netherlands; 6 Artemis Bioservices, Molengraaffsingel 10, Delft 2629 JD, The Netherlands

## Abstract

Five years after the onset of the COVID-19 pandemic, there still is an unmet need for novel antivirals to battle SARS-CoV-2 and other coronaviruses. For this purpose, the development of peptidomimetics against the SARS-CoV-2 main protease (M^pro^) and host proteases human cathepsin L (hCTSL) and cathepsin B (hCTSB) is an attractive strategy. These dual-mode antivirals target both viral entry and replication, which could be a suitable alternative to highly specific M^pro^ and CTS inhibitors. Herein, we examined the inhibitory activity, physicochemical and ADME properties, metabolic stability, and in vivo PK parameters of peptidomimetic inhibitors bearing a potent phenoxymethyl ketone warhead. Our compounds showed nanomolar inhibition of both M^pro^ and hCTSL/hCTSB and efficiently inhibited SARS-CoV-2 replication in cell culture. Furthermore, we studied metabolism and the impact of coadministration with the CYP-inhibitor ritonavir. Taken together, we report **1** as broad-spectrum coronavirus inhibitor with attractive properties to be pursued in *in vivo* efficacy studies.

## Graphical abstract

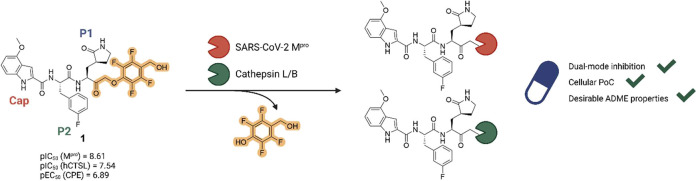

## Introduction

The COVID-19 pandemic illustrated that coronaviruses pose a serious threat to human health. The WHO has declared the SARS-CoV-2 pandemic to be over, but the need for therapeutic options remains, not only for specific (immunocompromised) patients that are currently infected, but also to enhance our preparedness for future novel coronavirus outbreaks. Vaccines have been very successful in curbing the pandemic and drastically lowering the number of severe COVID-19 cases. However, there remains a need for antivirals for those who cannot be vaccinated or risk groups that do not respond to vaccinations. More importantly, since it is uncertain whether vaccines will also be successful during a next coronavirus outbreak, it is crucial that effective broad-spectrum antivirals are available for use in prophylactic and therapeutic settings to prevent a next massive outbreak and simultaneously, gain time while vaccines are being developed. The existing landscape of effective antivirals against SARS-CoV-2 on the market is still limited.^1–3^

In antiviral drug design against coronaviruses, the viral main protease (M^pro^) is an attractive drug target, as it does not possess extensive sequence similarities with human proteases and is highly conserved among coronaviruses.^4^ M^pro^ catalytically cleaves several sites in the viral polyproteins pp1a and pp1ab, thereby being essential for viral replication.^5^ Small molecule inhibitors of M^pro^ have been proven to be efficient agents for treatment of SARS-CoV-2 infected patients.^6–9^ The M^pro^ inhibitor nirmatrelvir, known as Paxlovid when coadministered with CYP-inhibitor ritonavir, possesses high selectivity toward M^pro^ over human proteases and is the only FDA- and EMA-approved SARS-CoV-2 M^pro^ inhibitor on the market so far.^10^

SARS-CoV-2 entry requires proteolytic cleavage of the viral Spike glycoprotein (S protein) by host transmembrane protease serine 2 (TMPRSS2) when the cell surface entry pathway is used, or by lysosomal cathepsins during endosomal entry.^11–13^ Unlike the Delta and earlier virus variants, the current Omicron variant favors cathepsin-dependent entry over TMPRSS2-dependent entry due to evolution of the S protein.^14^ Elevated cathepsin L (hCTSL) and cathepsin B (hCTSB) levels have been observed in patients suffering from severe COVID-19 symptoms, which might be linked to enhanced viral infection.^15^ Inhibition of hCTSL and hCTSB has proven to effectively reduce SARS-CoV-2 replication both *in vitro* and *in vivo*, with broad-spectrum antiviral K777 being the most prominent example of a potent hCTS inhibitor, which is currently investigated in clinical trials.^16–20^

More recently, also dual-mode inhibitors that target both viral and host proteases have been proposed to inhibit SARS-CoV-2 infection, e.g. calpain inhibitor II, MPI8, calpeptin, MG-132, GC376, SM141 and SM142, which show potential in preclinical studies.^12,21–26^ Dual-mode inhibitor Olgotrelvir was found to enhance symptom recovery in clinical phase III, indicating that dual-mode inhibition is an attractive antiviral strategy.^27,28^ The major advantages of dual-mode inhibitors are that they inhibit two essential steps in the viral replication cycle – viral entry and polyprotein processing – and they might have a lower risk of development of drug resistance compared to specific M^pro^ inhibitors like Paxlovid, which is under clinical resistance surveillance.^29,30^ Both M^pro^, hCTSL and hCTSB are cysteine proteases, but their substrate specificity profiles vary: The M^pro^ active site accommodates glutamine or γ-lactam as glutamine surrogate in the S1 pocket, leucine or similar hydrophobic amino acids as P2 residues and various aliphatic and aromatic amino acids as P3 moieties.^29,31,32^ The hCTSL substrate specificity profile is mainly shaped by the P2 position, in which aromatic amino acids are preferred, while the P1, P3 and P4 positions allow a much broader variation.^33,34^

Most dual-mode inhibitors against M^pro^ and hCTSL, such as Olgotrelvir, MPI8, GC376, SM141 and SM142 contain the γ-lactam as P1 side chain and aliphatic or aromatic moieties in the P2 and P3 position.^22,26,28^ Few dual-mode inhibitors contain aliphatic amino acids, such as leucine as P1 side chains.^23,35^ The crystal structure of M^pro^ with calpain inhibitor II suggests that even methionine can be accommodated in the S1 pocket.^29^

As the landscape of antivirals against SARS-CoV-2 is still limited, we explore the potential of our peptidomimetics bearing a powerful phenoxymethyl ketone (PMK) warhead as both M^pro^ and hCTS inhibitors. Similar PMK warheads have been used in the context of protease inhibition before, but to our knowledge, this is the first time PMK inhibitors bearing hydroxymethyl, 1-hydroxyethyl or 2-hydroxypropan-2-yl in the 4 position are evaluated against SARS-CoV-2.^36–38^ Furthermore, we examined physicochemical and *in vitro* ADME properties as well as metabolic stability and *in vivo* PK parameters of our lead compounds to evaluate their potential as preclinical candidates to treat SARS-CoV-2 infections.

## Results and Discussion

The tetrafluorophenoxymethyl ketone warheads that we investigated contain hydroxymethyl, 1-hydroxyethyl or 2-hydroxypropan-2-yl in the 4 position with varying P1, P2 and cap modifications (Figure 1). Starting from commercially available (*S*)-methyl 2-((*tert*-butoxycarbonyl)­amino)-3-((*S*)-2-oxopyrrolidin-3-yl)­propanoate, chlorohomologation was performed according to literature to yield *tert*-butyl ((*S*)-4-chloro-3-oxo-1-((*S*)-2-oxopyrrolidin-3-yl)­butan-2-yl)­carbamate (**S1**, Scheme 1a).^39^

**Figure 1:**
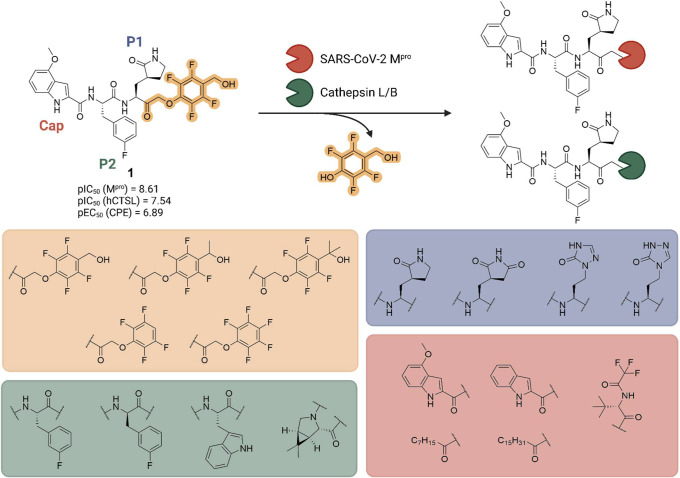
Peptidomimetic library of covalent SARS-CoV-2 M^pro^ and cathepsin L/B inhibitors bearing various phenoxymethyl ketone warheads (yellow), P1 (blue), P2 (green) and cap (red) modifications.

**Scheme 1:**
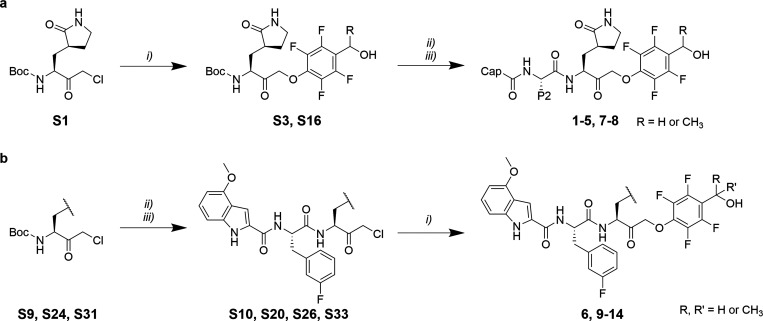
Synthesis of Peptidomimetics Bearing Phenoxymethyl Ketone Warheads with a) Varying P2/cap Moieties and b) Varying P1 Moieties^a^

a Reagents and conditions: (i) 2,3,5,6-tetrafluoro-4-(hydroxymethyl)­phenol, 2,3,5,6-tetrafluoro-4-(1-hydroxyethyl)­phenol or 2,3,5,6-tetrafluoro-4-(2-hydroxypropan-2-yl)­phenol (1.2 equiv), potassium fluoride (1.5 equiv), dry DMF, 60 °C, 16 h, 55-56%. (ii) 20% TFA/DCM, rt, 1 h, quant.; or trifluoromethanesulfonic acid (4 equiv), methoxybenzene or toluene (20 vol), MW, 100 °C, 20 min, quant. (iii) carboxylic acid (1 equiv), HATU (1.5 equiv), DIPEA (3 equiv), 0 °C→rt, 3 h. **14** has been synthesized according to scheme b), but contains a modified P2/cap building block (SI).

2,3,5,6-Tetrafluoro-4-(hydroxymethyl)­phenol was obtained by reduction of 2,3,5,6-tetrafluoro-4-hydroxybenzoic acid with BH_3_-THF as described previously.^38^ From the same starting material, 2,3,5,6-tetrafluoro-4-(1-hydroxyethyl)­phenol and 2,3,5,6-tetrafluoro-4-(2-hydroxypropan-2-yl)­phenol were obtained in six-step and eight-step synthesis routes using well-established protocols (SI). Chloromethyl ketone **S1** was subsequently coupled to the phenol of choice to yield P1/warhead building blocks **S3** and **S16**.^38^ As P2/cap building blocks, we explored 4-methoxyindoyl-3-fluorophenyl-alanine, indoyl-tryptophan, octanoyl-3-fluorophenylalanine and palmitoyl-3-fluorophenylalanine, and a commercially available nirmatrelvir intermediate consisting of a P2 bicyclic proline, P3 *tert*-leucine and trifluoroacetic acid cap. All other P2/cap building blocks **S4**–**S8** were obtained as carboxylic acids through SPPS with CTC resin. Boc-protected intermediates **S3** and **S16** were deprotected using 20% TFA in DCM, followed by solution-phase peptide coupling with the respective P2/cap carboxylic acid building blocks using HATU and DIPEA to yield final products **1–5**, **7–8**.

Next, we synthesized peptidomimetics bearing modified P1 side chains, including a succinimide with alanine spacing, and 2-linked and 4-linked 1,2,4-triazolones with homoalanine spacing (Scheme 1b). Those were obtained synthesizing first the P1 Boc-protected chloromethyl ketones **S9**, **S24** and **S31** using widely established procedures as detailed in the SI. In brief, succinimidyl alanine S9 was obtained via RuO_4_ oxidation of the γ-lactam of chloromethylketone **S1**.^40^ *N*-triazolonyl homoalanine **S24** and **S31** were obtained via substitution of commercially available *tert*-butyl (*S*)-2-((*tert*-butoxycarbonyl)­amino)-4-iodobutanoate by the appropriately protected 1,2,4-triazolone and subsequent chlorohomologation analogously to **S1**. Chloromethyl ketones **S9**, **S24** and **S31** were deprotected and used in solution-phase peptide couplings with 4-methoxy indoyl-3-fluorophenylalanine **S4**. Finally, chloromethyl ketones bearing the fully assembled P1/P2/cap peptide side chains (**S10**, **S20**, **S26**, **S33**) were coupled to different phenols to obtain the final products **6**, **9**, **11**-**13**. Compounds **10** and **14** were synthesized analogously, coupling **S4** or the nirmatrelvir carboxylic acid, respectively, to **S1**, and introducing the PMK substituent in the final step.

With the desired library of peptidomimetics in hand, we evaluated the activities of our molecules in biochemical and cell-based assays, using nirmatrelvir as well as earlier reported compounds **15** and **16** as controls.^36^ In biochemical assays, enzymatic activities of SARS-CoV-2 M^pro^, hCTSL, hCTSB, mouse cathepsin L (mCTSL), mouse cathepsin B (mCTSB), and hamster CTSL were evaluated (Table 1, Table S2). Cell-based reporter assays were performed in 293T cells (M^pro^ reporter assay) and VeroE6 cells (entry reporter assay) using quantification of luciferase activity as a readout for protease activity. Finally, the antiviral efficacy and cytotoxicity of our compounds were evaluated in SARS-CoV-2 CPE reduction assays using VeroE6 cells (Table 1, Table S1, Figure S1). Most of our compounds were found to inhibit both M^pro^ and hCTSL with nanomolar IC_50_ values except for **2** and **4**, which showed only micromolar inhibition. Inhibitor **1**, which contains 4-methoxyindoyl-3-fluorophenylalanine in the P2/cap position, was found most active against both M^pro^ (pIC_50_ = 8.61), hCTSL (pIC_50_ = 7.64) and hCTSB (pIC_50_ = 6.36). Its activity was confirmed in the M^pro^ reporter assay (pEC_50_ = 6.98) and entry reporter assay (pEC_50_ = 5.60). In the cell-based antiviral (CPE reduction) assay with SARS-CoV-2 on Vero E6 cells **1** was found to be highly active (pEC_50_ = 6.89). To gain information on broad-spectrum antiviral activity, we assessed the activity of **1** against other coronaviruses. Herein, **1** was found to be active against SARS-CoV with a slightly higher EC_50_ (pEC_50_ = 6.52). Remarkably, **1** was also found to be very active against MERS-CoV in huh-7 cells with single digit nanomolar EC_50_ (pEC_50_ = 8.28), which underlines its robustness and broad-spectrum applicability.

**Table 1:**
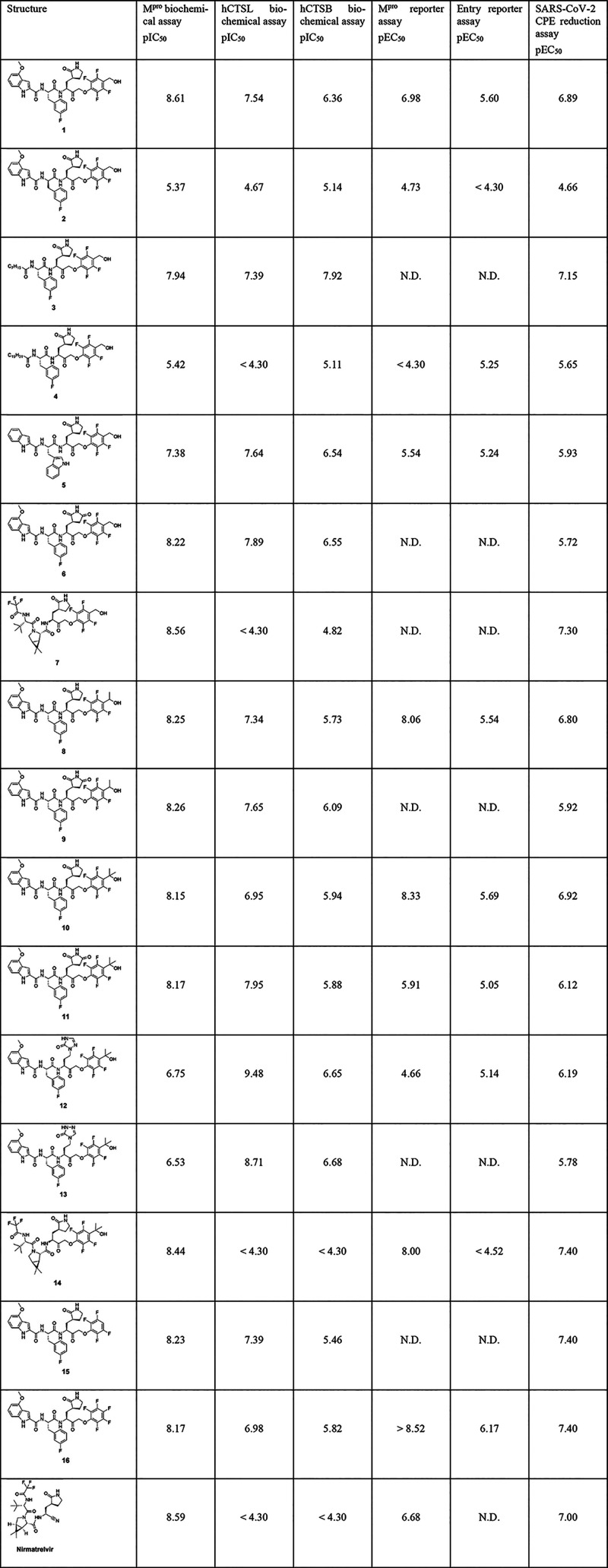
Combined Biochemical and Cellular Activity Data of Our Peptidomimetic Library^a^

a Activity data of mCTSL, mCTSB and hamster CTSL are depicted in Table S2 (N.D. = No data).

Compared to inhibitor **1**, the stereoisomer containing *D*-3-fluorophenylalanine (**2**) was ∼ 169-fold less active against SARS-CoV-2 in the CPE reduction assay. The same trend was observed in the cellular and biochemical assays, pointing out the importance of stereochemistry when assessing peptidomimetic inhibitors against M^pro^ and hCTS.

Synthesizing compounds **3** and **4**, we explored the potential of aliphatic chains in the cap position, which to our knowledge has not been reported before. Herein, **3** with the shorter octanoyl tail proved to be more potent than palmitoyl-derivative **4** and showed almost equal potency as **1**. Next, we introduced variations at the warhead position, replacing the hydroxymethyl moiety by 1-hydroxyethyl (**8**) and 2-hydroxypropan-2-yl (**10**). Both analogs were equally active as **1** in all activity assays (pEC_50_ (CPE) = 6.80 and pEC_50_ (CPE) = 6.92, respectively), showing a slight preference for the tertiary alcohol over the primary and secondary ones. To compare our peptidomimetic library to known M^pro^ and dual-mode inhibitors, we took along reference compounds **15** and **16**, bearing similar PMK warheads and nirmatrelvir in activity assays. Inhibitors **15** and **16** contain the 4-methoxyindoyl and 3-fluorophenylalanine, which we hypothesized to result in dual-mode inhibitory activity. The pEC_50_ and pIC_50_ values of both of these analogs in all activity assays were in a similar range as our most potent inhibitors. A clear dual inhibitory mode for both M^pro^ and hCTSL could be verified with ∼ 7- and 16-fold selectivity for M^pro^ over hCTSL, respectively. In our nirmatrelvir analogs, we replaced the nitrile warhead by 2,3,5,6-tetrafluoro-4-(hydroxymethyl)­phenoxymethyl ketone (**7**) and 2,3,5,6-tetrafluoro-4-(2-hydroyxpropan-2-yl)­phenoxymethylketone (**14**). Due to their optimized P2/P3/cap groups derived from nirmatrelvir, **7** and **14** are highly selective M^pro^ inhibitors. They were found equally active in the biochemical M^pro^ assay, while no hCTSL and hCTSB activity was observed, and they were ∼ 2-fold more active than nirmatrelvir in the SARS-CoV-2 CPE reduction assay (pEC_50_ = 7.30 and pEC_50_ = 7.40, respectively).

We predicted the Michaelis complex of **1** with M^pro^, hCTSL and hCTSB using molecular modeling to assess whether the PMK warhead has an enthalpic role to binding (Figure S2). For all complexes, the PMK warhead does form hydrophobic contacts, but the solvent-exposed nature of the majority of the phenyl ring in the complexes and electrostatic nature of the S1’ pockets likely decreases the solvation energy gain. Apart from hydrophobic contacts, the PMK moiety is predicted to engage in some directional interactions, such as a potential π-amide interaction with the Cys22 backbone and/or Gln19 backbone in hCTSL, and a hydrogen bond between the hydroxymethyl and His111 in hCTSB. Furthermore, the P3–P1 moieties in the predicted Michaelis complexes follow previously observed trends and interactions for each enzyme. For instance, the P1 γ-lactam moiety is solvent-exposed in the predicted hCTSL complex, which is well reflected in the template protein:ligand complex used for modeling (PDB ID: 8GX2), the protease’s specificity from MEROPS and the allowance of a tryptophan residue as P1 moiety.^41,42^ Similarly, the P3-indoyl cap engages in π-amide interactions with the Gly68 and Gly73 backbone for hCTSL and hCTSB, respectively.^43^ Although our predicted Michaelis complexes indicate some gain in affinity for the PMK warhead with an increasing trend from Mpro ≪ hCTSL < hCTSB, we hypothesize that the electron-withdrawing nature of the PMK warhead is the major driver of potency. Previous kinetics studies argue that inactivation by aryloxymethyl ketones is strongly dependent on the leaving group p*K*_a_.^44–46^

To rule out off-target effects, we investigated the activities of our most potent inhibitors against a diverse panel of host proteases. Herein, we screened compounds **1**, **8** and **14** against calpain-1, caspase 2, cathepsin D, neutrophil elastase 2, thrombin and trypsin at a concentration of 10 μM. Only limited activity was found against the selected off-target proteases, indicating high % selectivity (Table S3).

Having established a clear dual-mode of action for **1** and **8**, we continued to explore our peptidomimetic inhibitors in physicochemical and *in vitro* ADME studies. We investigated LogD, solubility, mouse plasma protein binding, as well as metabolic stability (Table 2, Table S4).

**Table 2:** Physicochemical and *In Vitro* ADME Properties of Selected Compounds

compound	LogD	kinetic solubility [μM]	mouse plasma protein binding	CL_int_ (mouse hepatocytes) [μL/min/10^6^ cells]
**1**	1.65	18.0	98.06% Fb 1.94% Fu	185.8
**3**	2.23	0.00	99.59% Fb 0.41% Fu	206.3
**4**	>3.5	0.00	N.D.	9.31
**5**	1.50	0.00	98.79% Fb 1.21% Fu	181.7
**6**	3.08	20.9	N.D.	155.5
**7**	2.82	71.3	96.45% Fb 3.55% Fu	121.2
**8**	3.26	12.3	99.17% Fb 0.83% Fu	64.5
**9**	2.60	6.33	N.D.	133.6
**10**	3.15	3.65	98.95% Fb 1.05% Fu	47.6
**11**	2.03	0.88	N.D.	124.3
**12**	2.88	5.84	99.69% Fb 0.31% Fu	76.8
**13**	2.70	6.78	99.69% Fb 0.28% Fu	78.8
**14**	2.52	77.2	99.24% Fb 0.76% Fu	112.1
**15**	2.99	1.40	99.57% Fb 0.43% Fu	240.3
**16**	2.95	4.09	99.33% Fb 0.67% Fu	153.7

In mouse, hamster and human microsome stability, as well as mouse and human hepatocyte stability, we found that most compounds suffered from poor metabolic stability (Tables S4 and S5). Inhibitor **10**, bearing the 2-hydroxypropan-2-yl warhead, was found the most stable analog with CL_int_ = 47.6 μL/min/10^6^ cells in mouse hepatocytes, meaning that the trend tertiary > secondary > primary alcohol applies in terms of metabolic stability. Kinetic solubility assays revealed that compounds **3**, **4** and **5**, containing aliphatic fatty acid caps and the aromatic indoyl-tryptophan, were unfortunately insoluble in water. Inhibitor **1**, containing 4-methoxyindoyl-3-fluorophenylalanine instead, was found to be soluble up to 18.0 μM, but was significantly less soluble than both nirmatrelvir analogs **7** and **14**. Comparing solubilities among warhead analogs **1**, **8** and **10**, the reverse trend primary > secondary > tertiary alcohol applies. Given that the primary and tertiary alcohol warhead analogs were slightly more active in the CPE reduction assay, **1** is our preferred choice with regard to both, dual-mode activity and solubility.

To gain further insights into the metabolic stability of exemplary compound **1**, we characterized arising metabolites at 0, 5, 15, 30, 45, and 60 min by LCMS in the mouse microsomal stability assay. Herein, three points of metabolism were observed: P2/cap oxidation (M1), P1 oxidation (M2) and P1 ketone formation (M3) through further oxidation. Within the first 30 min, M2 was found to be the major metabolite, at 45 min, M2 and M3 were in equal amounts present and after 1 h, M3 was detected as the major metabolite (Table S6).

Metabolism is hypothesized to be CYP-mediated. Taking into account literature findings, we conclude that the P1 γ-lactam is oxidized to the corresponding hydroxy-γ-lactam (M2).^47,48^ Unlike others, we also observed further oxidation of the hydroxy-γ-lactam moiety over time to the corresponding succinimide under our conditions (M3, Scheme S1).

Being aware of CYP-mediated oxidation, we followed several approaches to improve the metabolic stability of our inhibitors. First, we synthesized the observed metabolite M3, **6**, and similar analogs, in which the P1 γ-lactam was replaced by succinimide. Compounds **6**, **9** and **11** were synthesized starting from *tert*-butyl ((*S*)-4-chloro-3-oxo-1-((S)-2-oxopyrrolidin-3-yl)­butan-2-yl)­carbamate, which was oxidized using sodium periodate and ruthenium­(III) chloride. After that, the synthetic route described in Scheme 1 was followed.

In the biochemical M^pro^ and hCTS assays, we report nanomolar activities for all three succinimide analogs with a slight preference for M^pro^ over hCTSL. However, our panel of succinimides did not perform as well in cellular activity assays as the respective γ-lactam parent compounds. Contrary to the γ-lactam parent compounds, activities in cell-based assays varied from pEC_50_ = 5.72 to pEC_50_ = 6.12. The most active succinimide analog is **11**, which was found to be ∼ 6-fold less active than **1** in the SARS-CoV-2 antiviral assay. Interestingly, we observed a clear trend in activity (primary > secondary > tertiary alcohol) that was not in line with the earlier observed trend applying to γ-lactam parent compounds. Even though P1 γ-lactam oxidation was found to be the major structural point of metabolic instability, mouse hepatocyte stability studies of **6** revealed only a slight increase in clearance with CL_int_ = 155.5 μL/min/10^6^ cells when compared to **1** (CL_int_ = 185.8 μL/min/10^6^ cells). We hypothesize that oxidation of the P2/cap side chains contributes to metabolic stability to a higher extent than anticipated.

Second, we explored replacing the γ-lactam by 4-linked and 2-linked 1,2,4-triazolones – **12** and **13**, respectively – with homoalanine spacing as new P1 modifications. We hypothesize that bound to the S1 pocket of M^pro^, the carbonyl will undergo hydrogen bonding with His163 and the triazolone NH will undergo hydrogen bonding with Glu166 (Figure S3). Both compounds were found less active than inhibitors bearing the P1 γ-lactam, with the 4-linked 1,2,4-triazolone being more active in both cellular and biochemical M^pro^ and hCTSL assays compared to the 2-linked 1,2,4-triazolone. Contrary to the γ-lactam library, both triazolone derivatives showed high selectivity for hCTS over M^pro^. Triazolone **12** showed more than 500-fold selectivity for hCTSL over M^pro^ and is the most potent hCTSL inhibitor reported in this work with pIC_50_ = 9.48. Nevertheless, both compounds did not effectively inhibit SARS-CoV-2 infection in the CPE assay with pEC_50_ = 5.78 and pEC_50_ = 6.19, respectively. Therefore, we suggest the further exploration of triazolone-containing peptidomimetics and their potential to target hCTS and to inhibit hCTS functions in a different biochemical context.

As these structural alterations did not substantially improve metabolic stability, we investigated coadministration with a CYP3A4 inhibitor. Pfizer and others have reported that CYP3A4 inhibitor ritonavir effectively improves the metabolic stability of antivirals including nirmatrelvir.^48,49^ As a proof of concept, hepatocyte stability assays were performed in the presence of ritonavir with a 4:1 ratio (compound of interest/ritonavir). We performed stability assays not only in mouse, but also in hamster hepatocytes, as pathogenesis and clinical features of SARS-CoV-2 infected K18-hACE2 mice and Syrian golden hamsters tend to vary (Table 3, Table S7).^50^

**Table 3:** Hamster Hepatocyte Stability Data for Selected Compounds with and without Coadministration of Ritonavir

	hamster hepatocyte stability		hamster hepatocyte stability + ritonavir 4:1	
compound	CL_int_ [μL/min/10^6^ cells]	*t*_1/2_ [min]	CL_int_ [μL/min/10^6^ cells]	*t*_1/2_ [min]
**1**	87.6	7.9	77.1	9.0
**7**	50.2	13.8	41.9	16.6
**8**	33.1	20.9	29.7	23.3
**10**	27.5	25.2	19.4	35.7
**14**	62.1	11.2	44.7	15.5

We assessed a few compounds of interest and found that hamster hepatic clearance ranged from CL_int_ = 27.5 μL/min/10^6^ cells (*t*_1/2_ = 25.2 min) to CL_int_ = 87.6 μL/min/10^6^ cells (*t*_1/2_ = 7.9 min). Coadministration of ritonavir in a 4:1 ratio improved the hepatic stability slightly, with clearances ranging from CL_int_ = 19.4 μL/min/10^6^ cells (*t*_1/2_ = 35.7 min) to CL_int_ = 77.1 μL/min/10^6^ cells (*t*_1/2_ = 9.0 min) (Table 3). Generally, the effect of ritonavir on hepatic clearance was more pronounced in mouse than in hamster hepatocytes. For example, in mouse hepatocytes, the clearance of **7** decreased from CL_int_ = 121.2 μL/min/10^6^ cells (*t*_1/2_ = 14.3 min) without coadministration to CL_int_ = 21.8 μL/min/10^6^ cells (*t*_1/2_ = 79.5 min) in the presence of ritonavir (Table S5). In hamster hepatocytes, stability was assessed using varying concentrations of ritonavir ranging from 4:1 to 1:1 ratios (compound of interest/ritonavir). Changing the ratio first to 2:1 and then to 1:1, we observed a clear concentration dependence with increasing hepatic stability (Table S7). Finally, we performed hepatocyte stability assays with inhibitors **1** and **14** in human hepatocytes (Table S5). Both compounds showed moderate clearance with CL_int_ = 12.5 μL/min/10^6^ cells (*t*_1/2_ = 55.5 min) and CL_int_ = 6.6 μL/min/10^6^ cells (*t*_1/2_ = 105.3 min), respectively, even without the addition of ritonavir. Upon coadministration, metabolic stability was significantly improved, resulting in CL_int_ = 6.9 μL/min/10^6^ cells (*t*_1/2_ = 100.3 min) and even CL_int_ = 0.65 μL/min/10^6^ cells (*t*_1/2_ > 120 min) in the case of **14**. Based on this increased metabolic stability in human hepatocytes, we hypothesize that coadministration of ritonavir might only be required in mouse and hamster studies. Taken together, we expect that the addition of a CYP3A4 inhibitor such as ritonavir should enable the successful evaluation of our compounds in an animal infection model.

Next, we studied compounds **1**, **7**, **8** and **14** in *in vivo* PK studies, using 6–8 weeks old female Syrian golden hamsters and administering ritonavir orally 30 min prior to dosing (Figure 2, Table S8). Unfortunately, oral dosing did not result in sufficient bioavailability, as the total exposure was too low for all four compounds. Instead, intraperitoneal and subcutaneous were found the preferred routes of administration. Generally, **7** and **8** showed significantly lower bioavailability compared to **1** and **14**. The most favorable PK parameters have been observed for **14**, as this compound showed higher plasma concentrations than **1** in all administration routes. However, upon 100 mg/kg intraperitoneal dosing of **14**, clinical signs were reported, as the animals were found dull and lethargic 15 min up until 45 min postdosing.

**Figure 2:**
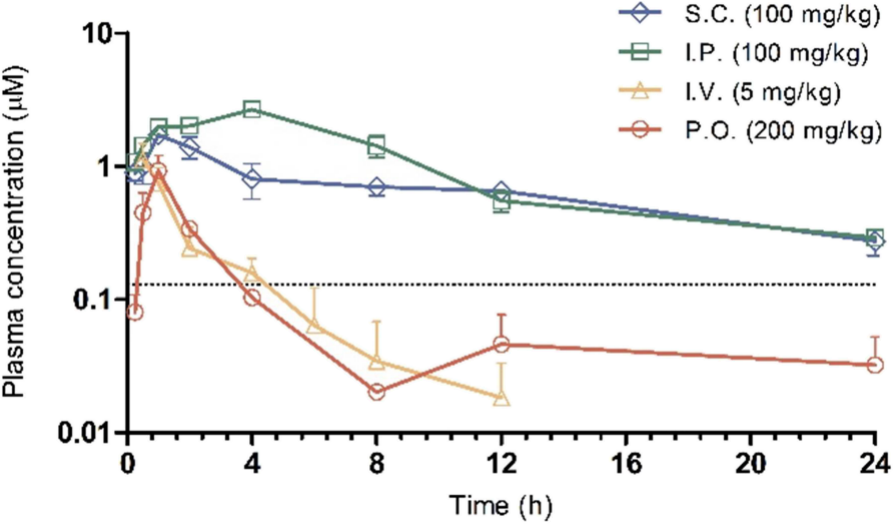
Mean plasma concentration (μM) of **1** pretreated orally with ritonavir (100 mg/kg) in female Syrian golden hamster following subcutaneous (100 mg/kg), intraperitoneal (100 mg/kg), intravenous (5 mg/kg) and oral (200 mg/kg) administration (*n* = 3). Dotted line: EC_50_ (CPE) = 130 nM.

**1** showed sufficiently high plasma concentrations over the course of 24 h upon intraperitoneal and subcutaneous dosing, especially when taking its antiviral efficacy in VeroE6 cells into account (Figure 2, dotted line). Opposed to compound **14**, no clinical signs have been observed upon intraperitoneal dosing. The data suggests proceeding with multiday PK studies and subsequently, *in vivo* efficacy studies of **1**, either applying a dosing of 100 mg/kg subcutaneous once a day, or 50 mg/kg subcutaneous twice a day.

## Conclusions

Herein, we report a library of novel peptidomimetic M^pro^ and hCTS inhibitors bearing phenoxymethyl ketone warheads. These dual-mode inhibitors were highly active in biochemical and cell-based SARS-CoV-2 infection assays, and they show broad-spectrum activity against SARS-CoV and MERS-CoV. While most compounds predominantly inhibited M^pro^, we also report two novel selective hCTSL inhibitors. Furthermore, we explored physicochemical and *in vitro* ADME properties of selected compounds, revealing moderate solubility, favorable plasma protein binding and fast clearance in mouse hepatocytes. Coadministration with CYP3A4 inhibitor ritonavir enhanced metabolic stability in mouse, hamster and human hepatocytes and microsomes. We hypothesize that coadministration of ritonavir will be needed in further *in vivo* efficacy, safety and tolerability studies of **1** in animal models. *In vivo* PK studies in Syrian golden hamsters of **1** coadministered with ritonavir indicated sufficiently high plasma concentrations upon 100 mg/kg subcutaneous and 100 mg/kg intraperitoneal dosing to proceed toward *in vivo* efficacy studies. Collectively, we report **1** as an attractive antiviral drug candidate for further *in vivo* studies against SARS-CoV-2.

## Experimental Section

### General Synthetic and Analytical Methods

NMR spectra were recorded on a Bruker Avance III 400 MHz or a Bruker 500 MHz spectrometer and the compounds were assigned using ^1^H NMR, ^13^C NMR, ^19^F NMR, COSY, HSQCED and HMBC spectra. Chemical shifts were reported in parts per million (ppm.) relative to reference (CDCl_3_: ^1^H: 7.26 ppm and ^13^C: 77.16 ppm; CD_3_OD: ^1^H: 3.31 ppm and ^13^C: 49.00 ppm; (CD_3_)_2_SO: ^1^H: 2.50 ppm and ^13^C: 39.52 ppm.) NMR data are presented in the following way: chemical shift, multiplicity (s = singlet, bs = broad singlet, d = doublet, t = triplet, dd = doublet of doublets, ddd = doublet of doublet of doublets, dtd = doublet of triplet of doublets h = heptet, m = multiplet and/or multiple resonances) and coupling constants *J* in Hz. Mass spectra were recorded on a JEOL AccuTOF CS JMS-T100CS (ESI)­mass spectrometer. Automatic flash column chromatography was executed on a Biotage Isolera Spektra One using SNAP or Silicycle cartridges (Biotage, 30–100 μm, 60Å) 4–50 g. Reactions under protective atmosphere were performed under positive Ar./N2 flow in flame-dried flasks. Purity of final compounds was determined by analytical HPLC (Waters, Protenovi C4 column, 4.6 × 250 mm, 5 μM OR XBridge C18 column, 4.6 × 150 mm, 3.5 μM OR Kinetex C18 column, 50 × 2.1 mm, 1.7 μM; A: 10 mM ammonium acetate in MQ, B: 100% MeCN; A:B 1/1; 1.0 mL/min; 60 °C). All compounds are >95% pure as determined by analytical HPLC.

#### General Procedure 1

Chloromethylketone coupling to phenol. Chloromethylketone (1 equiv) was dissolved in dry DMF (20 vol), followed by addition of phenol (1.2 equiv) and potassium fluoride (1.5 equiv). The reaction mixture was stirred for 16 h at 60 °C. The reaction mixture was then diluted with water and the aqueous layer was extracted with EtOAc (3 × 25 mL). The combined organic layer was washed with sat. NaCl, dried over Na_2_SO_4_, filtered, and concentrated *in vacuo*. Unless stated otherwise, the crude product was purified by flash column chromatography (0→5% MeOH in DCM).

#### General Procedure 2

Boc deprotection. Boc-protected amino acid (1.1 equiv) was dissolved in dry DCM (20 vol) and the solution was cooled in an ice bath, followed by the dropwise addition of TFA (20%/DCM). The reaction mixture was stirred for 1 h at room temperature. Subsequently, the reaction mixture was coevaporated with diethyl ether (5 × 5 mL), dried *in vacuo* and the obtained TFA salt was used without further purification.

#### General Procedure 3

Peptide coupling. Carboxylic acid (1 equiv) was introduced in a three-necked round-bottomed flask, after which three vacuum-backfill cycles were performed. Subsequently, peptide-grade DMF (20 vol) was added, and the solution was cooled in an ice bath. Then, HATU (1.5 equiv) and DIPEA (3 equiv) were added, and the reaction mixture was stirred for 20 min at 0 °C. Lastly, Boc-deprotected TFA salt (1.1 equiv) was added and the reaction mixture was stirred at room temperature for 3 h. The reaction mixture was poured into sat. aq. NH_4_Cl (20 mL) and extracted with EtOAc (3 × 20 mL). The combined organic layers were washed with sat. aqueous NaHCO_3_ (2 × 50 mL), water (2 × 50 mL) and brine (2 × 50 mL), dried over Na_2_SO_4_, filtered and concentrated *in vacuo.* Unless stated otherwise, the crude product was purified by flash column chromatography (0→5% MeOH in DCM).

#### General Procedure 4

Solid Phase Peptide Synthesis of dipeptide acids. A solution of Fmoc-protected amino acid (3.0 equiv) and DIPEA (5.0 equiv) in DMF (10 vol) was added to 2-chloro CTC resin (10 g, original loading rate: 1.2 mmol/g) and gently agitated under nitrogen bubbling for 16 h. Reagents were drained, and the resin was washed with DMF (2 × 10 vol), IPA (2 × 10 vol) and DMF (2 × 10 vol) sequentially for each 5 min. The unreacted chlorides in 2-CTC resin were capped with MeOH/DIPEA/DMF (15/5/80) for 15 min. The reagents were drained and washed with DCM. Subsequent Fmoc deprotection was performed with a mixture of 20% piperidine in DMF (10 vol) for 2 × 10 min by gently agitating under nitrogen bubbling. The mixture was drained and washed with DMF (2 × 10 vol), IPA (2 × 10 vol) and finally with DMF (2 × 10 vol). A solution of Fmoc-protected amino acid (3.0 equiv), Oxyma (3.0 equiv) and DIC (4.0 equiv) in DMF (10 vol) was added to the above resin and gently agitated under nitrogen bubbling for 2 h. Completion of coupling was monitored by Kaiser test. Upon a negative test result, reagents were drained, and the resin was washed with DMF (2 × 10 vol), IPA (2 × 10 vol) and finally with DCM (2 × 10 vol) for each 5 min. The dipeptide was cleaved off the resin using 30% HFIP in DCM as cleavage cocktail (10 vol) for 2 × 30 min, filtered and washed the resin with DCM (10 vol). The combined filtrates were concentrated under vacuum. The obtained crude peptide mass was triturated with diethyl ether to get off-white solid. It was filtered, washed two times with diethyl ether followed by drying under vacuum for 2 h, yielding the desired dipeptide.

##### *tert*-Butyl ((*S*)-4-Chloro-3-oxo-1-((*S*)-2-oxopyrrolidin-3-yl)­butan-2-yl)­carbamate (**S1**)

A three-necked flame-dried flask (100 mL) equipped with a nitrogen inlet and internal thermometer was charged with (*S*)-methyl 2-((*tert*-butoxycarbonyl)­amino)-3-((*S*)-2-oxopyrrolidin-3-yl)­propanoate (500 mg, 1.75 mmol, 1 equiv), chloroiodomethane (507 μL, 6.98 mmol, 4 equiv), and dry THF, and the solution was cooled to −77 °C. Lithium diisopropylamide (2 M, 5.24 mL, 10.5 mmol, 6 equiv) in THF/hexane was added via a pressure-equalizing dropping funnel at such a rate to keep the internal temperature below −70 °C. After complete addition, the reaction mixture was stirred for another hour, before quenching with acetic acid (800 μL, 14.0 mmol, 8 equiv) in THF (5 mL), over 20 min, while maintaining the temperature below −70 °C. The reaction mixture was diluted with EtOAc and water. The organic layer was collected and washed with water, sat. NaHCO_3_ and brine. The combined organic layers were dried over Na_2_SO_4_, filtered, and concentrated *in vacuo*. The crude product was purified by flash column chromatography (20% acetone in toluene) to give **S1** as a brown oil (176 mg, 33%). R*_f_* = 0.3 (acetone/toluene 1:1). ^1^H NMR (500 MHz, *d*-DMSO) δ 7.65 (s, 1H), 7.52 (d, *J* = 7.6 Hz, 1H), 4.61 (ABq, *J* = 23.6 Hz, 2H), 4.16 (ddd, *J* = 11.3, 7.6, 4.0 Hz, 1H), 3.20–3.09 (m, 2H), 2.29–2.19 (m, 1H), 2.18–2.09 (m, 1H), 1.87 (ddd, *J* = 13.9, 10.9, 4.6 Hz, 1H), 1.71–1.56 (m, 2H), 1.38 (s, 9H). HRMS (*m*/*z*): [M + H]^+^ calcd for C_13_H_21_ClN_2_O_4_: 305.1263; found: 305.1247.

##### 2,3,5,6-Tetrafluoro-4-(hydroxymethyl)­phenol (**S2**)

2,3,5,6-tetrafluoro-4-hydroxybenzoic acid (500 mg, 2.38 mmol, 1 equiv) was dissolved in anhydrous THF (2 mL). BH_3_THF (1 M, 9.52 mmol, 4 equiv) was added dropwise and the reaction was heated to reflux for 16 h. The reaction mixture was quenched with 2 N HCl, diluted with water (50 mL) and extracted with EtOAc (3 × 50 mL). The organics were combined, washed with sat. NaCl, dried over Na_2_SO_4_, filtered, and concentrated *in vacuo*. The crude product was purified by flash column chromatography (10→50% EtOAc in *n*-heptane) to yield **S2** as a white solid (392 mg, 84%). R*_f_* = 0.3 (EtOAc/heptane 2:1). ^1^H NMR (400 MHz, *d*-MeCN) δ 4.61 (t, *J* = 1.8 Hz, 2H). HRMS (*m*/*z*): [M - H]^−^ calcd for C_7_H_3_F_4_O_2_: 195.0075; found: 195.0066.

##### *tert*-Butyl ((*S*)-3-Oxo-1-((*S*)-2-oxopyrrolidin-3-yl)-4-(2,3,5,6-tetrafluoro-4-(hydroxymethyl)­phenoxy)­butan-2-yl)­carbamate (**S3**)

According to GP1 starting from *tert*-butyl ((*S*)-4-chloro-3-oxo-1-((*S*)-2-oxopyrrolidin-3-yl)­butan-2-yl)­carbamate (130 mg, 0.43 mmol) and 2,3,5,6-tetrafluoro-4-(hydroxymethyl)­phenol (100 mg, 0.51 mmol), **S3** was obtained as a white solid (109 mg, 55%). R*_f_* = 0.5 (MeOH/DCM 1:9). ^1^H NMR (400 MHz, *d*-DMSO) δ 7.65 (s, 1H), 7.70 (d, *J* = 7.7 Hz, 1H), 5.48 (t, *J* = 5.81 Hz, 1H), 5.22 (ABq, 2H), 4.51 (dt, *J* = 5.87, 1.77 Hz, 2H), 4.12 (ddd, *J* = 11.47, 7.61, 4.23 Hz, 1H), 3.20–3.08 (m, 2H), 2.29–2.18 (m, 1H), 2.18–2.09 (m, 1H), 1.93–1.83 (m, 1H), 1.70–1.53 (m, 2H), 1.39 (s, 9H). ^19^F NMR (377 MHz, *d*-DMSO) δ −146.39 (dd, *J* = 23.2, 8.8 Hz), −157.49 (dd, *J* = 23.2, 8.8 Hz). HRMS (*m*/*z*): [M + H]^+^ calcd for C_20_H_24_F_4_N_2_O_6_: 465.1643; found: 465.1655.

##### (*S*)-3-(3-Fluorophenyl)-2-(4-methoxy-1*H*-indole-2-carboxamido)­propanoic acid (**S4**)

According to GP4 coupling using 2-chloro CTC resin (5.0 g, 1.2 mmol/g) with Fmoc-3-fluoro-l-phenylalanine (3.0 equiv) and 4-methoxy-1*H*-indole-2-carboxylic acid (2.0 equiv), dipeptide **S4** was obtained as an off-white solid (2.0 g, 93%), which was used directly without further purification. ^1^H NMR (500 MHz, *d*-DMSO) δ 12.83 (bs, 1H), 11.51 (d, *J* = 2.4, 1H), 8.66 (d, *J* = 8.4 Hz, 1H), 7.33–7.26 (m, 2H), 7.20–7.14 (m, 2H), 7.09 (t, *J* = 7.9 Hz, 1H), 7.02–6.95 (m, 2H), 6.50 (d, *J* = 7.7 Hz, 1H), 4.65 (ddd, *J* = 10.8, 8.3, 4.3 Hz, 1H), 3.88 (s, 3H), 3.22 (dd, *J* = 13.8, 4.4, 1H), 3.07 (dd, *J* = 13.9, 10.8 Hz, 1H). ^19^F NMR (471 MHz, *d*-DMSO) δ −113.80. HRMS (*m*/*z*): [M - H]^−^ calcd for C_19_H_16_FN_2_O_4_: 355.1100; found: 355.1109.

##### *N*-((*S*)-3-(3-Fluorophenyl)-1-oxo-1-(((*S*)-3-oxo-1-((*S*)-2-oxopyrrolidin-3-yl)-4-(2,3,5,6-tetrafluoro-4-(hydroxymethyl)­phenoxy)­butan-2-yl)­amino)­propan-2-yl)-4-methoxy-1*H*-indole-2-carboxamide (**1**)

*tert*-Butyl ((*S*)-3-oxo-1-((*S*)-2-oxopyrrolidin-3-yl)-4-(2,3,5,6-tetrafluoro-4-(hydroxymethyl)­phenoxy)­butan-2-yl)­carbamate (287 mg, 0.62 mmol, 1.1 equiv) was deprotected following GP2. Subsequently, GP3 was followed using (*S*)-3-(3-fluorophenyl)-2-(4-methoxy-1*H*-indole-2-carboxamido)­propanoic acid (200 mg, 0.56 mmol) and (*S*)-3-((*S*)-2-amino-3-oxo-4-(2,3,5,6-tetrafluoro-4-(hydroxymethyl)­phenoxy)­butyl)­pyrrolidin-2-one (TFA salt) (295 mg, 0.62 mmol) and the crude product was purified by RP-HPLC to yield **1** (33 mg, 8%). ^1^H NMR (400 MHz, *d*-DMSO) δ 11.52 (d, *J* = 2.3 Hz, 1H), 8.67 (dd, *J* = 17.6, 8.1 Hz, 2H), 7.63 (s, 1H), 7.35–7.16 (m, 4H), 7.08 (t, *J* = 8.0 Hz, 1H), 7.01–6.91 (m, 2H), 6.50 (d, *J* = 7.7 Hz, 1H), 5.12 (ABq, *J* = 48.8 Hz, 2H), 4.76–4.66 (m, 1H), 4.50 (s, 2H), 4.48–4.43 (m, 1H), 3.89 (s, 3H), 3.18–2.99 (m, 4H), 2.31–2.21 (m, 1H), 2.12–1.93 (m, 2H), 1.69–1.55 (m, 2H), 0.89–0.75 (m, 1H). ^13^C NMR (126 MHz, *d*-DMSO) δ 203.3, 178.2, 171.9, 162.0 (d, *J* = 243.0 Hz), 161.2, 153.6, 145.8 (m), 143.8 (m), 141.1 (d, *J* = 7.5 Hz), 140.6 (d, *J* = 15.8 Hz), 138.6 (d, *J* = 16.3 Hz), 137.8, 135.7 (m), 129.9 (d, *J* = 8.4 Hz), 129.6, 125.3 (d, *J* = 2.6 Hz), 124.5, 118.0, 115.9 (d, *J* = 21.1 Hz), 113.1 (d, *J* = 21.1), 112.8 (m), 105.4, 101.0, 99.2, 74.9, 55.0, 54.5, 53.7, 50.6, 37.2, 36.5, 30.6, 27.1. ^19^F NMR (377 MHz, *d*-DMSO) δ −115.1 (m), −147.5 (dd, *J* = 23.1, 8.7 Hz), −158.8 (dd, *J* = 23.4, 8.8 Hz). HRMS (*m*/*z*): [M + H]^+^ calcd for C_34_H_31_F_5_N_4_O_7_: 703.2186; found: 703.2169.

##### (*R*)-3-(3-Fluorophenyl)-2-(4-methoxy-1*H*-indole-2-carboxamido)­propanoic Acid (**S5**)

According to GP4 coupling using 2-chloro CTC resin (1.0 g, 1.2 mmol/g) with Fmoc-3-fluoro-d-phenylalanine (3.0 equiv) and 4-methoxy-1*H*-indole-2-carboxylic acid (2.0 equiv), dipeptide **S5** was obtained as an off-white solid (400 mg, 93%), which was used directly without further purification. ^1^H NMR (500 MHz, *d*-DMSO) δ 12.83 (bs, 1H), 11.51 (d, *J* = 2.3 Hz, 1H), 8.66 (d, *J* = 8.4 Hz, 1H), 7.33–7.25 (m, 2H), 7.20–7.13 (m, 2H), 7.09 (t, *J* = 7.9 Hz, 1H), 7.03–6.95 (m, 2H), 6.50 (d, *J* = 7.7 Hz, 1H), 4.65 (ddd, *J* = 11.0, 8.4, 4.3 Hz, 1H), 3.88 (s, 3H), 3.22 (dd, *J* = 13.9, 4.4 Hz, 1H), 3.07 (dd, *J* = 13.8, 10.9 Hz, 1H). ^19^F NMR (471 MHz, *d*-DMSO) δ −113.80. HRMS (*m*/*z*): [M - H]^−^ calcd for C_19_H_16_FN_2_O_4_: 355.1100; found: 355.1109.

##### *N*-((*R*)-3-(3-Fluorophenyl)-1-oxo-1-(((*S*)-3-oxo-1-((*S*)-2-oxopyrrolidin-3-yl)-4-(2,3,5,6-tetrafluoro-4-(hydroxymethyl)­phenoxy)­butan-2-yl)­amino)­propan-2-yl)-4-methoxy-1*H*-indole-2-carboxamide (**2**)

*tert*-Butyl ((*S*)-3-oxo-1-((*S*)-2-oxopyrrolidin-3-yl)-4-(2,3,5,6-tetrafluoro-4-(hydroxymethyl)­phenoxy)­butan-2-yl)­carbamate (287 mg, 0.62 mmol, 1.1 equiv) was deprotected following GP2. Subsequently, GP3 was followed using (*R*)-3-(3-fluorophenyl)-2-(4-methoxy-1*H*-indole-2-carboxamido)­propanoic acid (200 mg, 0.56 mmol) and (*S*)-3-((*S*)-2-amino-3-oxo-4-(2,3,5,6-tetrafluoro-4-(hydroxymethyl)­phenoxy)­butyl)­pyrrolidin-2-one (TFA salt) (295 mg, 0.62 mmol) and the crude product was purified by flash column chromatography and SFC to yield **2** (40 mg, 10%). ^1^H NMR (400 MHz, *d*-DMSO) δ 11.49 (d, *J* = 2.3 Hz, 1H), 8.69 (dd, *J* = 34.4, 7.8 Hz, 2H), 7.63 (s, 1H), 7.35–7.26 (m, 2H), 7.24–7.16 (m, 2H), 7.09 (t, *J* = 8.0 Hz, 1H), 7.02–6.93 (m, 2H), 6.54–6.46 (m, 1H), 5.44 (t, *J* = 5.8 Hz, 1H), 5.27 (q, *J* = 18.0 Hz, 2H), 4.72 (q, *J* = 7.8 Hz, 1H), 4.47 (d, *J* = 5.8 Hz, 2H), 4.44–4.35 (m, 1H), 3.89 (s, 3H), 3.19–3.00 (m, 4H), 2.06–1.89 (m, 3H), 1.65–1.50 (m, 2H), 1.27–1.20 (m, 1H). ^13^C NMR (126 MHz, *d*-DMSO) δ 203.8, 178.1, 172.0, 162.0 (d, *J* = 242.5 Hz), 161.2, 153.6, 145.8 (m), 143.8 (m), 141.1 (d, *J* = 7.5 Hz), 140.6 (d, *J* = 15.4 Hz), 138.6 (d, *J* = 16.3 Hz), 137.8, 135.8 (m), 130.0 (d, *J* = 8.4 Hz), 129.6, 125.4 (d, *J* = 2.6 Hz), 124.5, 118.0, 116.0 (d, *J* = 21.1 Hz), 113.2 (d, *J* = 20.7), 112.8 (m), 105.5, 101.2, 99.2, 74.9, 55.1, 54.6, 53.9, 50.6, 37.1, 36.7, 30.8, 27.1. ^19^F NMR (377 MHz, *d*-DMSO) δ −115.61 (m), −148.13 (dd, *J* = 23.7, 8.7 Hz), −159.43 (dd, *J* = 23.6, 8.6 Hz). HRMS (*m*/*z*): [M + H]^+^ calcd for C_34_H_31_F_5_N_4_O_7_: 703.2186; found: 703.2192.

##### (*S*)-3-(3-Fluorophenyl)-2-octanamidopropanoic Acid (**S6**)

Following GP4, using 2-chloro CTC resin (5.0 g, 1.2 mmol/g), Fmoc-3-fluoro-l-phenylalanine (3.0 equiv) and octanoic acid (3.0 equiv) were coupled. The dipeptide was cleaved off the resin using 5% TFA in DCM as cleavage cocktail, yielding **S6** (1.0 g, 56%), which was used directly without further purification. ^1^H NMR (400 MHz, CDCl_3_) δ 8.61 (bs, 1H), 7.28 – 7.18 (m, 1H), 7.00–6.81 (m, 3H), 6.22 (d, *J* = 7.5 Hz, 1H), 4.87 (dt, *J* = 7.5, 5.9 Hz, 1H), 3.17 (ddd, *J* = 53.6, 14.0, 5.9 Hz, 2H), 2.20 (td, *J* = 7.4, 2.4 Hz, 2H), 1.56 (p, *J* = 7.1 Hz, 2H), 1.33–1.16 (m, 8H), 0.90–0.83 (m, 3H). ^19^F NMR (377 MHz, CDCl_3_) δ −112.90. HRMS (*m*/*z*): [M + H]^+^ calcd for C_17_H_24_FNO_3_: 310.1805; found: 310.1813.

##### *N*-((*S*)-3-(3-Fluorophenyl)-1-oxo-1-(((*S*)-3-oxo-1-((*S*)-2-oxopyrrolidin-3-yl)-4-(2,3,5,6-tetrafluoro-4-(hydroxymethyl)­phenoxy)­butan-2-yl)­amino)­propan-2-yl)­octanamide (**3**)

*tert*-Butyl ((*S*)-3-oxo-1-((*S*)-2-oxopyrrolidin-3-yl)-4-(2,3,5,6-tetrafluoro-4-(hydroxymethyl)­phenoxy)­butan-2-yl)­carbamate (0.7 g, 1.51 mmol, 1.1 equiv) was deprotected following GP2. Subsequently, according to GP3 starting from (*S*)-3-(3-fluorophenyl)-2-octanamidopropanoic acid (452 mg, 1.46 mmol) and (*S*)-3-((*S*)-2-amino-3-oxo-4-(2,3,5,6-tetrafluoro-4-(hydroxymethyl)­phenoxy)­butyl)­pyrrolidin-2-one (TFA salt) (0.7 g, 1.463 mmol) the crude product was purified by RP-HPLC and SFC to yield **3** (80 mg, 8%). ^1^H NMR (500 MHz, *d*-DMSO) δ 8.60 (d, *J* = 8.0 Hz, 1H), 8.18 (d, *J* = 8.0 Hz, 1H), 7.67 (s, 1H), 7.27 (m, 1H), 7.10 (m, 2H), 6.96 (td, *J* = 8.7, 2.6 Hz, 1H), 5.51 (t, *J* = 5.8 Hz, 1H), 5.02 (ABq, *J* = 59.8 Hz, 2H), 4.50 (m, 3H), 4.39 (ddd, *J* = 11.7, 7.9, 3.9 Hz, 1H), 3.14 (t, *J* = 9.1 Hz, 1H), 3.07 (td, *J* = 9.2, 7.1 Hz, 1H), 2.99 (dd, *J* = 13.7, 5.3 Hz, 1H), 2.80 (dd, *J* = 13.7, 9.8 Hz, 1H), 2.19 (m, 1H), 2.04 (m, 3H), 1.94 (m, 1H), 1.60 (m, 2H), 1.35 (p, *J* = 7.4 Hz, 2H), 1.22 (m, 3H), 1.16 (m, 4H), 1.07 (m, 2H), 0.84 (t, *J* = 7.1 Hz, 3H). ^13^C NMR (126 MHz, *d*-DMSO) δ 203.3, 178.2, 172.4, 172.0, 162.0 (d, *J* = 243.0 Hz), 145.8 (m), 143.9 (m), 140.8 (d, *J* = 7.6 Hz), 140.6 (m), 138.7 (d, *J* = 16.1 Hz), 135.7 (m), 129.9 (d, *J* = 8.3 Hz), 125.3 (d, *J* = 2.7 Hz), 115.9 (d, *J* = 21.1 Hz), 113.1 (d, *J* = 20.9 Hz), 112.8 (d, *J* = 18.9 Hz), 74.9, 53.9, 53.7, 50.6, 37.1, 36.8, 35.1, 31.2, 30.6, 28.5, 28.4, 27.1, 25.2, 22.1, 14.0. ^19^F NMR (377 MHz, *d*-DMSO) δ −113.99 (q, *J* = 9.4 Hz), −146.34 (dd, *J* = 23.0, 8.9 Hz), −157.56 (dd, *J* = 23.3, 8.9 Hz). HRMS (*m*/*z*): [M + H]^+^ calcd for C_32_H_38_F_5_N_3_O_6_: 656.2754; found: 656.2710.

##### (*S*)-3-(3-Fluorophenyl)-2-palmitamidopropanoic Acid (**S7**)

Following GP4, using 2-chloro CTC resin (5.0 g, 1.2 mmol/g), Fmoc-3-fluoro-l-phenylalanine (3.0 equiv) was coupled and subsequently deprotected. Palmitic acid (3.0 equiv), PyBOP (3.0 equiv), DIPEA (5.0 equiv), DMF (10 vol) was added to the resin and gently agitated under nitrogen bubbling for 2 h. After cleavage, **S7** was obtained as an off-white solid (2.0 g, 80%), which was used directly without further purification. ^1^H NMR (CDCl_3_, 400 MHz) δ 9.09 (bs, 1H), 7.26 (td, *J* = 7.9, 5.9 Hz, 1H), 6.95 (m, 2H), 6.89 (dt, *J* = 9.6, 2.1 Hz, 1H), 6.45 (d, *J* = 7.6 Hz, 1H), 4.89 (dt, *J* = 7.7, 5.9 Hz, 1H), 3.19 (ddd, *J* = 57.5, 14.0, 5.9 Hz, 2H), 2.22 (td, *J* = 7.4, 2.5 Hz, 2H), 1.57 (p, *J* = 7.1 Hz, 2H), 1.28 (m, 24H), 0.90 (t, *J* = 6.8 Hz, 3H). ^13^C NMR (101 MHz, CDCl_3_) δ 175.0, 174.3, 162.9 (d, *J* = 246.5 Hz), 138.4 (d, *J* = 7.3 Hz), 130.1 (d, *J* = 8.2 Hz), 125.2 (d, *J* = 2.8 Hz), 116.4 (d, *J* = 21.3 Hz), 114.2 (d, *J* = 21.1 Hz), 53.2, 37.1, 36.5, 32.0, 29.8, 29.8, 29.8, 29.6, 29.5, 29.4, 29.3, 25.8, 22.8, 14.2. ESI-MS (*m*/*z*): [M + H]^+^ calcd for C_25_H_40_FNO_3_: 422.31; found: 422.44.

##### *N*-((*S*)-3-(3-Fluorophenyl)-1-oxo-1-(((*S*)-3-oxo-1-((*S*)-2-oxopyrrolidin-3-yl)-4-(2,3,5,6-tetrafluoro-4-(hydroxymethyl)­phenoxy)­butan-2-yl)­amino)­propan-2-yl)­palmitamide (**4**)

*tert*-Butyl ((*S*)-3-oxo-1-((*S*)-2-oxopyrrolidin-3-yl)-4-(2,3,5,6-tetrafluoro-4-(hydroxymethyl)­phenoxy)­butan-2-yl)­carbamate (364 mg, 0.78 mmol, 1.1 equiv) was deprotected following GP2. Subsequently, according to GP3 starting from (*S*)-3-(3-fluorophenyl)-2-palmitamidopropanoic acid (300 mg, 0.711 mmol) and (*S*)-3-((*S*)-2-amino-3-oxo-4-(2,3,5,6-tetrafluoro-4-(hydroxymethyl)­phenoxy)­butyl)­pyrrolidin-2-one (TFA salt) (374 mg, 0.78 mmol) the crude product was purified by flash column chromatography and SFC to yield **4** (74 mg, 14%). ^1^H NMR (400 MHz, *d*-DMSO) δ 8.57 (d, *J* = 7.8 Hz, 1H), 8.14 (d, *J* = 8.0 Hz, 1H), 7.63 (bs, 1H), 7.27 (td, *J* = 8.0, 6.2 Hz, 1H), 7.13–7.06 (m, 2H), 7.00–6.91 (m, 1H), 5.48 (t, *J* = 5.8 Hz, 1H), 5.03 (ABq, *J* = 48.6 Hz, 2H), 4.55–4.46 (m, 3H), 4.39 (ddd, *J* = 11.7, 8.0, 4.0 Hz, 1H), 3.19–3.02 (m, 2H), 3.00 (dd, *J* = 13.8, 5.4 Hz, 1H), 2.80 (dd, *J* = 13.7, 9.7 Hz, 1H), 2.25–2.14 (m, 1H), 2.12–2.04 (m, 1H), 2.03 (t, *J* = 7.3 Hz, 2H), 1.99–1.89 (m, 1H), 1.66–1.56 (m, 2H), 1.36 (p, *J* = 7.3 Hz, 2H), 1.29–1.06 (m, 24H), 0.89–0.81 (m, 3H). ^13^C NMR (126 MHz, *d*-DMSO) δ 203.2, 178.2, 172.4, 171.9, 162.0 (d, *J* = 243.0 Hz), 145.8 (m), 143.8 (m), 140.7 (d, *J* = 7.6 Hz), 140.5 (m), 138.7 (d, *J* = 15.8 Hz), 135.7 (m), 129.8 (d, *J* = 8.4 Hz), 125.3 (d, *J* = 3.1 Hz), 115.9 (d, *J* = 21.0 Hz), 113.0 (d, *J* = 20.9 Hz), 112.9 (m), 74.9, 53.8, 53.7, 50.6, 37.1, 36.8, 35.1, 31.3, 30.5, 29.0, 29.0, 29.0, 28.9, 28.8, 28.7, 28.4, 27.1, 25.2, 22.1, 13.9. ^19^F NMR (377 MHz, *d*-DMSO) δ −113.98 (m), −146.35 (dd, *J* = 23.5, 8.9 Hz), −157.56 (dd, *J* = 23.4, 8.9 Hz). HRMS (*m*/*z*): [M + H]^+^ calcd for C_40_H_54_F_5_N_3_O_6_: 768.4006; found: 768.3990.

##### (1*H*-Indole-2-carbonyl)-l-tryptophan (**S8**)

Following GP4, using 2-chloro CTC resin (10.0 g, 1.2 mmol/g), Fmoc-Trp­(Boc)–OH (3.0 equiv) and 1*H*-indole-2-carboxylic acid (2.0 equiv) were coupled. The dipeptide was cleaved off the resin using 10% TFA in DCM as cleavage cocktail, yielding **S8** as an off-white solid (4.0 g, 100%), which was used directly without further purification. ^1^H NMR (500 MHz, *d*-DMSO) δ 12.30–11.52 (m, 1H), 10.78 (s, 1H), 9.23–8.21 (m, 1H), 7.62 (d, *J* = 7.8 Hz, 1H), 7.59 (d, *J* = 8.0 Hz, 1H), 7.43 (d, *J* = 8.2 Hz, 1H), 7.30 (d, *J* = 7.6 Hz, 1H), 7.23 (d, *J* = 18.8 Hz, 1H), 7.16 (t, *J* = 7.6 Hz, 1H), 7.10 (s, 1H), 7.06–6.99 (m, 2H), 6.95 (t, *J* = 7.4 Hz, 1H), 4.68 (s, 1H), 3.40 (d, *J* = 14.3 Hz, 1H), 3.24 (dd, *J* = 14.5, 9.2 Hz, 1H). HRMS (*m*/*z*): [M + Na]^+^ calcd for C_20_H_17_N_3_O_3_: 370.1162; found: 370.1172.

##### *N*-((*S*)-3-(1*H*-Indol-3-yl)-1-oxo-1-(((*S*)-3-oxo-1-((*S*)-2-oxopyrrolidin-3-yl)-4-(2,3,5,6-tetrafluoro-4-(hydroxymethyl)­phenoxy)­butan-2-yl)­amino)­propan-2-yl)-1*H*-indole-2-carboxamide (**5**)

*tert*-Butyl ((*S*)-3-oxo-1-((*S*)-2-oxopyrrolidin-3-yl)-4-(2,3,5,6-tetrafluoro-4-(hydroxymethyl)­phenoxy)­butan-2-yl)­carbamate (1.8 g, 3.88 mmol) was deprotected following GP2. Subsequently, GP3 was followed using (1*H*-indole-2-carbonyl)-*L*-tryptophan (700 mg, 2.02 mmol) and (*S*)-3-((*S*)-2-amino-3-oxo-4-(2,3,5,6-tetrafluoro-4-(hydroxymethyl)­phenoxy)­butyl)­pyrrolidin-2-one (TFA salt) (1.06 g, 2.22 mmol) and the crude product was purified by flash column chromatography and RP-HPLC to yield **5** (24 mg, 2%). ^1^H NMR (400 MHz, *d*-DMSO) δ 11.53 (d, *J* = 2.2 Hz, 1H), 10.81 (d, *J* = 2.5 Hz, 1H), 8.74 (d, *J* = 8.1 Hz, 1H), 8.59 (d, *J* = 7.7 Hz, 1H), 7.71 (d, *J* = 7.6 Hz, 1H), 7.64–7.57 (m, 2H), 7.39 (d, *J* = 8.2 Hz, 1H), 7.32–7.19 (m, 3H), 7.16 (t, *J* = 7.6 Hz, 1H), 7.10–6.94 (m, 4H), 5.05 (ABq, *J* = 62.4 Hz, 2H), 4.80–4.67 (m, 1H), 4.49 (s, 2H), 4.48–4.43 (m, 1H), 3.31–3.23 (m, 1H), 3.20–2.98 (m, 3H), 2.34–2.23 (m, 1H), 2.10–1.94 (m, 2H), 1.69–1.57 (m, 2H). ^13^C NMR (126 MHz, *d*-DMSO) δ 203.5, 178.3, 172.5, 145.8 (m), 143.8 (m), 140.5 (m), 138.5 (d, *J* = 16.3 Hz), 136.4, 136.0, 135.7 (m), 131.2, 127.1, 127.0, 124.0, 123.4, 121.5, 120.9, 119.7, 118.4, 118.2, 112.7 (m), 112.3, 111.3, 110.1, 103.4, 74.7, 54.2, 53.7, 50.6, 37.1, 30.6, 27.1. ^19^F NMR (377 MHz, *d*-DMSO) δ −147.8 (dd, *J* = 23.5, 8.8 Hz), −159.13 (dd, *J* = 23.2, 8.6 Hz). HRMS (*m*/*z*): [M + H]^+^ calcd for C_35_H_31_F_4_N_5_O_6_: 694.2283; found: 694.2298.

##### *tert*-Butyl ((*S*)-4-Chloro-1-((*R*)-2,5-dioxopyrrolidin-3-yl)-3-oxobutan-2-yl)­carbamate (**S9**)

A solution of *tert*-butyl ((*S*)-4-chloro-3-oxo-1-((*S*)-2-oxopyrrolidin-3-yl)­butan-2-yl)­carbamate (2.5 g, 8.22 mmol, 1.0 equiv) was dissolved in EtOAc/H_2_O (1:1, 200 mL, 80 vol) and cooled to 0 °C. To this, NaIO_4_ (26.2 g, 123 mmol, 15.0 equiv) and RuCl_3_ (1.7 g, 8.22 mmol, 1.0 equiv) were added. The resulting reaction mixture was stirred at 0–10 °C for 2 h. Progress of the reaction was monitored by TLC. Upon completion of the reaction, it was filtered through Celite and extracted with EtOAc (3 × 100 mL). The combined organic layer was washed with brine (100 mL), dried over Na_2_SO_4_ and concentrated under vacuum to give crude material as brown gum. The obtained crude was purified by flash chromatography, eluting with 30–40% EtOAc in pet-ether to give **S9** (1.6 g, 61%) as an off-white solid. R*_f_* = 0.6 (EtOAc/heptane 6:4). ^1^H NMR (400 MHz, CDCl_3_) δ 8.16 (bs, 1H), 5.38 (d, *J* = 8.5 Hz, 1H), 4.70 (q, *J* = 7.7 Hz, 1H), 4.31 (d, *J* = 3.7 Hz, 2H), 3.10–3.00 (m, 1H), 2.99–2.91 (m, 1H), 2.55 (dd, *J* = 18.0, 5.00 Hz, 1H), 2.20–2.06 (m, 2H), 1.45 (s, 9H). ESI-MS (*m*/*z*): [M + H]^+^ calcd for C_13_H_19_ClN_2_O_5_: 319.11; found: 219.13 (-Boc).

##### *N*-((*S*)-1-(((*S*)-4-Chloro-1-((*R*)-2,5-dioxopyrrolidin-3-yl)-3-oxobutan-2-yl)­amino)-3-(3-fluorophenyl)-1-oxopropan-2-yl)-4-methoxy-1*H*-indole-2-carboxamide (**S10**)

*tert*-Butyl ((*S*)-4-chloro-1-((*R*)-2,5-dioxopyrrolidin-3-yl)-3-oxobutan-2-yl)­carbamate (1.1 g, 3.45 mmol, 1.1 equiv) was deprotected following GP2. Subsequently, starting from (*R*)-3-((*S*)-2-amino-4-chloro-3-oxobutyl)­pyrrolidine-2,5-dione (TFA salt) (1.0 g, 4.56 mmol) and (*S*)-3-(3-fluorophenyl)-2-(4-methoxy-1*H*-indole-2-carboxamido)­propanoic acid (1.62 g, 4.56 mmol) GP3 was followed and the crude product was purified by flash column chromatography (8→10% MeOH in DCM) to yield **S10** (1.0 g, 64%) as an off-white solid. R*_f_* = 0.5 (MeOH/DCM 1:10). ^1^H NMR (400 MHz, *d*-DMSO) δ 11.53 (bs, 1H), 11.13 (bs, 1H), 8.67 (dd, *J* = 19.6, 8.1 Hz, 2H), 7.35–6.90 (m, 7H), 6.55–6.44 (m, 1H), 4.75–4.59 (m, 1H), 4.48 (s, 3H), 3.89 (s, 3H), 3.21–3.12 (m, 2H), 3.10–2.99 (m, 2H), 2.10–1.84 (m, 3H). ESI-MS (*m*/*z*): [M + H]^+^ calcd for C_27_H_26_ClFN_4_O_6_: 557.16; found: 557.32.

##### *N*-((*S*)-1-(((*S*)-1-((*R*)-2,5-Dioxopyrrolidin-3-yl)-3-oxo-4-(2,3,5,6-tetrafluoro-4-(hydroxymethyl)­phenoxy)­butan-2-yl)­amino)-3-(3-fluorophenyl)-1-oxopropan-2-yl)-4-methoxy-1*H*-indole-2-carboxamide (**6**)

Following GP1, 6 was obtained using *N*-((*S*)-1-(((*S*)-4-chloro-1-((*R*)-2,5-dioxopyrrolidin-3-yl)-3-oxobutan-2-yl)­amino)-3-(3-fluorophenyl)-1-oxopropan-2-yl)-4-methoxy-1*H*-indole-2-carboxamide (300 mg, 0.54 mmol) and 2,3,5,6,-tetrafluoro-(4-hydroxymethyl)­phenol (105 mg, 0.54 mmol). The crude product was purified by flash column chromatography and RP-HPLC to yield **6** (13 mg, 4%) as an off-white solid. ^1^H NMR (500 MHz, *d*-ACN) δ 9.94 (s, 1H), 8.92 (s, 1H), 7.37 (dd, *J* = 11.4, 8.0 Hz, 2H), 7.26 (tt, *J* = 8.0, 6.1 Hz, 1H), 7.20–7.01 (m, 5H), 6.91 (tt, *J* = 8.7, 2.7 Hz, 1H), 6.54 (d, *J* = 7.8 Hz, 1H), 4.97 (ABq, 2H), 4.77 (td, *J* = 8.6, 6.1 Hz, 1H), 4.58 (s, 2H), 4.53 (ddt, *J* = 11.0, 8.3, 3.9 Hz, 1H), 3.92 (s, 3H), 3.29 (dd, *J* = 13.9, 5.8 Hz, 1H), 3.10 (dd, *J* = 13.9, 8.9 Hz, 1H), 2.83 (ddt, *J* = 14.7, 9.8, 4.9 Hz, 1H), 2.67 (dd, *J* = 18.0, 9.1 Hz, 1H), 2.38 (dd, *J* = 18.0, 5.4 Hz, 1H), 2.08 (ddd, *J* = 14.9, 11.0, 4.3 Hz, 2H), 2.02–1.96 (m, 2H). ^13^C NMR (126 MHz, *d*-ACN) δ 203.0, 181.4, 177.7, 163.5 (d, *J* = 243.4 Hz), 154.9, 147.2 (m), 145.2 (m), 142.1 (d, *J* = 15.4 Hz), 141.3 (d, *J* = 7.5 Hz), 140.2 (d, *J* = 15.8 Hz), 138.8, 137.0 (m), 131.0 (d, *J* = 8.4 Hz), 130.0, 126.4, 126.2 (d, *J* = 3.1 Hz), 119.4, 116.9 (d, *J* = 21.6 Hz), 114.3 (d, *J* = 21.1), 105.9, 101.4, 100.4, 76.1 (t, *J* = 3.7 Hz), 55.8, 55.7, 54.7, 52.0, 38.6, 37.3, 36.0, 31.1. ^19^F (377 MHz, *d*-ACN): δ −115.1 (td, *J* = 9.7, 6.1 Hz), −147.8 (dd, *J* = 22.2, 8.9, 1.7 Hz), −158.8 (dd, *J* = 21.2, 8.9 Hz). HRMS (*m*/*z*): [M + H]^+^ calcd for C_34_H_29_F_5_N_4_O_8_: 717.1978; found: 717.1962.

##### (1*R*,2*S*,5*S*)-3-((*S*)-3,3-Dimethyl-2-(2,2,2-trifluoroacetamido)­butanoyl)-6,6-dimethyl-*N*-((*S*)-3-oxo-1-((*S*)-2-oxopyrrolidin-3-yl)-4-(2,3,5,6-tetrafluoro-4-(hydroxymethyl)­phenoxy)­butan-2-yl)-3-azabicyclo­[3.1.0]­hexane-2-carboxamide (**7**)

*tert*-Butyl ((*S*)-3-oxo-1-((*S*)-2-oxopyrrolidin-3-yl)-4-(2,3,5,6-tetrafluoro-4-(hydroxymethyl)­phenoxy)­butan-2-yl)­carbamate (3.0 g, 6.46 mmol) was deprotected following GP2. Subsequently, starting from (*S*)-3-((*S*)-2-amino-3-oxo-4-(2,3,5,6-tetrafluoro-4-(hydroxymethyl)­phenoxy)­butyl)­pyrrolidin-2-one (TFA salt) (1.3 g, 2.64 mmol) and (1*R*,2*S*,5*S*)-3-((*S*)-3,3-dimethyl-2-(2,2,2-trifluoroacetamido)­butanoyl)-6,6-dimethyl-3-azabicyclo­[3.1.0]­hexane-2-carboxylic acid (0.800 g, 2.20 mmol) GP3 was followed and the crude product was purified by flash column chromatography and RP-HPLC to yield **7** (300 mg, 20%). ^1^H NMR (400 MHz, *d*-DMSO) δ 9.41 (d, *J* = 8.4 Hz, 1H), 8.69 (d, *J* = 8.4 Hz, 1H), 7.60 (s, 1H), 5.47 (t, *J* = 5.8 Hz, 1H), 5.26 (bs, 2H), 4.54–4.47 (m, 3H), 4.42 (d, *J* = 8.4 Hz, 1H), 4.23 (s, 1H), 3.91 (dd, *J* = 10.4, 5.5 Hz, 1H), 3.69 (d, *J* = 10.4 Hz, 1H), 3.14 (t, *J* = 9.1 Hz, 1H), 3.04 (q, *J* = 9.2 Hz, 1H), 2.43–2.34 (m, 1H), 2.15–2.06 (m, 1H), 2.00–1.91 (m, 1H), 1.67–1.56 (m, 2H), 1.55 (dd, *J* = 7.7, 5.6 Hz, 1H), 1.35 (d, *J* = 7.6 Hz, 1H), 1.03 (s, 3H), 0.97 (s, 9H), 0.85 (s, 3H). ^13^C NMR (126 MHz, *d*-DMSO) δ 203.2, 178.3, 171.3, 167.4, 156.9 (q, *J* = 37.1 Hz), 145.8 (m), 143.8 (m), 140.5 (d, *J* = 16.1 Hz), 138.6 (d, *J* = 16.5 Hz), 135.8 (m), 115.8 (q, *J* = 288.0 Hz), 112.8 (t, *J* = 18.9 Hz), 75.0 (t, *J* = 3.6 Hz), 60.2, 58.1, 53.2, 50.6, 47.6, 37.0, 34.6, 30.8, 30.3, 27.3, 27.1, 26.2, 25.8, 18.7, 12.3. ^19^F (377 MHz, *d*-DMSO): δ −72.9 (s), −146.3 (dd, *J* = 23.2, 8.7 Hz), −157.7 (dd, *J* = 23.3, 8.7 Hz). HRMS (*m*/*z*): [M + H]^+^ calcd for C_31_H_37_F_7_N_4_O_7_: 711.2623; found: 711.2610.

##### Methyl 2,3,5,6-Tetrafluoro-4-hydroxybenzoate (**S11**)

In a 250 mL round-bottom flask, 2,3,5,6-tetrafluoro-4-hydroxybenzoic acid (10.0 g, 47.6 mmol) was dissolved in MeOH (50 mL, 5 vol) and cooled to 0 °C. To this, H_2_SO_4_ (5 mL, 0.5 vol) was added dropwise over 2 min. The reaction mixture was stirred at 70 °C for 4 h. Progress of the reaction was monitored by TLC. Upon completion, it was diluted with ice cold water (200 mL) and extracted with EtOAc (3 × 500 mL). The combined organic layer was washed with sat. NaHCO_3_ (200 mL) followed by brine (200 mL), dried over Na_2_SO_4_ and concentrated under vacuum to give crude material as brown oil (11 g). The crude material triturated with Et_2_O to give **S11** (9.0 g, 85%) as an off-white solid, which was used without further purification. R*_f_* = 0.5 (MeOH/DCM 5:95). ^1^H NMR (400 MHz, *d*-DMSO) δ 3.78 (s, 3H). ESI-MS (*m*/*z*): [M + H]^+^ calcd for C_8_H_4_F_4_O_3_: 225.02; found: 225.10.

##### Methyl 4-(Benzyloxy)-2,3,5,6-tetrafluorobenzoate (**S12**)

In a 250 mL round-bottom flask, methyl 2,3,5,6-tetrafluoro-4-hydroxybenzoate (9.0 g, 40.0 mmol, 1 equiv) was dissolved in DMF (90 mL, 5 vol) and cooled to 0 °C. To this, was added K_2_CO_3_ (11.1 g, 80.0 mmol, 2 equiv) and benzyl bromide (7.15 mL, 60.0 mmol, 1.5 equiv). The reaction mixture was stirred at 25 °C for 6 h. Progress of the reaction was monitored by TLC. Upon completion, it was diluted with ice cold water (200 mL) and extracted with EtOAc (3 × 500 mL). The combined organic layer was washed with ice cold water (2 × 200 mL) followed by brine (200 mL), dried over Na_2_SO_4_ and concentrated under vacuum to give crude material as brown oil (∼15 g). The crude was purified by silica flash chromatography (20→25% EtOAc in pet-ether) to give **S12** (10 g, 79%) as an off-white solid. R*_f_* = 0.7 (EtOAc/Pet-ether 6:4). ^1^H NMR (400 MHz, CDCl_3_) δ 7.45–7.34 (m, 5H), 5.34 (s, 2H), 3.94 (s, 3H). ESI-MS (*m*/*z*): [M + H]^+^ calcd for C_15_H_10_F_4_O_3_: 315.06; found: 315.18.

##### 4-Benzyloxy-1,2,5,6-tetrafluorobenzaldehyde (**S13**)

In a 250 mL round-bottom flask, methyl 2,3,5,6-tetrafluoro-4-hydroxybenzoate (10.0 g, 31.8 mmol, 1 equiv) was dissolved in THF (200 mL, 20 vol) and cooled to −78 °C. To this was added, LAH (2 M in THF) (24.0 mL, 47.7 mmol, 1.5 equiv) dropwise over 10 min. The reaction mixture was stirred at −78 °C for 30 min. Progress of the reaction was monitored by TLC. Upon completion, it was diluted with EtOAc (500 mL), quenched with sat. NH_4_Cl solution (200 mL), filtered through Celite and extracted with EtOAc (3 × 500 mL). The combined organic layer was washed with saturated brine solution (500 mL), dried over Na_2_SO_4_ and concentrated under vacuum to give crude material as brown gum (∼10 g). The obtained crude was purified by silica flash chromatography (20→25% EtOAc in pet-ether) to give **S13** (6.0 g, 66%), as well as 4-(benzyloxy)-2,3,5,6-tetrafluorophenyl)­methanol (2.0 g, 22%), as off-white solids. **S13**: R*_f_* = 0.6 (EtOAc/Pet-ether 3:7). ^1^H NMR (400 MHz, CDCl_3_) δ 10.20 (t, *J* = 1.18 Hz, 1H), 7.45–7.35 (m, 5H), 5.42 (s, 2H). ESI-MS (*m*/*z*): [M + H]^+^ calcd for C_14_H_8_F_4_O_2_: 285.05; found: 285.20.

##### 1-(4-(Benzyloxy)-2,3,5,6-tetrafluorophenyl)­ethan-1-ol (**S14**)

To a 250 mL round-bottom flask, was added MeMgBr (1 M in THF) (105 mL, 106 mmol, 5 equiv) and THF (30 mL, 5 vol). To this, 4-(benzyloxy)-2,3,5,6-tetrafluorobenzaldehyde (6.0 g, 21.1 mmol, 1 equiv) in THF (30 mL, 5 vol) was added dropwise over 10 min at 25 °C. The resulting reaction mixture was stirred at 25 °C for 2 h. Progress of the reaction was monitored by TLC. Upon completion, it was diluted with saturated NH_4_Cl (100 mL) and extracted with EtOAc (3 × 200 mL). The combined organic layer was washed with saturated brine solution (100 mL), dried over Na_2_SO_4_ and concentrated under vacuum to give crude material as brown gum. The obtained crude was purified by silica flash chromatography (30→40% EtOAc in pet-ether) to give **S14** (4.5 g, 71%) as an off-white solid. R*_f_* = 0.3 (EtOAc/Pet-ether 2:3). ^1^H NMR (400 MHz, CDCl_3_) δ 7.45–7.33 (m, 5H), 5.23 (s, 2H), 5.26–5.17 (m, 1H), 2.11 (dt, *J* = 7.87, 1.33 Hz, 1H), 1.63 (d, *J* = 6.75 Hz, 3H). ESI-MS (*m*/*z*): [M–OH]^+^ calcd for C_15_H_12_F_4_O_2_: 283.07; found: 283.14.

##### 2,3,5,6-Tetrafluoro-4-(1-hydroxyethyl)­phenol (**S15**)

In a 250 mL round-bottom flask, 1-(4-(benzyloxy)-2,3,5,6-tetrafluorophenyl)­ethan-1-ol (1.0 g, 3.33 mmol) was dissolved in MeOH (20 mL, 20 vol). The reaction mixture was flushed with H_2_ and 10% Pd–C (200 mg, 20%) was added. The reaction mixture was stirred at 25 °C under 1 atm H_2_ pressure for 2 h. Progress of the reaction was monitored by TLC. Upon completion, it was filtered through Celite and the filtrate was concentrated to vacuum to give crude material as yellow oil. The crude was purified by silica flash chromatography (30→40% EtOAc in pet-ether) to give **S15** (0.5 g, 71%) as an off-white solid. R*_f_* = 0.2 (EtOAc/Pet-ether 2:3). ^1^H NMR (400 MHz, CDCl_3_) δ 5.24 (q, *J* = 6.74 Hz, 1H), 1.65 (dt, *J* = 6.71, 0.79 Hz, 3H). ESI-MS (*m*/*z*): [M–OH]^+^ calcd for C_8_H_6_F_4_O_2_: 193.03; found: 193.09.

##### *tert*-Butyl ((2*S*)-3-Oxo-1-((*S*)-2-oxopyrrolidin-3-yl)-4-(2,3,5,6-tetrafluoro-4-(1-hydroxyethyl)­phenoxy)­butan-2-yl)­carbamate (**S16**)

According to GP1 starting from *tert*-butyl ((*S*)-4-chloro-3-oxo-1-((*S*)-2-oxopyrrolidin-3-yl)­butan-2-yl)­carbamate (4.0 g, 13.2 mmol) and 2,3,5,6-tetrafluoro-4-(1-hydroxyethyl)­phenol (3.3 g, 15.8 mmol), **S16** was obtained as an off-white solid (3.5 g, 56%). R*_f_* = 0.4 (EtOAc/Pet-ether 3:2). ^1^H NMR (400 MHz, CDCl_3_) δ 6.17 (d, *J* = 7.25 Hz, 1H), 5.85 (bs, 1H), 5.22 (t, *J* = 6.34 Hz, 1H), 5.07 (ABq, *J* = 36.7 Hz, 2H), 4.57–4.47 (m, 1H), 3.41–3.32 (m, 2H), 2.54–2.42 (m, 2H), 2.40–2.34 (m, 1H), 2.10–2.01 (m, 1H), 1.98–1.81 (m, 2H), 1.63 (d, *J* = 6.74 Hz, 3H), 1.44 (s, 9H). ESI-MS (*m*/*z*): calcd for C_21_H_26_F_4_N_2_O_6_: 479.18; found: 479.19.

##### *N*-((2*S*)-3-(3-Fluorophenyl)-1-oxo-1-(((2*S*)-3-oxo-1-((*S*)-2-oxopyrrolidin-3-yl)-4-(2,3,5,6-tetrafluoro-4-(1-hydroxyethyl)­phenoxy)­butan-2-yl)­amino)­propan-2-yl)-4-methoxy-1*H*-indole-2-carboxamide (**8**)

*tert*-Butyl ((2*S*)-3-oxo-1-((*S*)-2-oxopyrrolidin-3-yl)-4-(2,3,5,6-tetrafluoro-4-(1-hydroxyethyl)­phenoxy)­butan-2-yl)­carbamate (600 mg, 1.25 mmol) was deprotected according to GP2. Subsequently, GP3 was followed using (*S*)-3-(3-fluorophenyl)-2-(4-methoxy-1*H*-indole-2-carboxamido)­propanoic acid (450 mg, 1.26 mmol) and (3*S*)-3-((2*S*)-2-amino-3-oxo-4-(2,3,5,6-tetrafluoro-4-(1-hydroxyethyl)­phenoxy)­butyl)­pyrrolidin-2-one (TFA salt) (620 mg, 1.26 mmol) and the crude product was purified by flash column chromatography and RP-HPLC to yield **8** (155 mg, 17%) as off-white solid. ^1^H NMR (400 MHz, *d*-DMSO) δ 11.53 (d, *J* = 2.3 Hz, 1H), 8.67 (dd, *J* = 15.0, 8.1 Hz, 2H), 7.63 (s, 1H), 7.34–7.18 (m, 4H), 7.08 (t, *J* = 7.9 Hz, 1H), 7.00–6.92 (m, 2H), 6.50 (d, *J* = 7.7 Hz, 1H), 5.60 (d, *J* = 4.4 Hz, 1H), 5.21–5.01 (m, 3H), 4.75–4.67 (m, 1H), 4.48 (ddd, *J* = 11.7, 8.0, 3.9 Hz, 1H), 3.89 (s, 3H), 3.17–2.99 (m, 4H), 2.31–2.24 (m, 1H), 2.11–1.94 (m, 2H), 1.69–1.56 (m, 2H), 1.46 (d, *J* = 6.7 Hz, 3H). ^13^C NMR (126 MHz, *d*-DMSO) δ 203.3, 178.2, 171.9, 162.0 (d, *J* = 243 Hz), 161.1, 153.6, 145.2 (m), 143.3 (m), 141.1 (d, *J* = 7.5 Hz), 140.7 (d, *J* = 16.2 Hz), 138.8 (d, *J* = 16.2 Hz), 137.8, 135.0 (m), 129.9 (d, *J* = 8.2 Hz), 129.6, 125.3 (d, *J* = 2.6 Hz), 124.5, 118.0, 117.1 (t, *J* = 15.8 Hz), 115.9 (d, *J* = 21.1 Hz), 113.1 (d, *J* = 21.0 Hz), 105.4, 101.0, 99.2, 74.8, 59.9, 55.0, 54.5, 53.6, 37.1, 36.5, 30.6, 27.1, 22.5. ^19^F (377 MHz, *d*-DMSO): δ −113.85 (m), −145.82 (d, *J* = 22.9, 8.2 Hz), −157.66 (dd, *J* = 22.6, 8.4 Hz). HRMS (*m*/*z*): [M + H]^+^ calcd for C_35_H_33_F_5_N_4_O_7_: 717.2342; found: 717.2324.

##### *N*-((2*S*)-1-(((2*S*)-1-((*R*)-2,5-Dioxopyrrolidin-3-yl)-3-oxo-4-(2,3,5,6-tetrafluoro-4-(1-hydroxyethyl)­phenoxy)­butan-2-yl)­amino)-3-(3-fluorophenyl)-1-oxopropan-2-yl)-4-methoxy-1*H*-indole-2-carboxamide (**9**)

Following GP1, 9 was obtained using *N*-((*S*)-1-(((*S*)-4-chloro-1-((*R*)-2,5-dioxopyrrolidin-3-yl)-3-oxobutan-2-yl)­amino)-3-(3-fluorophenyl)-1-oxopropan-2-yl)-4-methoxy-1*H*-indole-2-carboxamide (300 mg, 0.54 mmol) and 2,3,5,6-tetrafluoro-4-(1-hydroxyethyl)­phenol (113 mg, 0.54 mmol). The crude product was purified by flash column chromatography, RP-HPLC and chiral SFC to yield **9** (36 mg, 4%) as an off-white solid. ^1^H NMR (400 MHz, *d*-ACN) δ 10.09 (bs, 1H), 9.04 (bs, 1H), 7.51 (dd, *J* = 23.2, 8.1 Hz, 2H), 7.37 (td, *J* = 8.0, 6.1 Hz, 1H), 7.29 (t, *J* = 8.0 Hz, 1H), 7.24 (d, *J* = 7.7 Hz, 1H), 7.22–7.13 (m, 3H), 7.03–6.98 (m, 1H), 6.65 (d, *J* = 7.8 Hz, 1H), 5.26 (q, *J* = 6.9 Hz, 1H), 5.07 (ABq, 2H), 4.88 (ddd, *J* = 9.2, 7.8, 5.9 Hz, 1H), 4.65 (ddd, *J* = 11.0, 8.4, 4.1 Hz, 1H), 4.03 (s, 3H), 3.75 (bs, 1H), 3.40 (dd, *J* = 14.0, 5.9 Hz, 1H), 3.22 (dd, *J* = 14.0, 9.2 Hz, 1H), 2.99–2.91 (m, 1H), 2.78 (dd, *J* = 18.1, 9.1 Hz, 1H), 2.49 (dd, *J* = 18.1, 5.5 Hz, 1H), 2.18 (ddd, *J* = 14.9, 11.0, 4.4 Hz, 1H), 2.13–2.06 (m, 1H), 1.62 (d, *J* = 6.7 Hz, 3H). ^13^C NMR (126 MHz, *d*-ACN) δ 203.2, 181.4, 177.8, 172.6, 163.6 (d, *J* = 243.4 Hz), 162.4, 155.0, 146.8 (m), 144.8 (m), 142.4 (d, *J* = 16.3 Hz), 141.4 (d, *J* = 7.6 Hz), 140.4 (d, *J* = 16.4 Hz), 138.9, 136.4, 131.1 (d, *J* = 8.4 Hz), 130.1, 126.5, 126.3 (d, *J* = 2.8 Hz), 119.5, 117.0 (d, *J* = 21.2 Hz), 114.3 (d, *J* = 21.1 Hz), 106.0, 101.5, 100.5, 76.1 (t, *J* = 3.7 Hz), 61.9, 55.9, 55.8, 54.7, 38.7, 37.4, 36.1, 31.2, 22.8. ^19^F (471 MHz, *d*-ACN): δ −115.1 (q, *J* = 9.6, 6.1 Hz), −147.3 (dd, *J* = 20.9, 8.4 Hz), −158.9– −159.0 (m). HRMS (*m*/*z*): [M + H]^+^ calcd for C_35_H_31_F_5_N_4_O_8_: 731.2135; found: 731.2112.

##### 1-(4-(Benzyloxy)-2,3,5,6-tetrafluorophenyl)­ethan-1-one (**S17**)

To a 250 mL round-bottom flask, was added PCC (7.1 g, 33.3 mmol, 5 equiv), 4 Å molecular sieves (7.10 g) and DCM (60 mL, 30 vol). The reaction mixture was cooled to 0 °C and a solution of 1-(4-(benzyloxy)-2,3,5,6-tetrafluorophenyl)­ethan-1-ol (1.0 equiv, 2.0 g, 6.66 mmol) in DCM (20 mL, 10 vol) dropwise over 10 min. The resulting reaction mixture was stirred at 25 °C for 2 h. Progress of the reaction was monitored by TLC. Upon completion, it was filtered through 230–400 mesh silica and the filtrate was concentrated to vacuum, to give the crude material as brown gum. The obtained crude was purified by silica flash chromatography (5→10% EtOAc in pet-ether) to give **S17** (1.5 g, 75%) as an off-white solid. R*_f_* = 0.6 (EtOAc/Pet-ether 2:3). ^1^H NMR (400 MHz, CDCl_3_) δ 7.45–7.34 (m, 5H), 5.34 (s, 2H), 2.58 (t, *J* = 2.1 Hz, 3H). ESI-MS (*m*/*z*): [M + H]^+^ calcd for C_15_H_10_F_4_O_2_: 299.07; found: 299.18.

##### 2-(4-(Benzyloxy)-2,3,5,6-tetrafluorophenyl)­propan-2-ol (**S18**)

To a 100 mL round-bottom flask, was added MeMgBr (1 M in THF) (25 mL, 25.2 mmol, 5 equiv) and THF (30 mL, 5 vol). To this, 1-(4-(benzyloxy)-2,3,5,6-tetrafluorophenyl)­ethan-1-one (1.5 g, 5.03 mmol, 1 equiv) in THF (7.5 mL, 5 vol) was added dropwise over 10 min at 25 °C. The resulting reaction mixture was stirred at 25 °C for 1 h. Progress of the reaction was monitored by TLC. Upon completion, it was diluted with saturated NH_4_Cl (50 mL) and extracted with EtOAc (3 × 100 mL). The combined organic layer was washed with sat. brine solution (50 mL), dried over Na_2_SO_4_ and concentrated under vacuum to give crude material as brown gum. The crude was purified by silica flash chromatography (30→40% EtOAc in pet-ether) to give **S18** (1.1 g, 70%) as an off-white solid. R*_f_* = 0.3 (EtOAc/Pet-ether 2:3). ^1^H NMR (400 MHz, CDCl_3_) δ 7.46–7.33 (m, 5H), 5.24 (s, 2H), 2.69 (t, *J* = 4.32 Hz, 1H), 1.71 (t, *J* = 2.08 Hz, 6H). ESI-MS (*m*/*z*): [M – OH]^+^ calcd for C_16_H_14_F_4_O_2_: 297.09; found: 297.23.

##### 2,3,5,6-Tetrafluoro-4-(2-hydroxypropan-2-yl)­phenol (**S19**)

In a 250 mL round-bottom flask, 2-(4-(benzyloxy)-2,3,5,6-tetrafluorophenyl)­propan-2-ol (1.2 g, 3.82 mmol) was dissolved in MeOH (20 mL, 20 vol). The reaction mixture was flushed with H_2_ and added 10% Pd–C (240 mg, 20%). The heterogeneous mass was stirred at 25 °C under 1 atm H_2_ pressure for 2 h. Progress of the reaction was monitored by TLC. Upon completion, it was filtered through Celite and the filtrate was concentrated to vacuum to give crude material as yellow oil. The obtained crude was purified by silica flash chromatography (30→40% EtOAc in pet-ether) to give **S19** (0.7 g, 82%) as an off-white solid. R*_f_* = 0.2 (EtOAc/Pet-ether 2:3). ^1^H NMR (400 MHz, CDCl_3_) δ 5.89 (bs, 1H), 2.72 (bs, 1H), 1.72 (t, *J* = 2.07 Hz, 6H), 1.67 (t, *J* = 2.39 Hz, 1H). ESI-MS (*m*/*z*): [M – OH]^+^ calcd for C_9_H_8_F_4_O_2_: 207.04; found: 207.14.

##### *N*-((*S*)-1-(((S)-4-Chloro-3-oxo-1-((*S*)-2-oxopyrrolidin-3-yl)­butan-2-yl)­amino)-3-(3-fluorophenyl)-1-oxopropan-2-yl)-4-methoxy-1*H*-indole-2-carboxamide (**S20**)

*tert*-Butyl ((*S*)-4-chloro-3-oxo-1-((*S*)-2-oxopyrrolidin-3-yl)­butan-2-yl)­carbamate (5.0 g, 16.4 mmol) was deprotected according to GP2. Subsequently, GP3 was followed using (*S*)-3-(3-fluorophenyl)-2-(4-methoxy-1*H*-indole-2-carboxamido)­propanoic acid (3.8 g, 10.7 mmol) and ((*S*)-3-((*S*)-2-amino-4-chloro-3-oxobutyl)­pyrrolidin-2-one (TFA salt) (5.12 g, 16.0 mmol). The crude material was purified by C18 reverse phase column chromatography (35→40% ACN in H_2_O) to give **S20** as an off-white solid (3.4 g, 34%). ^1^H NMR (400 MHz, *d*-DMSO) δ 11.54 (d, *J* = 2.38 Hz, 1H), 8.70 (d, *J* = 7.90 Hz, 1H), 8.63 (d, *J* = 8.01 Hz, 1H), 7.63 (s, 1H), 7.31 (m, 2H), 7.22 (m, 2H), 7.09 (m, 1H), 6.99 (m, 2H), 6.50 (d, *J* = 7.75 Hz, 1H), 4.71 (m, 1H), 4.50 (m, 2H), 4.46 (m, 1H), 3.89 (s, 3H), 3.11 (m, 4H), 2.29 (m, 1H), 2.02 (m, 2H), 1.64 (m, 2H). ESI-MS (*m*/*z*): [M + H]^+^ calcd for C_27_H_28_ClFN_4_O_5_: 543.18; found: 543.21.

##### *N*-((*S*)-3-(3-Fluorophenyl)-1-oxo-1-(((*S*)-3-oxo-1-((*S*)-2-oxopyrrolidin-3-yl)-4-(2,3,5,6-tetrafluoro-4-(2-hydroxypropan-2-yl)­phenoxy)­butan-2-yl)­amino)­propan-2-yl)-4-methoxy-1*H*-indole-2-carboxamide (**10**)

Following GP1, 10 was obtained using *N*-((*S*)-1-(((*S*)-4-chloro-3-oxo-1-((*S*)-2-oxopyrrolidin-3-yl)­butan-2-yl)­amino)-3-(3-fluorophenyl)-1-oxopropan-2-yl)-4-methoxy-1*H*-indole-2-carboxamide (3.4 g, 6.26 mmol) and 2,3,5,6-tetrafluoro-4-(2-hydroxypropan-2-yl)­phenol (1.4 g, 6.26 mmol). The crude product was purified by flash column chromatography and RP-HPLC to yield **10** (350 mg, 8%) as off-white solid. ^1^H NMR (400 MHz, *d*-DMSO) δ 11.54–11.49 (m, 1H), 8.75–8.62 (m, 2H), 7.63 (bs, 1H), 7.34–7.17 (m, 4H), 7.08 (t, *J* = 7.97 Hz, 1H), 7.03–6.91 (m, 2H), 6.50 (d, *J* = 7.71 Hz, 1H), 5.21–5.01 (ABq, 2H), 4.77–4.66 (m, 1H), 4.51–4.44 (m, 1H), 3.89 (s, 3H), 3.19–2.99 (m, 4H), 2.32–2.24 (m, 1H), 2.12–2.03 (m, 1H), 2.03–1.94 (m, 1H), 1.70–1.60 (m, 2H), 1.59–1.53 (m, 6H). ^13^C NMR (126 MHz, *d*-DMSO) δ 203.4, 178.2, 171.9, 162.0 (d, *J* = 243.0 Hz), 161.1, 153.6, 145.4 (m), 143.4 (m), 141.1 (d, *J* = 7.5 Hz), 140.9 (m), 139.1 (m), 137.8, 134.4 (m), 129.9 (d, *J* = 8.4 Hz), 129.6, 125.3 (d, *J* = 2.6 Hz), 124.5, 119.9 (m), 118.0, 115.9 (d, *J* = 21.1 Hz), 113.1 (d, *J* = 20.7 Hz), 105.4, 101.0, 99.2, 74.7, 71.4, 55.0, 54.5, 53.6, 37.1, 36.5, 30.9 (t, *J* = 3.7 Hz), 30.6, 27.1, ^19^F (377 MHz, *d*-DMSO): δ −113.85 (m), −141.1 (d, *J* = 22.5, 7.0 Hz), −158.0 (d, *J* = 22.2, 7.0 Hz). HRMS (*m*/*z*): [M + H]^+^ calcd for C_36_H_35_F_5_N_4_O_7_: 731.2499; found: 731.2474.

##### *N*-((*S*)-3-(3-Fluorophenyl)-1-oxo-1-(((*S*)-3-oxo-1-((*S*)-2-oxopyrrolidin-3-yl)-4-(2,3,5,6-tetrafluoro-4-(2-hydroxypropan-2-yl)­phenoxy)­butan-2-yl)­amino)­propan-2-yl)-4-methoxy-1*H*-indole-2-carboxamide (**11**)

Following GP1, 11 was obtained using *N*-((*S*)-1-(((*S*)-4-chloro-1-((*R*)-2,5-dioxopyrrolidin-3-yl)-3-oxobutan-2-yl)­amino)-3-(3-fluorophenyl)-1-oxopropan-2-yl)-4-methoxy-1*H*-indole-2-carboxamide (300 mg, 0.54 mmol) and 2,3,5,6-tetrafluoro-4-(2-hydroxypropan-2-yl)­phenol (120 mg, 0.54 mmol). The crude product was purified by flash column chromatography, RP-HPLC and chiral SFC to yield 11 (14 mg, 3%) as an off-white solid. ^1^H NMR (400 MHz, *d*-ACN) δ 9.88 (bs, 1H), 8.86 (bs, 1H), 7.33 (dd, *J* = 14.7, 8.1 Hz, 2H), 7.25 (td, *J* = 8.0, 6.1 Hz, 1H), 7.17 (t, *J* = 8.0 Hz, 1H), 7.12 (d, *J* = 7.7 Hz, 1H), 7.10–7.00 (m, 3H), 6.89 (td, *J* = 8.6, 2.7 Hz, 1H), 6.53 (d, *J* = 7.7 Hz, 1H), 4.95 (ABq, 2H), 4.78–4.71 (m, 1H), 4.56–4.49 (m, 1H), 3.91 (s, 3H), 3.63 (bs, 1H), 3.28 (dd, *J* = 14.0, 5.9 Hz, 1H), 3.09 (d, *J* = 14.0, 9.1 Hz, 1H), 2.83 (tt, *J* = 9.8, 4.9 Hz, 1H), 2.66 (dd, *J* = 18.1, 9.1 Hz, 1H), 2.37 (dd, *J* = 18.0, 5.5 Hz, 1H), 2.06 (ddd, *J* = 15.0, 11.0, 4.4 Hz, 1H), 2.00–1.95 (m, 1H), 1.61 (t, *J* = 2.0 Hz, 6H). ^13^C (126 MHz, *d*-ACN): δ 203.2, 181.4, 177.7, 172.5, 163.4 (d, *J* = 285.2 Hz), 162.6, 155.0, 146.8, 144.8, 142.7, 141.4 (d, *J* = 7.9 Hz), 140.6, 138.9, 131.1 (d, *J* = 8.4 Hz), 130.1, 126.4, 126.4, 126.3 (d, *J* = 2.6 Hz), 119.5, 116.9 (d, *J* = 21.4 Hz), 114.3 (d, *J* = 21.1 Hz), 106.0, 101.4, 100.5, 76.0 (t, *J* = 3.5 Hz), 55.9, 55.8, 54.7, 38.7, 37.4, 36.1, 31.2, 31.2, 31.1, 30.6. ^19^F (377 MHz, *d*-ACN): δ −116.0 (td, *J* = 9.6, 6.0 Hz), −143.7 (m), −160.2 (dd, *J* = 20.0, 7.2 Hz). HRMS (*m*/*z*): [M + H]^+^ calcd for C_36_H_33_F_5_N_4_O_8_: 745.2291; found: 745.2273.

##### 4-(4-Methoxybenzyl)-2,4-dihydro-3*H*-1,2,4-triazol-3-one (**S21**)

In a round-bottom flask, 2,4-dihydro-3*H*-1,2,4-triazol-3-one (2.5 g, 29.4 mmol) was dissolved in DMF (50 mL, 20 vol) and cooled to 0 °C. To this, was added K_2_CO_3_ (6.08 g, 44.1 mmol) followed by PMB-Cl (1.99 mL, 14.7 mmol). The reaction mixture was stirred at 25 °C for 4 h. Progress of the reaction was monitored by TLC. Upon completion, it was diluted with ice cold water (120 mL) and extracted with EtOAc (3 × 100 mL). The combined organic layer was washed with ice cold water (3 × 50 mL) followed by brine (100 mL) and dried over Na_2_SO_4_. It was concentrated under vacuum to give crude material as brown oil (∼2.8 g). The crude was purified by silica flash chromatography (100% EtOAc in pet-ether) to give **S21** (1.0 g, 17%) as an off-white solid. R_f_ = 0.4 (EtOAc/Pet-ether 3:2). ^1^H NMR (400 MHz, *d*-DMSO) δ 11.65 (bs, 1H), 7.91 (d, *J* = 1.41 Hz, 1H), 7.24 (d, *J* = 8.69 Hz, 2H), 6.91 (d, *J* = 8.70 Hz, 2H), 4.66 (s, 2H), 3.73 (s, 3H). ESI-MS (*m*/*z*): [M + H]^+^ calcd for C_10_H_11_N_3_O_2_: 206.09; found: 206.07.

##### *tert*-Butyl (*S*)-2-((*tert*-Butoxycarbonyl)­amino)-4-(4-(4-methoxybenzyl)-5-oxo-4,5-dihydro-1*H*-1,2,4-triazol-1-yl)­butanoate (**S22**)

In a round-bottom flask, 4-(4-methoxybenzyl)-2,4-dihydro-3*H*-1,2,4-triazol-3-one (1.0 g, 4.87 mmol) was dissolved in DMF (10 mL, 10 vol) and cooled to 0 °C. To this, was added Cs_2_CO_3_ (2.38 g, 7.30 mmol) and stirred for 5 min followed by the dropwise addition of *tert*-butyl (*S*)-2-((*tert*-butoxycarbonyl)­amino)-4-iodobutanoate (1.88 g, 4.87 mmol) in DMF (10 mL). The reaction mixture was stirred at 25 °C for 4 h. Progress of the reaction was monitored by TLC. Upon completion, it was diluted with ice cold water (120 mL) and extracted with EtOAc (3 × 50 mL). The combined organic layer was washed with ice cold water (3 × 30 mL) followed by brine (60 mL), dried over Na_2_SO_4_ and concentrated under vacuum to give crude material as brown oil (∼1.4 g). The crude was purified by silica flash chromatography (30% EtOAc in pet-ether) to give **S22** (2.0 g, 88%) as an off-white solid. R_f_ = 0.4 (EtOAc/pet-ether 2:3). ^1^H NMR (400 MHz, CDCl_3_) δ 7.25–7.21 (m, 3H), 6.92–6.86 (m, 2H), 5.31 (d, *J* = 8.6 Hz, 1H), 4.70 (s, 2H), 4.27 (d, *J* = 7.0 Hz, 1H), 3.89 (t, *J* = 7.4 Hz, 2H), 3.80 (s, 3H), 2.29–2.20 (m, 1H), 2.13–2.01 (m, 1H), 1.44 (s, 18H). ESI-MS (*m*/*z*): [M + H]^+^ calcd for C_23_H_34_N_4_O_6_: 463.26; found: 463.27.

##### (*S*)-2-((*tert*-Butoxycarbonyl)­amino)-4-(4-(4-methoxybenzyl)-5-oxo-4,5-dihydro-1*H*-1,2,4-triazol-1-yl)­butanoic Acid (**S23**)

According to GP2, *tert*-butyl (*S*)-2-((tert-butoxycarbonyl)­amino)-4-(4-(4-methoxybenzyl)-5-oxo-4,5-dihydro-1*H*-1,2,4-triazol-1-yl)­butanoate (2.0 g, 4.32 mmol) was deprotected and the crude reaction mixture was directly used without further purification. ESI-MS (*m*/*z*): [M + H]^+^ calcd for C_14_H_18_N_4_O_4_: 307.14; found: 307.19. A solution of ((*S*)-2-amino-4-(4-(4-methoxybenzyl)-5-oxo-4,5-dihydro-1*H*-1,2,4-triazol-1-yl)­butanoic acid (2.0 g, 6.52 mmol) in dioxane:H_2_O (1:1) (40 mL, 20 vol) was cooled to 0 °C and NaHCO_3_ was added (2.19 g, 26.11 mmol), followed by (Boc)_2_O (4.49 mL, 19.6 mmol). The contents were stirred for 2 h at 25 °C. Progress of the reaction was monitored by TLC. After completion of reaction, it was diluted with water (40 mL, 20 vol) and extracted with EtOAc (3 × 60 mL). The combined organic layer was washed with brine solution (100 mL) and dried over Na_2_SO_4_ and concentrated under vacuum to give **S23** (2.0 g, 75%) as an off-white solid. The crude reaction mixture was directly used without further purification. ESI-MS (*m*/*z*): [M + H]^+^ calcd for C_19_H_26_N_4_O_6_: 407.19; found: 407.24.

##### *tert*-Butyl (*S*)-(1-Chloro-5-(4-(4-methoxybenzyl)-5-oxo-4,5-dihydro-1H-1,2,4-triazol-1-yl)-2-oxopentan-3-yl)­carbamate (**S24**)

A solution of (*S*)-2-((*tert*-butoxycarbonyl)­amino)-4-(4-(4-methoxybenzyl)-5-oxo-4,5-dihydro-1*H*-1,2,4-triazol-1-yl)­butanoic acid (2.0 g, 4.92 mmol) in THF (40 mL, 20 vol) was cooled to −10 °C and added triethylamine (0.89 mL, 6.39 mmol), followed by isobutyl chloroformate (0.76 mL, 5.90 mmol). The resulting reaction mixture was stirred at −10 °C for 30 min. After completion of reaction, the heterogeneous mixture was filtered and washed with THF (10 mL). The filtrate was taken in RBF and was cooled to −15 °C. To this, was added freshly prepared diazomethane in diethyl ether (20 mL) dropwise at −10 °C. The resulting reaction mixture was stirred for 30 min at −10 °C. Progress of the reaction was monitored by TLC. After completion of reaction, it was quenched with acetic acid until colorless and diluted with water (80 mL, 40 vol) and extracted with EtOAc (3 × 100 mL). The combined organic layer was washed with brine solution (100 mL) and dried over Na_2_SO_4_. The volatiles were removed under vacuum and the resulting yellow semisolid (2.0 g, crude) was used without further purification. R_f_ = 0.5 (EtOAc/Pet-ether 3:2). ESI-MS (*m*/*z*): [M + H]^+^ calcd for C_20_H_26_N_6_O_5_: 431.20; found: 403.70 (-N_2_). The obtained semisolid intermediate (2.0 g, 4.65 mmol) was dissolved in THF (40 mL, 20 vol) and the solution was cooled to −10 °C and 4 M HCl in dioxane (4.65 mL, 18.6 mmol) was added. The reaction mixture was stirred for 30 min at the same temperature. Progress of the reaction was monitored by TLC. After completion of reaction, it was concentrated and triturated with pentane (2 × 10 mL) to give **S24** (1.8 g, crude) as a light yellow gum. ^1^H NMR (400 MHz, CDCl_3_) δ 7.25–7.20 (m, 3H), 6.90 (d, *J* = 8.66 Hz, 2H), 5.83 (d, *J* = 8.50, 1H), 4.69 (s, 2H), 4.46 (q, *J* = 6.65, 1H), 4.37–4.25 (m, 2H), 4.01–3.83 (m, 2H), 3.80 (s, 3H), 2.24 (h, *J* = 6.74 Hz, 2H), 1.45 (s, 9H). ESI-MS (*m*/*z*): [M + H]^+^ calcd for C_20_H_27_ClN_4_O_5_: 439.17; found: 439.58.

##### (*S*)-2-(3-Amino-5-chloro-4-oxopentyl)-2,4-dihydro-3*H*-1,2,4-triazol-3-one (**S25**)

To a solution of (*tert*-butyl (*S*)-(1-chloro-5-(4-(4-methoxybenzyl)-5-oxo-4,5-dihydro-1*H*-1,2,4-triazol-1-yl)-2-oxopentan-3-yl)­carbamate (400 mg, 0.91 mmol) in anisole (8 mL, 20 vol) at 25 °C was added TfOH (0.32 mL, 3.65 mmol). The contents were irradiated in microwave at 100 °C for 20 min. Progress of the reaction was monitored by TLC. After completion of reaction, it was concentrated under reduced pressure. The crude was diluted with water (8 mL, 20 vol) and extracted with EtOAc (3 × 15 mL). The combined organic layer was washed with water (3 × 10 mL), brine solution (10 mL) and dried over Na_2_SO_4_ and concentrated under vacuum to give **S25** (190 mg, crude) as a pale brown solid. This free amine was immediately coupled with the dipeptide. ESI-MS (*m*/*z*): [M + H]^+^ calcd for C_7_H_11_ClN_4_O_2_: 219.06; found: 219.36.

##### *N*-((*S*)-1-(((*S*)-1-Chloro-2-oxo-5-(5-oxo-4,5-dihydro-1*H*-1,2,4-triazol-1-yl)­pentan-3-yl)­amino)-3-(3-fluorophenyl)-1-oxopropan-2-yl)-4-methoxy-1*H*-indole-2-carboxamide (**S26**)

To a cooled solution (−5 °C) of (*S*)-3-(3-fluorophenyl)-2-(4-methoxy-1*H*-indole-2-carboxamido)­propanoic acid (0.6 g, 1.68 mmol) in DMF (18 mL, 30 vol), was added (*S*)-4-(3-amino-5-chloro-4-oxopentyl)-2,4-dihydro-3*H*-1,2,4-triazol-3-one (0.55 g, 2.52 mmol). The contents were stirred for 5 min at the same temperature followed by the addition of HATU (0.96 g, 2.52 mmol) and DIPEA (0.96 mL, 5.05 mmol). The resulting reaction mixture was stirred at −5 °C for 30 min. Progress of the reaction was monitored by TLC. After completion of reaction, it was diluted with water (24 mL, 40 vol) and extracted with EtOAc (3 × 50 mL). The combined organic layer was washed with ice cold water (3 × 30 mL), brine solution (50 mL) and dried over Na_2_SO_4_. The volatiles were removed under vacuum and the crude was purified by silica flash chromatography (2→3% MeOH in DCM) to give **S26** (33 mg, 4%) as an off-white solid. ^1^H NMR (400 MHz, *d*-DMSO) δ 11.54 (d, *J* = 2.3 Hz, 1H), 8.75 (d, *J* = 7.7 Hz, 1H), 8.66 (d, *J* = 7.8 Hz, 1H), 7.82 (s, 1H), 7.34–7.26 (m, 2H), 7.25–7.18 (m, 2H), 7.08 (t, *J* = 8.0 Hz, 1H), 6.97 (d, *J* = 8.3 Hz, 2H), 6.50 (d, *J* = 7.7 Hz, 1H), 4.75–4.65 (m, 1H), 4.48 (d, *J* = 1.4 Hz, 1H), 4.46–4.36 (m, 1H), 3.88 (s, 3H), 3.66 (t, *J* = 7.2 Hz, 2H), 3.18 (dd, *J* = 13.8, 4.5 Hz, 1H), 3.08–2.98 (m, 1H), 2.24–2.14 (m, 1H), 1.95–1.85 (m, 1H). ^19^F NMR (377 MHz, *d*-DMSO) δ −113.8 (m). HRMS (*m*/*z*): [M + H]^+^ calcd for C_26_H_26_ClFN_6_O_5_: 557.1710; found: 557.1711.

##### *N*-((*S*)-3-(3-Fluorophenyl)-1-oxo-1-(((*S*)-2-oxo-5-(5-oxo-1,5-dihydro-4*H*-1,2,4-triazol-4-yl)-1-(2,3,5,6-tetrafluoro-4-(2-hydroxypropan-2-yl)­phenoxy)­pentan-3-yl)­amino)­propan-2-yl)-4-methoxy-1*H*-indole-2-carboxamide (**12**)

Following GP1, 12 was obtained using *N*-((*S*)-1-(((*S*)-1-chloro-2-oxo-5-(5-oxo-4,5-dihydro-1*H*-1,2,4-triazol-1-yl)­pentan-3-yl)­amino)-3-(3-fluorophenyl)-1-oxopropan-2-yl)-4-methoxy-1*H*-indole-2-carboxamide (450 mg, 0.81 mmol) and 2,3,5,6-tetrafluoro-4-(2-hydroxypropan-2-yl)­phenol (109 mg, 0.49 mmol). The crude product was triturated with diethyl ether, purified by RP-HPLC and chiral SFC to yield **12** (42 mg, 12%) as off-white solid. R_f_ = 0.6 (EtOAc/pet-ether 3:2). ^1^H NMR (400 MHz, *d*-DMSO) δ 11.53 (s, 1H), 8.71 (dd, *J* = 22.8, 7.9 Hz, 2H), 7.82 (d, *J* = 1.5 Hz, 1H), 7.34–7.26 (m, 2H), 7.25–7.19 (m, 2H), 7.11–7.06 (m, 1H), 7.00–6.93 (m, 2H), 6.50 (d, *J* = 7.7 Hz, 1H), 5.51 (s, 1H), 5.11 (ABq, 2H), 4.76–4.69 (m, 1H), 4.42 (q, *J* = 7.8 Hz, 1H), 3.89 (s, 3H), 3.68 (t, *J* = 7.4 Hz, 2H), 3.16 (dd, *J* = 13.6, 4.5 Hz, 1H), 3.04 (dd, *J* = 13.8, 10.4 Hz, 1H), 2.24–2.14 (m, 1H), 1.90 (dq, *J* = 14.5, 7.2 Hz, 1H), 1.57 (s, 6H). ^13^C (126 MHz, *d*-DMSO): δ 202.7, 171.8, 162.0 (d, *J* = 243.0 Hz), 161.2, 153.6, 153.5, 145.3 (m), 143.4 (m), 141.2 (d, *J* = 7.5 Hz), 140.9 (m), 139.0 (d, *J* = 18.0 Hz), 137.8, 135.0, 134.4 (m), 129.9 (d, *J* = 8.4 Hz), 129.6, 125.3 (d, *J* = 2.6 Hz), 124.4, 120.0 (m), 118.0, 115.9 (d, *J* = 21.1 Hz), 113.1 (d, *J* = 20.7 Hz), 105.4, 101.0, 99.2, 74.7, 71.4, 55.0, 54.4, 53.1, 40.6, 36.5, 30.9 (t, *J* = 3.7 Hz), 28.2. ^19^F (377 MHz, *d*-DMSO): δ −113.8 (dt, *J* = 10.0, 4.9 Hz), −141.1 (dd, *J* = 23.1, 6.9 Hz), −157.9 (dd, *J* = 22.1, 7.0 Hz). HRMS (*m*/*z*): [M-H]^−^ calcd for C_35_H_33_F_5_N_6_O_7_: 743.2258; found: 743.2272.

##### *tert*-Butyl (*S*)-2-((*tert*-Butoxycarbonyl)­amino)-4-(5-oxo-1,5-dihydro-4*H*-1,2,4-triazol-4-yl)­butanoate (**S27**)

In a round-bottom flask, 2,4-dihydro-3*H*-1,2,4-triazol-3-one (3.7 g, 43.3 mmol, 1 equiv) was dissolved in DMF (74 mL, 20 vol) and cooled to 0 °C. To this, was added K_2_CO_3_ (8.6 g, 65.2 mmol, 1.5 equiv) and *tert*-butyl (*S*)-4-bromo-2-((*tert*-butoxycarbonyl)­amino)­butanoate (7.33 g, 21.7 mmol, 0.5 equiv). The reaction mixture was stirred at 25 °C for 4 h. Progress of the reaction was monitored by TLC. Upon completion, it was diluted with ice cold water (120 mL) and extracted with EtOAc (3 × 200 mL). The combined organic layer was washed with ice cold water (2 × 100 mL) followed by brine (100 mL), dried over Na_2_SO_4_ and concentrated under vacuum to give crude material as brown oil (∼7.8 g). The crude was purified by silica flash chromatography (20→25% EtOAc in pet-ether) to give **S27** (4.7 g, 32%) as off-white solid. R*_f_* = 0.7 (EtOAc/Pet-ether 3:2). ESI-MS (*m*/*z*): [M + H]^+^ calcd for C_15_H_26_N_4_O_5_: 343.20; found: 343.19.

##### *tert*-Butyl (*S*)-2-((*tert*-Butoxycarbonyl)­amino)-4-(5-oxo-1,5-dihydro-4*H*-1,2,4-triazol-4-yl)­butanoate (**S28**)

In a round-bottom flask, *tert*-butyl (*S*)-2-((*tert*-butoxycarbonyl)­amino)-4-(5-oxo-1,5-dihydro-4*H*-1,2,4-triazol-4-yl)­butanoate (4.7 g, 13.7 mmol, 1 equiv) was dissolved in DMF (47 mL, 10 vol) and cooled to 0 °C. To this, was added Cs_2_CO_3_ (6.69 g, 20.6 mmol, 1.5 equiv) and benzyl bromide (2.02 mL, 16.4 mmol, 1.2 equiv). The reaction mixture was stirred at 25 °C for 3 h. Progress of the reaction was monitored by TLC. Upon completion, it was diluted with ice cold water (100 mL) and extracted with EtOAc (3 × 200 mL). The combined organic layer was washed with ice cold water (2 × 100 mL) followed by brine (200 mL), dried over Na_2_SO_4_ and concentrated under vacuum to give crude material as brown oil (∼6.5 g). The crude was purified by silica flash chromatography (10→15% EtOAc in pet-ether) to give **S28** (4.3 g, 73%) as off-white solid. R*_f_* = 0.6 (EtOAc/pet-ether 2:3). ESI-MS (*m*/*z*): [M + H]^+^ calcd for C_22_H_32_N_4_O_5_: 433.25; found: 433.25.

##### (*S*)-2-Amino-4-(1-benzyl-5-oxo-1,5-dihydro-4*H*-1,2,4-triazol-4-yl)­butanoic Acid (**S29**)

To a stirred solution of *tert*-butyl (*S*)-2-((*tert*-butoxycarbonyl)­amino)-4-(5-oxo-1,5-dihydro-4*H*-1,2,4-triazol-4-yl)­butanoate (4.3 g, 9.95 mmol), 30% TFA in DCM (86 mL, 20 vol) was added at 0 °C and brought to room temperature. The resulting reaction mixture was stirred at 25 °C for 16 h. Progress of the reaction was monitored by LCMS. After completion of the reaction, it was concentrated under reduced pressure and triturated with diethyl ether to get **S29** (3.8 g, > 99%) as an off-white solid. The crude material was used without further purification. ESI-MS (*m*/*z*): [M + H]^+^ calcd for C_13_H_16_N_4_O_3_: 277.13; found: 277.13.

##### (*S*)-4-(1-Benzyl-5-oxo-1,5-dihydro-4*H*-1,2,4-triazol-4-yl)-2-((*tert*-butoxycarbonyl)­amino)­butanoic Acid (**S30**)

A solution of (*S*)-2-amino-4-(1-benzyl-5-oxo-1,5-dihydro-4*H*-1,2,4-triazol-4-yl)­butanoic acid (3.8 g, 13.7 mmol) in dioxane:H_2_O (1:1) (38 mL, 10 vol) was cooled to 0 °C and NaHCO_3_ (4.68 g, 55.1 mmol) was added, followed by the addition of (Boc)_2_O (9.47 mL, 41.3 mmol). The contents were stirred for 2 h at 25 °C. Progress of the reaction was monitored by TLC. After completion of reaction, it was diluted with water (38 mL, 10 vol) and extracted with EtOAc (3 × 100 mL). The combined organic layer was washed with ice cold water (3 × 60 mL), brine solution (100 mL) and dried over Na_2_SO_4_ and concentrated under vacuum to give **S30** (4.0 g) as an off-white solid. The crude material was used without further purification. ESI-MS (*m*/*z*): [M + H]^+^ calcd for C_18_H_24_N_4_O_5_: 377.18; found: 377.18.

##### *tert*-Butyl (*S*)-(5-(1-Benzyl-5-oxo-1,5-dihydro-4*H*-1,2,4-triazol-4-yl)-1-chloro-2-oxopentan-3-yl)­carbamate (**S31**)

A solution of (*S*)-4-(1-benzyl-5-oxo-1,5-dihydro-4*H*-1,2,4-triazol-4-yl)-2-((*tert*-butoxycarbonyl)­amino)­butanoic acid (4.0 g, 10.6 mmol) in THF (80 mL, 20 vol) was cooled to −10 °C and added triethylamine (1.92 mL, 13.8 mmol), followed by isobutyl chloroformate (1.65 mL, 12.8 mmol). The resulting reaction mixture was stirred at −10 °C for 30 min. The heterogeneous reaction mixture was filtered and washed with THF (20 mL). The filtrate was taken in a RBF and was cooled to −15 °C. To this, was added a freshly prepared diazomethane in diethyl ether (40 mL) dropwise at −10 °C. The resulting reaction mixture was stirred for 30 min at −10 °C. Progress of the reaction was monitored by TLC. After completion of reaction, it was quenched with acetic acid until it turns to colorless and diluted with water (160 mL, 40 vol) and extracted with EtOAc (3 × 200 mL). The combined organic layer was washed with brine solution (200 mL), dried over Na_2_SO_4_, concentrated under vacuum and the resulting yellow semisolid (4.0 g, crude) was used without further purification. R*_f_* = 0.5 (EtOAc/Pet-ether 3:2). ESI-MS (*m*/*z*): [M + H]^+^ calcd for C_19_H_24_N_6_O_4_: 401.19; found: 401.19. The obtained semisolid intermediate (4.0 g, 9.90 mmol) was dissolved in THF (80 mL, 20 vol) and the solution was cooled to −10 °C and 4 M HCl in dioxane was added (9.29 mL, 37.2 mmol). The resulting reaction mixture was stirred for 30 min at the same temperature. Progress of the reaction was monitored by TLC. After completion of reaction, it was concentrated and triturated with pentane (2 × 10 mL) to give **S31** (3.0 g) as a light yellow gum. The crude material was used without further purification. ESI-MS (*m*/*z*): [M + H]^+^ calcd for C_19_H_25_ClN_4_O_4_: 409.16; found: 409.57.

##### (*S*)-4-(3-Amino-5-chloro-4-oxopentyl)-2,4-dihydro-3*H*-1,2,4-triazol-3-one (**S32**)

To a solution of *tert*-butyl (*S*)-(5-(1-benzyl-5-oxo-1,5-dihydro-4H-1,2,4-triazol-4-yl)-1-chloro-2-oxopentan-3-yl)­carbamate (300 mg, 0.73 mmol) in toluene (6 mL, 20 vol) at 25 °C was added CF_3_SO_3_H (0.21 mL, 2.48 mmol). The contents were irradiated with microwave at 100 °C for 20 min. Progress of the reaction was monitored by TLC. After completion of reaction, it was concentrated under reduced pressure. The crude was diluted with water (6 mL, 20 vol) and extracted with EtOAc (3 × 10 mL). The combined organic layer was washed with ice cold water (3 × 10 mL), brine solution (10 mL) and dried over Na_2_SO_4_. The volatiles were removed under vacuum to give **S32** (300 mg, crude) as an off-white solid. This free amine was immediately coupled with the dipeptide. ESI-MS (*m*/*z*): [M + H]^+^ calcd for C_7_H_11_ClN_4_O_2_: 219.06; found: 219.12.

##### *N*-((*S*)-1-(((*S*)-1-Chloro-2-oxo-5-(5-oxo-1,5-dihydro-4*H*-1,2,4-triazol-4-yl)­pentan-3-yl)­amino)-3-(3-fluorophenyl)-1-oxopropan-2-yl)-4-methoxy-1*H*-indole-2-carboxamide (**S33**)

A solution of (*S*)-3-(3-fluorophenyl)-2-(4-methoxy-1*H*-indole-2-carboxamido)­propanoic acid (0.4 g, 1.12 mmol) in DMF (12 mL, 30 vol) was cooled to −5 °C, followed by the addition of (*S*)-4-(3-amino-5-chloro-4-oxopentyl)-2,4-dihydro-3*H*-1,2,4-triazol-3-one 9 (0.49 g, 2.25 mmol). The contents were stirred for 5 min at the same temperature followed by the addition of HATU (0.64 g, 1.68 mmol) and DIPEA (0.58 mL, 3.36 mmol). The resulting reaction mixture was stirred at −5 °C for 30 min. Progress of the reaction was monitored by TLC. After completion of reaction, it was diluted with water (16 mL, 40 vol) and extracted with EtOAc (3 × 30 mL). The combined organic layer was washed with ice cold water (3 × 20 mL), brine solution (30 mL) and dried over Na_2_SO_4_. The volatiles were removed under vacuum to give crude material. The crude was purified by silica flash chromatography (2→3% MeOH in DCM) to give **S33** (0.20 g, 32%) as yellow solid. ^1^H NMR (400 MHz, *d*-DMSO) δ 11.64 (bs, 1H), 11.53 (bs, 1H), 8.77 (d, *J* = 7.9 Hz, 1H), 8.70 (d, *J* = 7.6 Hz, 1H), 7.75 (d, *J* = 1.3 Hz, 1H), 7.35–7.28 (m, 2H), 7.27–7.18 (m, 2H), 7.09 (t, *J* = 8.0 Hz, 1H), 7.04–6.95 (m, 2H), 6.50 (d, *J* = 7.8 Hz, 1H), 4.72–4.62 (m, 1H), 4.45 (s, 1H), 4.40–4.32 (m, 1H), 3.89 (s, 3H), 3.63–3.48 (m, 2H), 3.18 (dd, *J* = 13.7, 5.0 Hz, 1H), 3.11–3.02 (m, 1H), 2.26–2.15 (m, 1H), 1.89–1.77 (m, 1H). ^19^F NMR (377 MHz, *d*-DMSO) δ −113.7 (m). HRMS (*m*/*z*): [M + H]^+^ calcd for C_26_H_26_ClFN_6_O_5_: 557.1710; found: 557.1706.

##### *N*-((*S*)-3-(3-Fluorophenyl)-1-oxo-1-(((*S*)-2-oxo-5-(5-oxo-1,5-dihydro-4*H*-1,2,4-triazol-4-yl)-1-(2,3,5,6-tetrafluoro-4-(2-hydroxypropan-2-yl)­phenoxy)­pentan-3-yl)­amino)­propan-2-yl)-4-methoxy-1*H*-indole-2-carboxamide (**13**)

Following GP1, 13 was obtained using *N*-((*S*)-1-(((*S*)-1-chloro-2-oxo-5-(5-oxo-1,5-dihydro-4*H*-1,2,4-triazol-4-yl)­pentan-3-yl)­amino)-3-(3-fluorophenyl)-1-oxopropan-2-yl)-4-methoxy-1*H*-indole-2-carboxamide (290 mg, 0.52 mmol) and 2,3,5,6-tetrafluoro-4-(2-hydroxypropan-2-yl)­phenol (234 mg, 1.04 mmol). The crude product was purified by RP-HPLC and chiral SFC to yield **13** (8 mg, 2%). ^1^H NMR (400 MHz, *d*-DMSO) δ 11.64 (bs, 1H), 11.52 (d, *J* = 2.4 Hz, 1H), 8.74 (dd, *J* = 12.8, 7.8 Hz, 2H), 7.73 (d, *J* = 1.4 Hz, 1H), 7.35–7.18 (m, 4H), 7.12–7.06 (m, 1H), 7.00–6.93 (m, 2H), 6.50 (d, *J* = 7.7 Hz, 1H), 5.50 (bs, 1H), 5.06 (q, *J* = 17.9 Hz, 2H), 4.72–4.64 (m, 1H), 4.41–4.32 (m, 1H), 3.89 (s, 3H), 3.65–3.48 (m, 2H), 3.16 (dd, *J* = 13.8, 5.0 Hz, 1H), 3.12–3.03 (m, 1H), 2.25–2.14 (m, 1H), 1.88–1.77 (m, 1H), 1.56 (s, 6H). ^19^F (377 MHz, *d*-DMSO): δ −113.8 (d, *J* = 9.6 Hz), −141.1 (d, *J* = 21.9 Hz), 157.8 (dd, *J* = 22.0, 7.1 Hz). HRMS (*m*/*z*): [M + H]^+^ calcd for C_35_H_33_F_5_N_6_O_7_: 745.2404; found: 745.2391.

##### (1*R*,2*S*,5*S*)-*N*-((*S*)-4-Chloro-3-oxo-1-((*S*)-2-oxopyrrolidin-3-yl)­butan-2-yl)-3-((*S*)-3,3-dimethyl-2-(2,2,2-trifluoroacetamido)­butanoyl)-6,6-dimethyl-3-azabicyclo­[3.1.0]­hexane-2-carboxamide (**S34**)

*tert*-Butyl ((*S*)-4-chloro-3-oxo-1-((*S*)-2-oxopyrrolidin-3-yl)­butan-2-yl)­carbamate (2.0 g, 6.58 mmol) was deprotected according to GP2. Subsequently, starting from (1*R*,2*S*,5*S*)-3-((*S*)-3,3-dimethyl-2-(2,2,2-trifluoroacetamido)­butanoyl)-6,6-dimethyl-3-azabicyclo­[3.1.0]­hexane-2-carboxylic acid (1.1 g, 3.14 mmol) and ((*S*)-3-((*S*)-2-amino-4-chloro-3-oxobutyl)­pyrrolidin-2-one (TFA salt) (1.0 g, 3.14 mmol) GP3 was followed and the crude product was purified by flash column chromatography to yield **S34** (800 mg, 53%). ^1^H NMR (400 MHz, *d*-DMSO) δ 9.39 (d, *J* = 8.5 Hz, 1H), 8.74 (d, *J* = 8.1 Hz, 1H), 7.60 (s, 1H), 4.63 (d, *J* = 0.9 Hz, 2H), 4.51–4.44 (m, 1H), 4.42 (d, *J* = 8.5 Hz, 1H), 4.23 (s, 1H), 3.71–3.67 (m, 2H), 3.15 (t, *J* = 9.0 Hz, 1H), 3.11–3.00 (m, 1H), 2.42–2.34 (m, 1H), 2.16–2.07 (m, 1H), 2.00–1.90 (m, 1H), 1.68–1.58 (m, 2H), 1.54 (dd, *J* = 7.6, 5.3 Hz, 1H), 1.38 (d, *J* = 7.6 Hz, 1H), 1.03 (s, 3H), 0.98 (s, 9H), 0.86 (s, 3H). ESI-MS (*m*/*z*): [M + H]^+^ calcd for C_24_H_34_ClF_3_N_4_O_5_: 551.22; found: 551.22.

##### (1*R*,2*S*,5*S*)-3-((*S*)-3,3-Dimethyl-2-(2,2,2-trifluoroacetamido)­butanoyl)-6,6-dimethyl-*N*-((*S*)-3-oxo-1-((*S*)-2-oxopyrrolidin-3-yl)-4-(2,3,5,6-tetrafluoro-4-(2-hydroxypropan-2-yl)­phenoxy)­butan-2-yl)-3-azabicyclo­[3.1.0]­hexane-2-carboxamide (**14**)

Following GP1, 14 was obtained using (1*R*,2*S*,5*S*)-*N*-((*S*)-4-chloro-3-oxo-1-((*S*)-2-oxopyrrolidin-3-yl)­butan-2-yl)-3-((*S*)-3,3-dimethyl-2-(2,2,2-trifluoroacetamido)­butanoyl)-6,6-dimethyl-3-azabicyclo­[3.1.0]­hexane-2-carboxamide (600 mg, 1.09 mmol) and 2,3,5,6-tetrafluoro-4-(2-hydroxypropan-2-yl)­phenol (244 mg, 1.09 mmol). The crude product was purified by flash column chromatography, RP-HPLC and chiral SFC to yield **14** (80 mg, 10%). ^1^H NMR (400 MHz, *d*-DMSO) δ 9.41 (d, *J* = 6.1 Hz, 1H), 8.69 (d, *J* = 8.3 Hz, 1H), 7.61 (s, 1H), 5.52 (s, 1H), 5.24 (s, 2H), 4.50 (ddd, *J* = 11.9, 8.4, 3.4 Hz, 1H), 4.42 (d, *J* = 6.2 Hz, 1H), 4.23 (s, 1H), 3.91 (dd, *J* = 10.3, 5.5 Hz, 1H), 3.69 (d, *J* = 10.4 Hz, 1H), 3.15 (t, *J* = 9.0 Hz, 1H), 3.05 (td, *J* = 9.3, 7.1 Hz, 1H), 2.38 (dq, *J* = 14.3, 5.6 Hz, 1H), 2.10 (dt, *J* = 13.6, 7.9 Hz, 1H), 1.96 (ddd, *J* = 13.7, 11.8, 3.8 Hz, 1H), 1.68–1.52 (m, 8H), 1.35 (d, *J* = 7.6 Hz, 2H), 1.02 (s, 3H), 0.97 (s, 9H), 0.86 (s, 3H). ^13^C NMR (101 MHz, *d*-DMSO) 203.2, 178.4, 171.3, 167.4, 156.9 (q, *J* = 36.9 Hz), 145.6 (m), 143.2 (m), 141.2 (d, *J* = 18.1 Hz), 138.7 (d, *J* = 17.7 Hz), 134.5 (m), 120.0 (m), 115.8 (q, *J* = 288.0 Hz), 74.9, 71.4, 60.2, 58.1, 53.2, 47.6, 37.0, 34.6, 30.9 (t, *J* = 3.8 Hz), 30.4, 27.3, 27.2, 26.2, 25.8, 18.7, 12.3. ^19^F (377 MHz, *d*-DMSO): δ −72.9 (s), −141.1 (d, *J* = 19.3 Hz), −158.2 (dd, *J* = 22.1, 7.0 Hz). HRMS (*m*/*z*): [M + H]^+^ calcd for C_33_H_41_F_7_N_4_O_7_: 739.2936; found: 739.2930.

#### In Vivo Pharmacokinetics in Syrian Golden Hamsters

All animal experiments were performed following the protocols evaluated and approved by the Institutional Animal Ethics Committee (IAEC) of TheraIndx Lifesciences Pvt Ltd. Bangalore (Ethics Approval Number: IAEC/27/2024/304). For in vivo PK studies, 6–8 weeks old female Syrian golden hamsters were used. Animals were fasted for 8–10 h and were fed 4 h post animal dosing in the case of PO administration. As vehicle for 1 and 8, 10% DMSO, 20% PEG400, 65% PG and 5% PBS pH 7.4 was used. As vehicle for 7 and 14, 5% DMSO, 65% PG and 30% normal saline was used. The vehicle for ritonavir oral dosing was 5% DMSO, 65% PG and 30% normal saline. Animals were dosed either 1) intravenously through slow infusion during 30 min via cephalic vein, 2) intraperitoneally, 3) subcutaneously or 4) orally by gavage. All animals received ritonavir by oral administration 30 min prior to dosing. Post dose, serial blood samplings were collected (30–50 μL) from lateral saphenous vein by using 25-gauge needle at time points 15 min, 30 min, 1 h, 2 h, 4 h, 6 h, 8 h, 12 and 24 h. Blood was collected in 1.5 mL Eppendorf tubes containing 0.010 mL of 10% K_2_EDTA, mixed gently and placed on ice, followed by centrifugation at 10000 rpm for 10 min. Plasma was harvested and stored at −80 °C. Compound concentrations were quantified in plasma by LCMS7MS using a fit for purpose bioanalytical method. PK data analysis was performed using noncompartmental methods in WinNonlin.

## Supplementary Material





## Notes

Experimental details for in vitro assays, in vivo studies and binding mode predition; antiviral efficacy and cytotoxicity data; biochemical human CTSB, mouse CTSL, mouse CTSB and hamster CTSL activity data; predicted Michaelis complexes, inhibition data of a protease panel; mouse, hamster and human liver microsomal stability data; mouse and human hepatocyte stability data; LCMS analysis of a mouse liver microsomal stability assay; proposed CYP-mediated metabolism; predicted binding mode of **13**; hamster hepatocyte stability data with ritonavir; in vivo PK parameters; ^1^H NMR, ^13^C NMR and ^19^F NMR spectra of final compounds; analytical HPLC spectra of final compounds (PDF)
Molecular formula strings of the target compounds (CSV)

## ABBREVIATIONS

CPE: cytopathic effect
CTS: cathepsin
CYP: cytochromes P450
M^pro^: main protease
PK: pharmacokinetics.
