# Supplementary material for: Peptidomimetic Phenoxymethyl Ketone Warheads as Potent Dual-Mode Inhibitors against SARS-CoV‑2 Mpro and Cathepsin
Source: J Med Chem. 2025 May 26;68(11):10953–69. doi: 10.1021/acs.jmedchem.4c03147 (PMC12169608; doi:10.1021/acs.jmedchem.4c03147)
Supplement: Supplementary file 1 [file jm4c03147_si_001.pdf]

Supporting Information

Peptidomimetic phenoxymethyl ketone warheads as potent dual-mode inhibitors against SARS-CoV-2 M^{pro} and cathepsin

Miriam R.B. Porzberg[1], G.J. Mirjam Groenewold[2], Heyrhyoung Lyoo[3], Alexander K.M.H. Jakob[1], Willem H.C. Titulaer[1], Lorenzo Cavina[1], Katrien C.K. Poelaert[3], Marleen Zwaagstra[3], Cindy E.J. Dieteren[4], Jaap G.H. Lemmers[4], S. Hakim Hamdani[4], Bernd N.M. van Buuren[4], Bart Ackerschott[4], Johannes J. Platteeuw[5], Joey Michorius[5], Byron E.E. Martina[6], Martin C. Feiters[1], Daniel Gironés[1,4], Frank J.M. van Kuppeveld[3], Martijn J. van Hemert[2], Floris P.J.T. Rutjes[1,*]

[1]Institute for Molecules and Materials, Radboud University, Heyendaalseweg 135, 6525 AJ Nijmegen, The Netherlands

[2]Molecular Virology Laboratory, Leiden University Center for Infectious Diseases, Leiden University Medical Center, Albinusdreef 2, 2333ZA Leiden, The Netherlands

[3]Virology Section, Division of Infectious Diseases & Immunology, Department of Biomolecular Health Sciences, Faculty of Veterinary Medicine, Utrecht University, Yalelaan 1, 3584 CL Utrecht, The Netherlands

[4]Protinhi Therapeutics, Transistorweg 5, 6534 AT Nijmegen, The Netherlands

[5]Avivia BV, Transistorweg 5, 6534 AT Nijmegen, The Netherlands

[6]Artemis Bioservices, Molengraaffsingel 10, 2629 JD Delft, The Netherlands

*floris.rutjes@ru.nl

Table of contents

1.1 Bioassay methods S2

1.2 Supporting Figures S8

1.3 NMR spectra S18

1.4 HPLC spectra S38

1.5 References S44

1.1 Bioassay methods

SARS-CoV-2 M^{pro} biochemical assay:
M^{pro} enzymatic reactions were performed in reaction buffer containing 20 mM TRIS HCl pH 7.5, 150 mM NaCl, 1 mM EDTA, 1 mM TCEP, 0.25% glycerol, 0.02% BSA and 0.003% Tween-20. Recombinant SARS-CoV-2 3C-like main protease according to PDB-ID 7BQY (6.6 nM final assay concentration) was added to Greiner small volume 384-well black plates containing 30 µM Dabcyl-KTSAVLQSGFRKM-E(Edans)-amide substrate solution (Biosynthan GmbH, dissolved at 20 mM in 100% DMSO). 100 µM ML188 was included to generate no activity control samples. 11-point full concentration response curves were generated directly in assay plates using a Tecan D300, providing concentrations between 6.3 pM and 250 µM. DMSO was normalized to 2.5% across the entire plate. Plates were immediately transferred to a BMG CLARIOstar plate reader and the change in fluorescence intensity was monitored for 30 min at 15 sec intervals.

Cathepsin biochemical assays:
Cathepsin enzymatic reactions were conducted in assay buffer with a final DMSO concentration of 1% per well. For hCTSL, assay were performed in 50 mM MES, 5 mM DTT, 1 mM EDTA, 0.005% (w/v) Brij-35, pH 6.0. For mCTSL, assays were conducted in 25 mM MES, 5 mM DTT, pH 6.0. hCTSB and mCTSB assays were performed in 25 mM MES, pH 5.0. Compounds (10 mM stocks in 100% DMSO) and controls (100% DMSO) were prediluted and 10 µL was added in duplicate to a Thermo Scientific™ Nunc™ F96 MicroWell™ black PS micro plate. Starting at a concentration of 50 µM, 3-fold serial dilutions (keeping DMSO concentration constant at 10%) were used to generate 8-point dose response curves Ten µM E-64 (10 mM stock in 100% DMSO (Alfa Aesar™)) final assay plate well concentration was used to generate no fluorescence signal increase and an 8-point dose response curve (0.457 – 1000 nM) was used as an internal compound control. Substrate pre-dilution to 37.5 µM (10 mM stock Z-Leu-Arg-AMC x HCl (Bachem Biochemica) in 100% DMSO) was prepared in assay buffer and transferred to the wells of a separate micro plate. As a positive fluorescence control 37.5 µM AMC (50 mM stock in 100% DMSO (Alfa Aesar™)) in assay buffer was used in duplicate. The substrate pre-dilution plate was pre-incubated at 25 °C in a micro plate fluorescence spectrophotometer (Molecular Devices, Gemini XPS) just before the enzyme-compound assay plate incubation. Assay plate final well concentration for substrate and fluorescence control was 15 µM. Recombinant hCTSL enzyme (R&D Systems®) was activated and pre-diluted to 0.030 ng/µL according to the manufacturer's protocol. Fifty µL was added to the wells of the assay plate containing the compound pre-dilutions, and 50 µL assay buffer was used as a negative control. The assay plate was then incubated for 10 minutes at 25 °C in a micro plate fluorescence spectrophotometer. Assay plate final well concentration was 15 pg/µL. Finally, 40 µL per well was transferred from the substrate pre-dilution plate to the assay plate and an optical adhesive seal was applied. Final well volume was 100 µL. Fluorescence signal was measured for 10 minutes at 1 minute intervals in a micro plate fluorescence spectrophotometer at 25°C (λ_{ex} = 346 nm, λ_{em} = 440). Reaction speeds were determined using the linear part of the fluorescence signal curves. The DMSO control (DC) reaction speed was used to calculate the activity and inhibitory potency of the samples. CDD Vault was used to calculate the IC_{50} values using the Levenberg–Marquardt algorithm to fit the Hill equation for dose-response data to the generated inhibition curves.

CPE reduction assays:
Cell culture: Vero E6 cells were cultured as previously described at 37 °C under 5% CO_2 and they were tested regularly for the absence of mycoplasma contamination.[1]
Cell viability (CPE reduction) assay: Vero E6 cells were seeded in 96-well plates at a density of 6 x 10^3 cells per well 1 day prior to the experiment. Cells were then treated with 2-fold serial dilutions of testing compounds starting at concentrations between 75 and 0.25 µM. At 4 days post infection, the viability of

(infected and non-infected) compound-treated cells was measured using CellTiter 96® aqueous MTS reagent (Promega). MTS absorbance was measured at 495 nm with an EnVision multiplate reader (PerkinElmer). CPE reduction assays with SARS-CoV and MERS-CoV were performed as described previously.[2]

Virus infections: Infection experiments with SARS-CoV-2/Leiden-0002 (isolated at LUMC during the first wave of the COVID-19 pandemic in March 2020 (GenBank: MT510999.1)) were performed in the LUMC biosafety level 3 facilities as described previously.[1]

Data analysis: Data analysis was performed using Microsoft® 365 Excel and GraphPad Prism® 9.3.1 (GraphPad Software Inc., CA). CC_{50} and EC_{50} values were determined via non-linear regression (sigmodial, 4PL, X is concentration).

Cellular M^{pro} assay:

We set up a cell based SARS-CoV-2 main protease (M^{pro}) activity assay based on the previously reported cell-based enterovirus 3CL protease assay.[3] The assay is based on the induction of expression of a firefly luciferase reporter by a chimeric transcription factor, in which M^{pro} is flanked by M^{pro} cleavage sites inserted between the GAL4 binding domain and the VP16 activation domain. Firefly luciferase expression is dependent on cleavage of the transcription factor by M^{pro}. Briefly, HEK293T cells were seeded at 15,000 cells per well in a Poly-L-Lysine coated 96-well plate and incubated overnight at 37 °C. Next day, compound dilutions were prepared in 10% DMEM at 10 and 50 µM for screening or in a concentration range from 100 µM (1/3 dilution). Medium in the cell-seeded plates was replaced to 100 µL of compound containing medium. 10 µL transfection mix containing pG5luc mix (50 ng pBIND-Mpro-VP16 (SARS2) plasmid (WT or C145A M^{pro}), 50 ng pG5luc plasmid, up to 5 µL OptiMEM) and lipofectamine mix (0.5 µL lipofectamine 2000, 4.5 µL OptiMEM) was added to each well and mixed by pipetting up and down once, followed by incubation for 20-24 hours. Both Firefly and Renila luciferase expression was measured by using the Dual-Luciferase® Reporter Assay System (Promega) according to the manufacturer's protocol. Results were plotted as FLuc/RLuc ratio. In parallel, cell viability was determined by an MTS assay. Herein, CellTiter 96® AQueous One Solution (Promega) was applied on top of the existing medium. After 1-2 h incubation at 37 °C, OD490nm was measured.

Pseudovirus neutralization assay:

Neutralization assays using luciferase-expressing VSV (ΔG) pseudotyped with Spike from SARS-CoV-2 were performed as described.[4] Briefly, VeroE6 cells were seeded in 96-well plates at 25.000 cells/well in 100 µL and incubated overnight at 37 °C. Compound dilutions were prepared in 10% DMEM at 10 µM for screening or concentration ranges. SARS2pp virus dilution was prepared 1:50 in 1% DMEM. Medium was removed from the wells, 50 µL of compound was added to the cells and plates were incubated for 1 h at 37 °C. After that, 50 µL virus dilution was added to the wells, followed by incubation overnight at 37 °C. For firefly luciferase readout, medium was removed from the wells and 60 µL 5x diluted lysis buffer in MQ was added to each well and incubated for 30 min at rt. 100 µL homemade firefly substrate (UU-FLAR) was prepared per well. Subsequently, 25 µL lysate was added to a white 96-well plate and firefly luciferase output was measured with a luminometer. Cytotoxicity was determined as described previously.

LogD:

In vitro LogD determination at pH 7.4 was carried out using the octanol buffer partitioning method. Equal volumes of sodium phosphate buffer (10 mM) and n-octanol were added to a separation funnel and mixed thoroughly by shaking and inverting the funnel several times. The two layers were allowed to separate for 2 days and then dispensed into separate glass bottles. 500 µL of organic phase (1-octanol) was added to each well of a 2 mL deep well plate, followed by 500 µL of buffer and 15 µL of test substance was added

(dissolved at 10 mM in 100% DMSO). The plate was vortexed for 1 h on a plate shaker at 1200 rpm. After incubation, the samples were allowed to equilibrate for 20 min and then centrifuged at 4000 rpm for 30 min for complete phase separation and analyzed by LC-UV (Shimadzu UFLC, Waters xBridge C18 column, 50*4.6 mm, 3.5 µM; A: 10 mM ammonium acetate/0.1% formic acid in MQ; B: 100% acetonitrile).

Kinetic Solubility:
4 µL of 10 mM DMSO stock from the stock plate was added to the deep well plate containing 396 µL of PBS buffer pH 7.4 (100 µM final concentration, 1.0% DMSO content). The well-sealed sample plate was vortexed at 800 rpm for 24 h on a thermomixer at room temperature. At the end of the incubation period, the sample plate was centrifuged at 4000 rpm for 10 min and analyzed in LC-UV (Shimadzu UFLC, Waters xBridge C18 column, 50*4.6 mm, 3.5 µM; A: 10 mM ammonium acetate/0.1% formic acid in MQ; B: 100% acetonitrile) against calibration curve (CC). Solubility (µM) was obtained against the CC curve.

Plasma Protein Binding:
In vitro plasma protein binding studies were carried out by ultra-centrifugation using mouse plasma. 2.4 mL of plasma containing internal QC (Warfarin, 10 mM) was preincubated for 20 min at 37 °C. 6 µL of test compound stock solution (800 µM) was added and mixed properly. 25 µL of plasma was separated and crashed with 300 µL of acetonitrile containing internal standard. 400 µL of blank plasma was transferred to a 0.5 mL Beckman tubes (part no. 344625) and centrifuged for 3 h, 627000 g, 37 °C. After 45 min and after 3 h, 25 µL of supernatant samples was separated and crashed with 300 µL of acetonitrile containing internal standard. 25 µL of blank plasma was added to crashed centrifuged samples for matrix matching. All stability and centrifuged samples were vortexed at 1000 rpm for 5 min and centrifuged for 10 min, 4000 rpm. The supernatant was separated, diluted 2-fold with MQ and analyzed by LC-MS/MS.

Plasma Stability:
In vitro plasma stability determination was carried out in mouse CD1 plasma. The frozen plasma was thawed at room temperature and centrifuged at 1400 rcf 4 ˚C, for 15 min. Approximately 90% of the clear supernatant fraction was transferred to a separate tube and was used for the assay. Test compound stocks of 2 µM were prepared by diluting in plasma. 500 µL of plasma containing the test compound was incubated for 120 min at 37 ˚C in a shaker water bath with gentle shaking. 50 µL aliquots of sample at 0, 15, 30, 60 and 120 min were precipitated immediately with 300 µL acetonitrile containing internal standard and were centrifuged at 4000 rcf, 4 ˚C for 10 min. 150 µL of supernatant was diluted with 150 µL of water and analyzed by LC-MS/MS (EXION LC, QTRAP 4500, KINETEX C18 column, 100 Å, 5 µM; A: 10 mM ammonium acetate/0.1% formic acid in MQ; B: MeOH/MeCN 1:1).

Hepatocyte Stability:
In vitro hepatocyte stability assays were carried out using cryopreserved hepatocytes (Thermo Fisher Scientific). Hepatocytes were thawed, resuspended in prewarmed InvitroGRO HT medium and the cell count was taken using a hemacytometer. 200 µL of hepatocytes (0.8 x 10^6 cells/mL) were added to the wells (48 well plate) and pre-incubated for 30 min and then 200 µL of compound stock (2 µM, water/MeCN 1:1, 0.1% final DMSO concentration) was added to it and incubated in a CO_2 incubator for 120 min. The plate was vortexed at 500 rpm. At time points 0, 15, 30, 60, 90 and 120 min, 50 µL of incubation mixture was precipitated with 200 µL of acetonitrile containing internal standard (propranolol). At the end of the experiment assay samples were centrifuged and supernatants were submitted for LC-MS/MS analysis (EXION LC, TRIPLE-QUADE-4500; A: 10 mM ammonium acetate/0.1% formic acid in MQ; B: MeOH/MeCN 1:1). In case of Ritonavir co-administration, 75 µL of buffer containing 1 µM Ritonavir was added to 150 µL cells and pre-incubated for 10 min. Subsequently, 75 µL of 4 µM test compound solution was added

(final concentrations: 0.25 µM Ritonavir, 1 µM test compound). The plate was incubated, vortexed and analyzed as described above.

Microsomal Stability:

In vitro microsomal stability assays were carried out using mouse liver microsomes with either a) 10 µM test compound concentration, 1.0 mg/mL final protein concentration and 2.5 µM Ritonavir or b) 1 µM test compound concentration and 0.5 mg/mL final protein concentration. 75 µL liver microsomes (3.33 mg/mL) were pre-incubated with 2.5 µL test compound solution (100 µM, water/MeCN 1:1) and 85 µL 100 mM potassium phosphate buffer pH 7.4 for 10 min at 37 °C. 60 min samples without cofactor were obtained by mixing 32.5 µL of pre-incubation solution and 17.5 µL 100 mM potassium phosphate buffer pH 7.4, followed by incubation for 60 min at 37 °C. 0 min reference samples were obtained by mixing 16.25 µL of pre-incubation solution with 200 µL MeCN containing internal standard and 8.75 µL cofactor (NADPH, final concentration 1 mM). The remaining pre-incubation solution was mixed with 62 µL cofactor (NADPH, final concentration 1 mM), followed by incubation for 60 min at 37 °C. For analysis, 25 µL aliquots of the incubated samples were mixed with 200 µL MeCN containing internal standard. Subsequently, samples were vortexed for 5 min at 1200 rpm and centrifuged for 10 min at 4000 rpm. The supernatant was diluted 2-fold with MQ and injected on LC-MS/MS (EXION LC, QTRAP 4500, KINETEX C18 column, 100 Å, 5 µM; A: 10 mM ammonium acetate/0.1% formic acid in MQ; B: MeOH/MeCN 1:1). In case of Ritonavir co-administration, 2.5 µL Ritonavir (100 µM, water/MeCN 1:1) was added to 75 µL liver microsomes (1.666 mg/mL) and 85 µL 100 mM potassium phosphate buffer pH 7.4, followed by pre-incubation for 15 min at 37 °C. Subsequently, 2.5 µL of test compound solution was added and the plate was incubated, vortexed and analyzed as described above.

Caco-2 permeability assay:

In vitro evaluation of apparent permeability was performed using 21 day cultured Caco-2 cells monolayer in 96-well format (American Type Culture Collection). 10 mM stock solutions in DMSO containing control compounds propranolol (high permeability), atenolol (low permeability) and digoxin (Pgp substrate) were diluted to 10 µM solutions in HBSS buffer pH 7.4, and 10 mM test compound solutions in DMSO were diluted with HBSS buffer pH 7.4 to a final concentration of 2 µM. 250 µL DMEM was added to the basal compartment of 96-well multi-screen plates (poly carbonate high pore density, Millipore, 0.4 µm pore size, 0.11 cm^2 active membrane area) and 12000 cells/well (0.16 x 10^6 cells/mL) were seeded in the apical wells, followed by CO_2 incubation at 37 °C for cell proliferation. Every alternate day, the utilized medium was replenished by fresh medium. On the day of the assay, medium was removed and washed with HBSS buffer. Plates were subsequently incubated with HBSS buffer for 30 min in an incubator and wells with TEER values greater than 230 $ohm.cm^2$ were selected for the assay. 75 µL of test compound in HBSS was added to apical wells and 250 µL of HBSS buffer and 1% DMSO was added to basal wells. Apical to basal permeability samples were collected at 120 min. 250 µL of test compound in HBSS was added to basal wells and 75 µL of HBSS buffer and 1% DMSO was added to apical wells. Basal to apical permeability samples were collected at 120 min. For sample processing, single point calibration curve in HBSS buffer was used. 25 µL of assay samples were precipitated with 300 µL MeCN containing internal standard (Telmisartan & Metoprolol), vortexed for 5 min at 850 rpm and centrifuged at 3500 rpm for 5 min. After centrifugation, 150 µL of supernatant was diluted with 150 µL MQ and submitted for LC-MS/MS analysis (EXION LC, QTRAP 6500+, Phenomenex C18 column, 50*4.6, 5 µM; A: 10 mM ammonium acetate/0.1% formic acid in MQ; B: MeOH/MeCN 1:1).

Metabolite Identification assay:
Identification of metabolites was carried out in mouse liver microsome at a final protein concentration of 1 mg/mL. 2.5 µL of test compound (1 mM) was added to 75 µL mouse liver microsomes at 3.33 mg/mL and 85 µL of 100 mM potassium phosphate buffer pH 7.4 pre-incubated for 10 min at 37 °C. The mixture was pre-incubated for 17 min at 37 °C in an incubator. For the 0 min sample, 16.4 µL of pre-incubation mixture was crashed in 300 µL with 8.6 µL of NADPH (1 mM). 32.5 µL of pre-incubation mixture and 17.5 µL of 100 mM potassium phosphate buffer pH 7.4 was kept for incubation. 62 µL NADPH (2.85 mM) was added to the pre-incubation mixture. The reaction mixture was stopped at 5, 15, 30, 45 and 60 min by taking out 25 µL of pre-incubation reaction mixture into 300 µL of crash plate. The samples were vortexed for 5 min at 850 rpm and centrifuged for 10 min at 4000 rpm. The supernatant was separated, diluted 2-fold with MQ and submitted for LC-MS/MS analysis (Agilent 1200 RRLC, QTRAP 5500, EMS+EPI, EPI (Fragmentation), MRM, Phenomenex C18 column, 50*4.6, 5 µM; A: 0.1% formic acid in MQ; B: 100% acetonitrile).

Protease Panel:
Calpain-1, Caspase 2, Cathepsin D and Neutrophil Elastase 2 assays were performed by Eurofins Discovery using the following assay conditions:

Protease	Substrate	Source	Pre-incubation Time/Temp	Incubation Time/Temp	Incubation Buffer	Quantitation Method
Calpain-1	0.05% Casein-FITC	Human erythrocytes	-	30 min, 37 ˚C	50 mM Tris-HCl, pH 7.4	Spectrofluorimetric quantitation of Peptide-FITC
Caspase 2	25 µM Z-VDVAD-AFC	Human recombinant E. coli	15 min, 37 ˚C	60 min, 37 ˚C	50 mM HEPES, pH 7.4, 100 mM NaCl, 0.1% CHAPS, 1 mM EDTA, 10% glycerol, 10 mM DTT	Spectrofluorimetric quantitation of AFC
Cathepsin D	20 µM MOCAc-Gly-Lys-Pro-Ile-Leu-Phe-Phe-Arg-Leu-Lys(Dnp)-D-Arg-NH_2	Human liver	15 min, 37 ˚C	10 min, 37 ˚C	50 mM CH_3COONa, pH 4.0	Spectrofluorimetric quantitation of MOCAc-Gly-Lys-Pro-Ile-Leu-Phe
Neutrophil Elastase 2	200 µM N-MeOSuc-Ala-Ala-Pro-Val-pNA	Human neutrophils	15 min, 25 ˚C	60 min, 25 ˚C	62.5 mM HEPES, pH 7.8, 625 mM NaCl, 1.25 mg/ml BSA	Spectrophotometric quantitation of pNA

Thrombin and trypsin assays were performed as previously reported.[5-6] Continuous fluorometric assays were done in black 96-well V-bottom plates (Greiner Bio-One) using a BMG Labtech Fluostar OPTIMA microtiter fluorescence plate reader (excitation = 355 nm; emission = 460 nm). Inhibitors were preincubated with thrombin (10 nM, Sigma-Aldrich) or trypsin (1 nM, Sigma-Aldrich) in assay buffer (50 mM Tris-HCl pH 7.5, 150 mM NaCl, 0.05% Tween 20) for 15 min. Enzymatic cleavage was initiated by the addition of the substrate Boc-Val-Pro-Arg-AMC (final concentration: 50 µM, Bachem). Enzyme activities were monitored for 15 min and determined as a slope of relative fluorescence units per second (RFU/s). Camostat mesylate served as inhibition control. All experiments were performed in triplicate and percentage inhibition was calculated as the mean of the values with standard deviations. Values are reported in relation to an uninhibited control.

Binding mode prediction:

<u>Covalent complexes</u>: A covalently bound M^{pro}-ligand structure with a 3-fluorophenyl and 2-indole cap as respectively P2- and P3-moiety (PDB ID: 6M0K[7]) was processed with MOE[8] to build missing residues, remove non-drug relevant ligands such as DMSO and protonate the homodimer at pH 7.4. Residues and ligands were parameterized with the Amber14:EHT force field,[9-10] protein residues further than 9 Å from the covalently bound ligand fixed while residues within 9 Å were tethered after which an energy minimization was performed using standard settings. Peptidomimetic P1-sidechains to be sampled were then built on the covalently bound ligand sampling all staggered conformations of the χ_1& χ_2 torsion angles. Binding modes that upon visual inspection did not affect or positively affect the hydrogen bonding network of the protein-ligand complex were chosen as representative binding modes for the ligand.

<u>Non-covalent complexes</u>: To elucidate if there is an enthalpic/K_M role of the warhead/P1' of compound **1**, non-covalent structures were modelled. The catalytic dyad was prepared as anionic cysteine (Cym) and protonated histidine (Hip) and the covalent ligand:protein bond was removed. The same tethered energy minimization protocol described *vide supra* was used for geometry optimizations while ligands were built in the active site using reference structures. Molecular surfaces were calculated with MOE and coloured by Poisson-Boltzman electrostatics. For M^{pro}, the ligand was built in the 6M0K complex using 7RXS[11] only to guide the placement of the tetrafluorophenol since superpositioning did not indicate induced fit effects of the warhead (benzothiazole-ketone warhead in 7RXS). For CTSL, 8GX2 (chain B) was chosen as model to build the ligand from using 7W33[11] to aid the P2-fluorophenyl sidechain placement. 8GX2 was chosen since it contains a P3-cap (benzofuran) and a P1 δ-lactam while exploring the S1' with a cyclopropyl from an α-ketoamide while 7ZVF[12] indicates crystal contacts of 8GX2 are negligible for interpretation of the P3-cap. For CTSB, 6AY2[13] was used to build the Michaelis complex using 8HE9[14] to direct the warhead placement.

1.2 Supporting Figures

Table S1. Antiviral efficacy (pEC_{50}) as determined by CPE reduction assay with SARS-CoV-2 in Vero E6 cells and cytotoxicity (pCC_{50})**.**

**Compound**	**pEC_{50} (CPE)**	**pCC_{50} (CPE)**
1	6.89	< 6.00
2	4.66	< 4.13
3	7.15	< 6.30
4	5.65	< 4.60
5	5.93	< 5.00
6	5.72	< 4.60
7	7.30	< 6.30
8	6.80	< 5.90
9	5.92	< 5.00
10	6.92	< 5.90
11	6.12	< 5.30
12	6.19	< 5.00
13	5.78	< 5.00
14	7.40	< 6.30
15	7.40	< 5.90
16	7.40	< 6.60
Nirmatrelvir	7.00	< 6.13

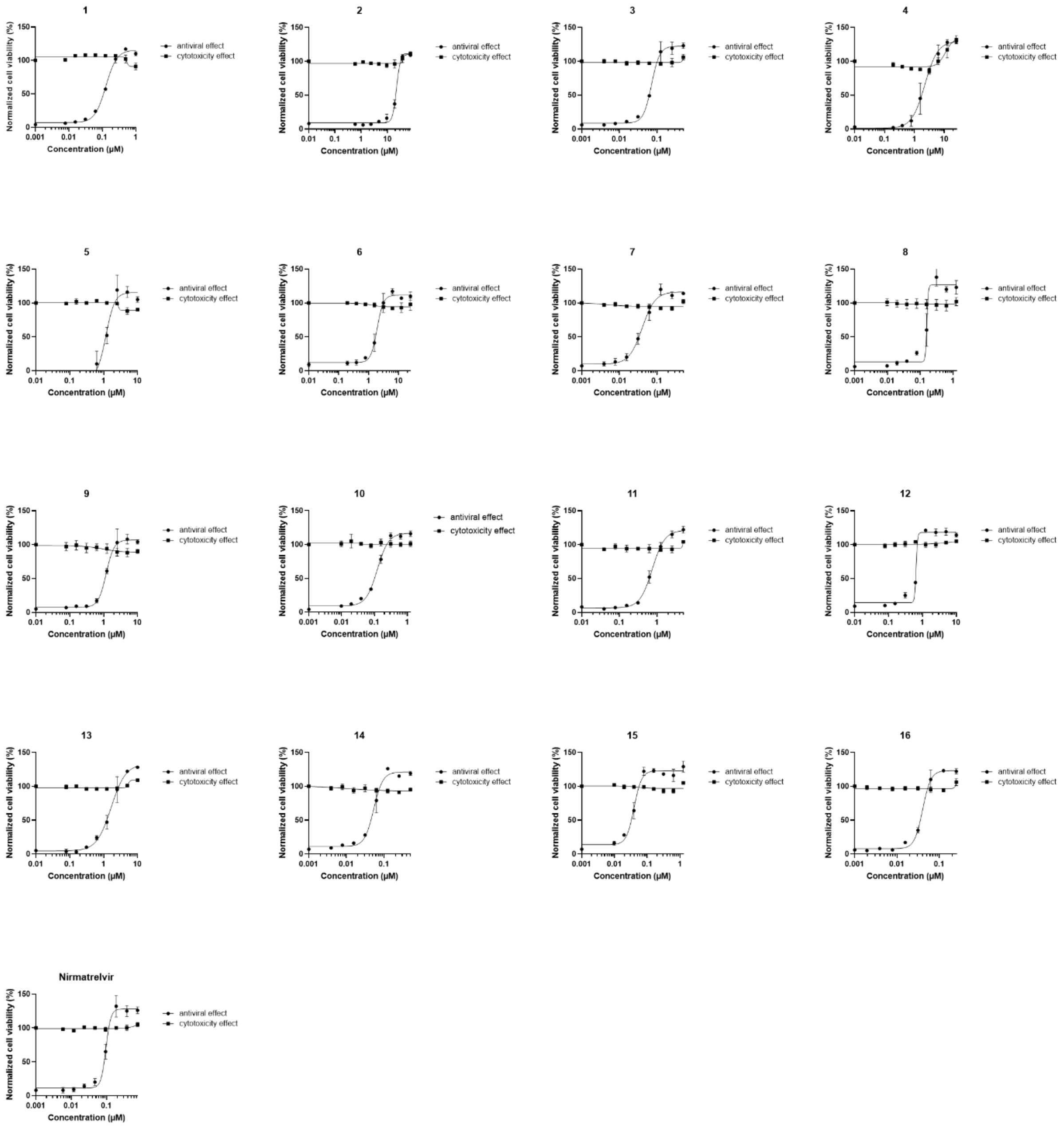

Figure S1. Results of the SARS-CoV-2 CPE reduction assays with cytotoxicity data generated in parallel.

Table S2. Biochemical mouse CTSL, mouse CTSB and hamster CTSL activity data (N.D. = No data).

Compound	pIC_{50} (mouse CTSL)	pIC_{50} (mouse CTSB)	pIC_{50} (hamster CTSL)
1	7.08 ± 0.11	N.D.	8.34 ± 0.09
2	4.87	N.D.	N.D.
3	7.12	N.D.	N.D.
4	4.98	N.D.	N.D.
5	6.65	7.91	N.D.
6	7.01	N.D.	N.D.
7	4.45	5.73	5.07 ± 0.10
8	6.54 ± 0.14	N.D.	7.92 ± 0.18
9	6.80	N.D.	N.D.
10	6.03 ± 0.16	N.D.	N.D.
11	6.50	N.D.	N.D.
12	7.66	> 8.16	N.D.
13	7.63	> 8.16	N.D.
14	< 4.30	5.30	4.67 ± 0.33
15	6.22	N.D.	N.D.
16	6.26	N.D.	N.D.
Nirmatrelvir	< 4.30	5.88	N.D.

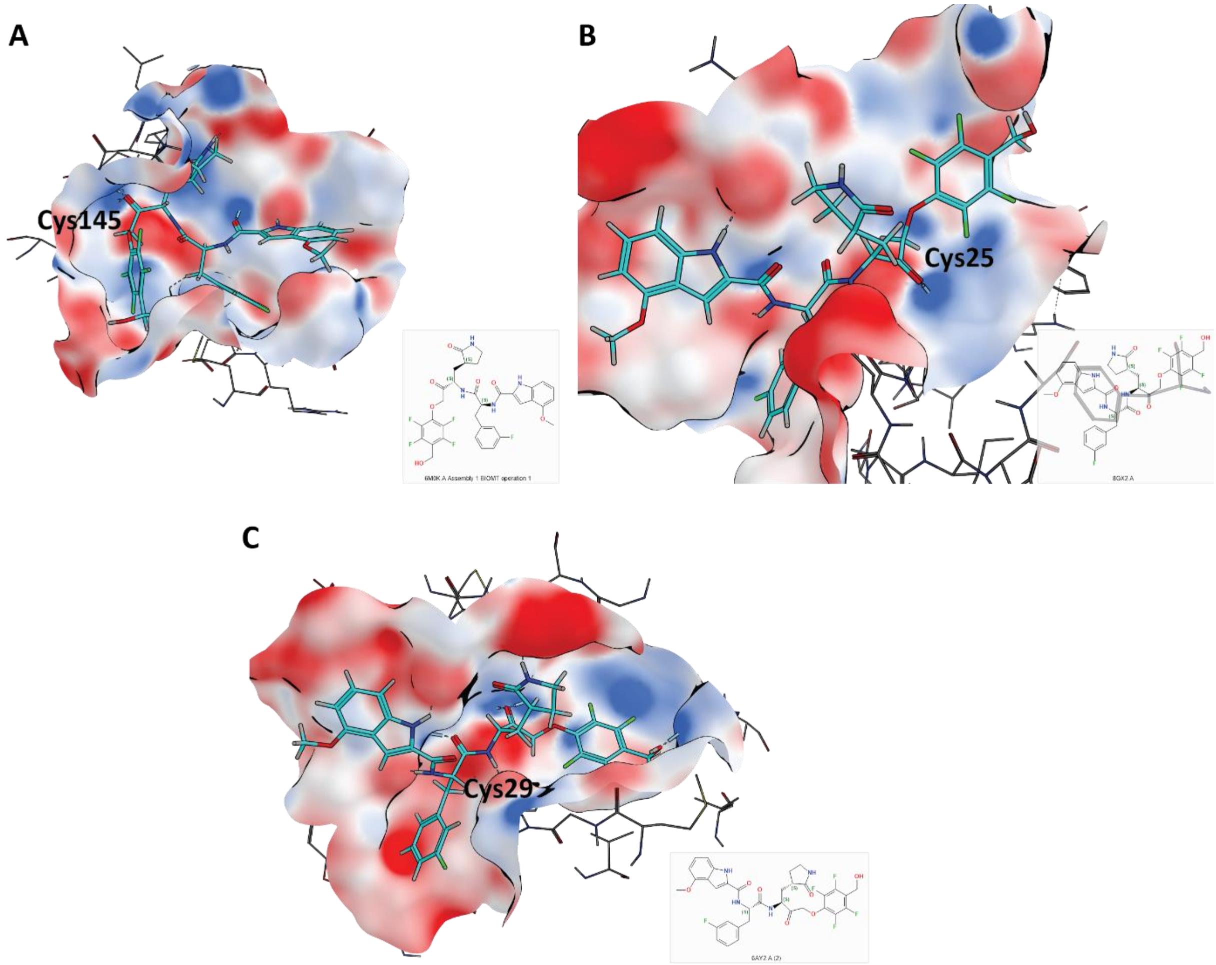

Figure S2. Predicted Michaelis complexes of **1** with **A)** SARS-CoV-2 M^{pro}, **B)** hCTSL and **C)** hCTSB. Molecular surface is coloured by Poisson-Boltzman electrostatics with the catalytic Cys highlighted for every complex.

Table S3. Inhibition (%) at 10 µM testing concentration of selected compounds in a panel of proteases (N.D. = No data).

Compound	Calpain-1	Caspase 2	Cathepsin D	Neutrophil Elastase 2	Thrombin	Trypsin
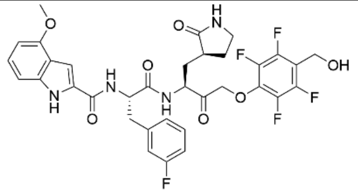 **1**	3	11	8	2	0.2 ± 1.1	-3.4 ± 9.0
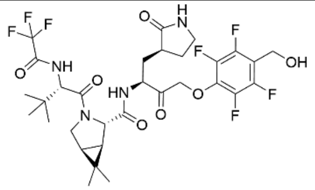 **7**	N.D.	N.D.	N.D.	N.D.	0.4 ± 0.9	9.9 ± 6.6
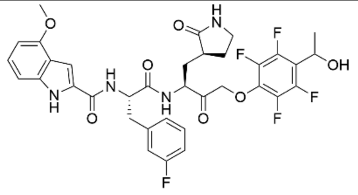 **8**	5	20	9	2	-0.2 ± 0.6	7.5 ± 4.9
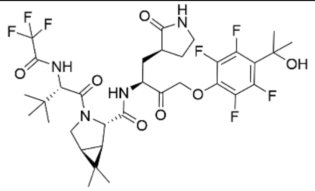 **14**	-7	7	-23	4	4.1 ± 1.5	10.2 ± 3.3

Table S4. Mouse, hamster and human liver microsomal stability of selected compounds (N.D. = No data).

Compound	Mouse microsomes	Hamster microsomes	Human microsomes
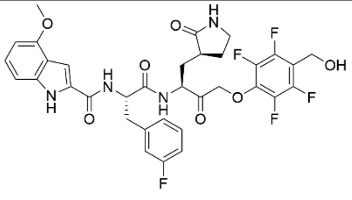 **1**	CL = 586 µL/min/mg protein<br>$t_{1/2}$ = 1.18 min<br>0.00% remaining at 60 min	CL = 2373 µL/min/mg protein<br>$t_{1/2}$ = 0.58 min<br>0.00% remaining at 60 min	CL = 350 µL/min/mg protein<br>$t_{1/2}$ = 4.04 min<br>0.00% remaining at 60 min
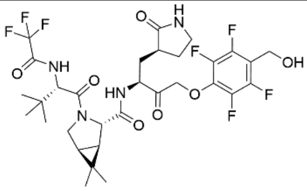 **7**	N.D.	CL = 803 µL/min/mg protein<br>$t_{1/2}$ = 1.73 min<br>0.15% remaining at 60 min	N.D.
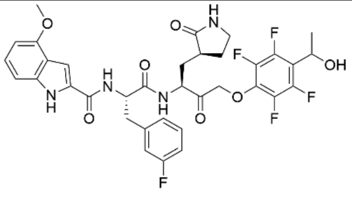 **8**	CL = 1100 µL/min/mg protein<br>$t_{1/2}$ = 1.28 min<br>0.00% remaining at 60 min	CL = 1287 µL/min/mg protein<br>$t_{1/2}$ = 1.08 min<br>0.00% remaining at 60 min	N.D.
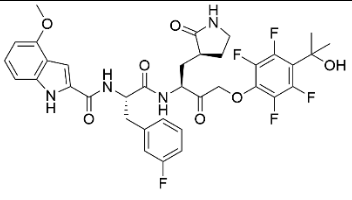 **10**	CL = 780 µL/min/mg protein<br>$t_{1/2}$ = 1.90 min<br>0.00% remaining at 60 min	CL = 500 µL/min/mg protein<br>$t_{1/2}$ = 2.77 min<br>0.00% remaining at 60 min	N.D.
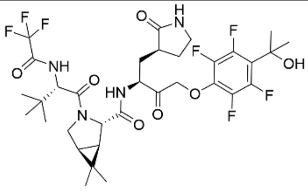 **14**	N.D.	CL = 377 µL/min/mg protein<br>$t_{1/2}$ = 1.73 min<br>0.10% remaining at 60 min	CL = 40 µL/min/mg protein<br>$t_{1/2}$ = 35.03 min<br>30.18% remaining at 60 min

Table S5. Mouse and human hepatocyte stability with and without co-administration of ritonavir (ratio 4:1) of selected compounds (N.D. = No data).

**Compound**	**Mouse hepatocyte stability**		**Mouse hepatocyte stability + ritonavir 4:1**		**Human hepatocyte stability**		**Human hepatocyte stability + ritonavir 4:1**	
	**CL_{int} [µL/min/ 10^6 cells]**	**$t_{1/2}$ [min]**	**CL_{int} [µL/min/ 10^6 cells]**	**$t_{1/2}$ [min]**	**CL_{int} [µL/min/ 10^6 cells]**	**$t_{1/2}$ [min]**	**CL_{int} [µL/min/ 10^6 cells]**	**$t_{1/2}$ [min]**
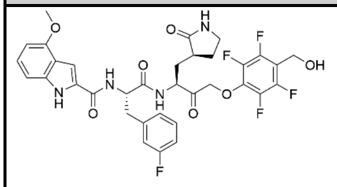 **1**	185.8	9.3	N.D.	N.D.	12.5	55.5	6.9	100.3
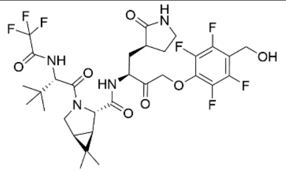 **7**	121.2	14.3	21.8	79.5	N.D.	N.D.	N.D.	N.D.
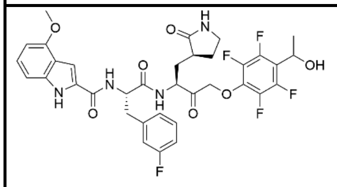 **8**	64.5	26.9	N.D.	N.D.	N.D.	N.D.	N.D.	N.D.
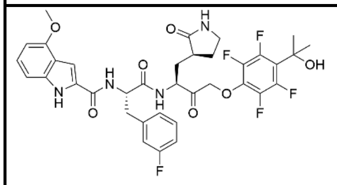 **10**	47.6	36.4	N.D.	N.D.	N.D.	N.D.	N.D.	N.D.
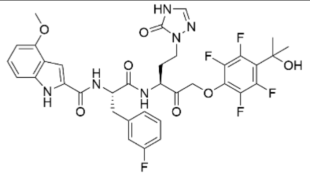 **12**	76.8	22.6	37.1	46.7	N.D.	N.D.	N.D.	N.D.
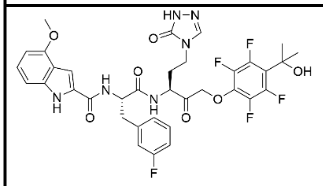 **13**	78.8	22.0	49.3	35.2	N.D.	N.D.	N.D.	N.D.
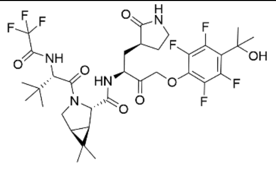 **14**	112.1	15.5	26.7	64.9	6.6	105.3	0.65	> 120

Table S6. Peak areas (%) of **1** (P), P2/P3 oxidation (M1), P1 oxidation (M2) and P1 ketone formation (M3) obtained by LCMS analysis of a mouse liver microsomal stability assay.

Time Points (min)	P	M1	M2	M3
0	100	0	0	0
5	86.9	2.2	8.3	2.5
15	67.9	4.2	17.7	10.2
30	48.7	9.5	22.5	19.2
45	35.8	17.7	23.3	23.2
60	32.6	18.7	23.8	24.9

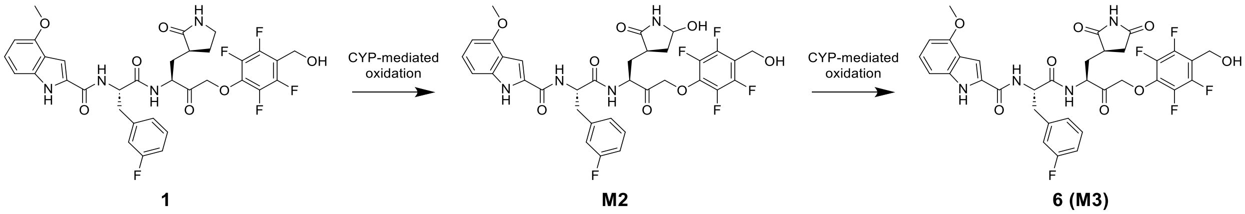

Scheme S1. Proposed CYP-mediated metabolism of **1** towards succinimide **6** (M3).

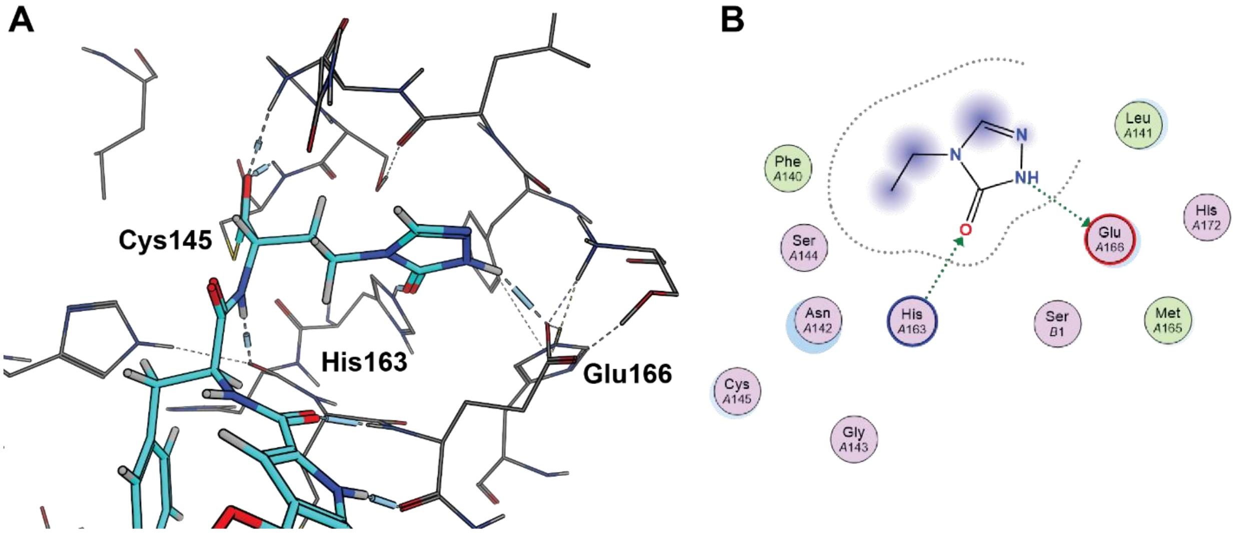

Figure S3. Predicted binding mode of **13** after covalent reaction with SARS-CoV-2 M^{pro}. **A)** 3D-overview of M^{pro} S1-pocket with key-residues highlighted and **13** in cyan. **B)** 2D-ligand interactions of the P1 triazolone sidechain. Figures were prepared with MOE[8] using the supplementary method on page S7.

Table S7. Hamster hepatocyte stability with co-administration of ritonavir (ratios 2:1 and 1:1) of selected compounds.

Compound	Compound/ritonavir 2:1	Compound/ritonavir 1:1
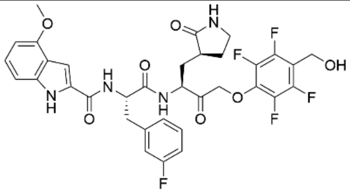 **1**	CL_{int} = 48.13 µL/min/10^6 cells<br>$t_{1/2}$ = 14.40 min<br>0.56% remaining at 120 min	CL_{int} = 31.40 µL/min/10^6 cells<br>$t_{1/2}$ = 22.08 min<br>2.51% remaining at 120 min
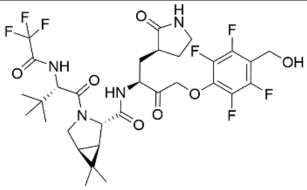 **7**	CL_{int} = 27.36 µL/min/10^6 cells<br>$t_{1/2}$ = 25.33 min<br>3.47% remaining at 120 min	CL_{int} = 12.48 µL/min/10^6 cells<br>$t_{1/2}$ = 55.55 min<br>20.82% remaining at 120 min
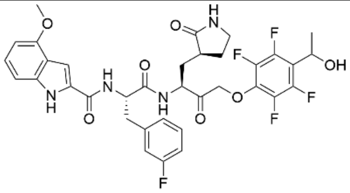 **8**	CL_{int} = 31.70 µL/min/10^6 cells<br>$t_{1/2}$ = 21.87 min<br>2.78% remaining at 120 min	CL_{int} = 22.57 µL/min/10^6 cells<br>$t_{1/2}$ = 30.71 min<br>7.05% remaining at 120 min
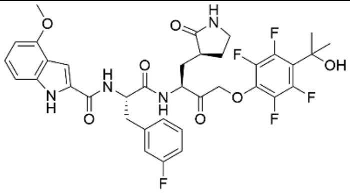 **10**	CL_{int} = 30.73 µL/min/10^6 cells<br>$t_{1/2}$ = 22.56 min<br>2.17% remaining at 120 min	CL_{int} = 16.42 µL/min/10^6 cells<br>$t_{1/2}$ = 42.21 min<br>11.62% remaining at 120 min
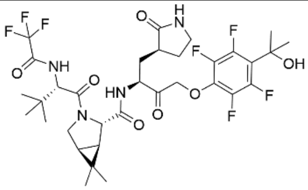 **14**	CL_{int} = 25.44 µL/min/10^6 cells<br>$t_{1/2}$ = 27.24 min<br>4.42% remaining at 120 min	CL_{int} = 9.30 µL/min/10^6 cells<br>$t_{1/2}$ = 74.50 min<br>28.77% remaining at 120 min

Table S8. In vivo PK parameters of selected compounds pretreated orally with ritonavir (100 mg/kg) in female Syrian golden hamsters following intravenous (5 mg/kg), intraperitoneal (100 mg/kg), subcutaneous (100 mg/kg) and oral (200 mg/kg) administration (n=3).

	**1**				**7**			
	**I.V.**	**I.P.**	**S.C.**	**P.O.**	**I.V.**	**I.P.**	**S.C.**	**P.O.**
**C_{max} [ng/mL]**		1874 ± 291	1206 ± 196	650 ± 338		4552 ± 303	2575 ± 500	597 ± 56
**t_{max} [h]**		4.00 ± 0.00	1.00 ± 0.00	1.00 ± 0.00		0.42 ± 0.14	1.00 ± 0.00	0.33 ± 0.14
**$t_{1/2}$ [h]**	2.77 ± 0.76	8.23 ± 1.68	11.1 ± 2.18	24.8 ± 4.21	3.54 ± 0.68	4.68 ± 0.74	3.17 ± 0.64	7.54 ± 6.40
**AUC0-inf [ng*h/mL]**	1443 ± 203	19542 ± 2955	14205 ± 4940	2511 ± 1970	6454 ± 778	9528 ± 79	12889 ± 2489	1633 ± 210
**%F**		62	40	3.0		7.3	10.0	0.6
	**8**				**14**			
	**I.V.**	**I.P.**	**S.C.**	**P.O.**	**I.V.**	**I.P.**	**S.C.**	**P.O.**
**C_{max} [ng/mL]**		951 ± 221	372 ± 31	29 ± 4.5		11931 ± 1965	4096 ± 643	1581 ± 92
**t_{max} [h]**		1.00 ± 0.00	3.33 ± 1.15	1.50 ± 2.17		3.00 ± 1.73	6.00 ± 0.00	2.67 ± 1.15
**$t_{1/2}$ [h]**	2.47 ± 0.35	3.45 ± 1.18	4.11 ± 0.79	1.52	3.82 ± 0.28	5.30 ± 1.68	3.45 ± 0.13	3.20 ± 1.60
**AUC0-inf [ng*h/mL]**	3420 ± 416	6109 ± 735	3003 ± 155	31	8633 ± 129	141356 ± 21008	49595 ± 5012	20061 ± 2749
**%F**		8.2	4.3	0.0		78.2	28.6	5.4

1.3 NMR spectra

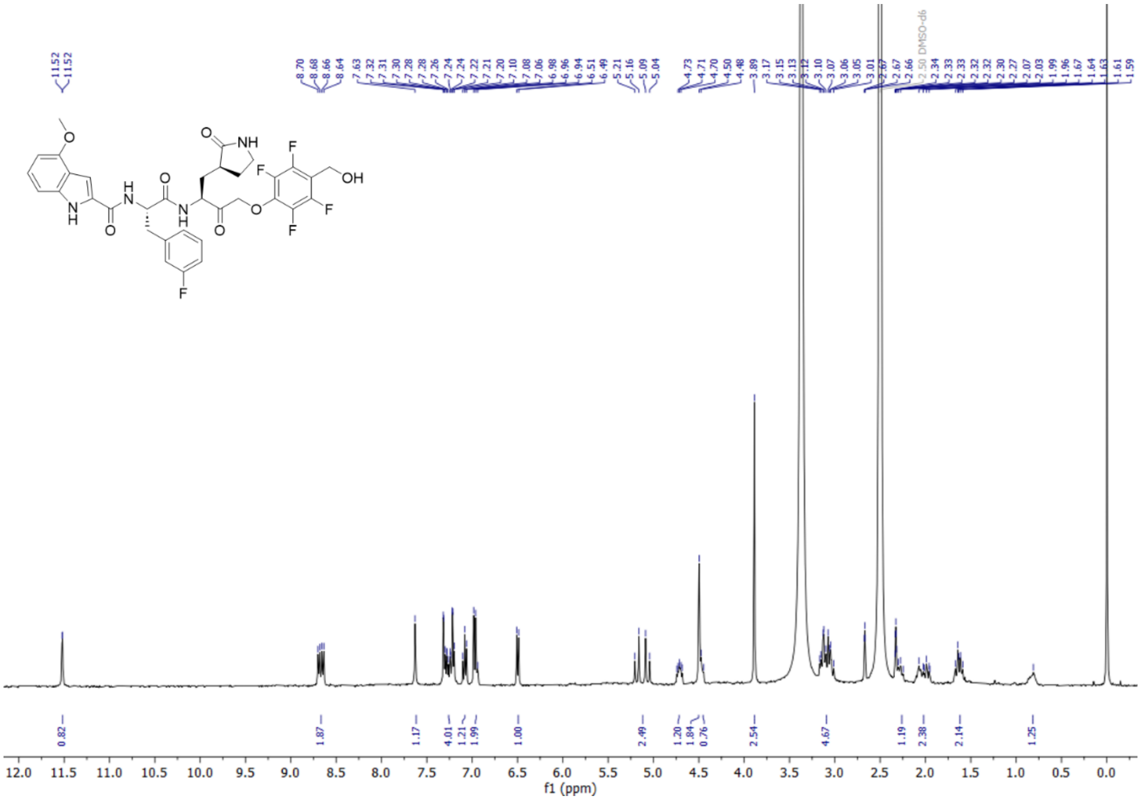

Figure S4. ^{1}H-NMR spectrum of **1**.

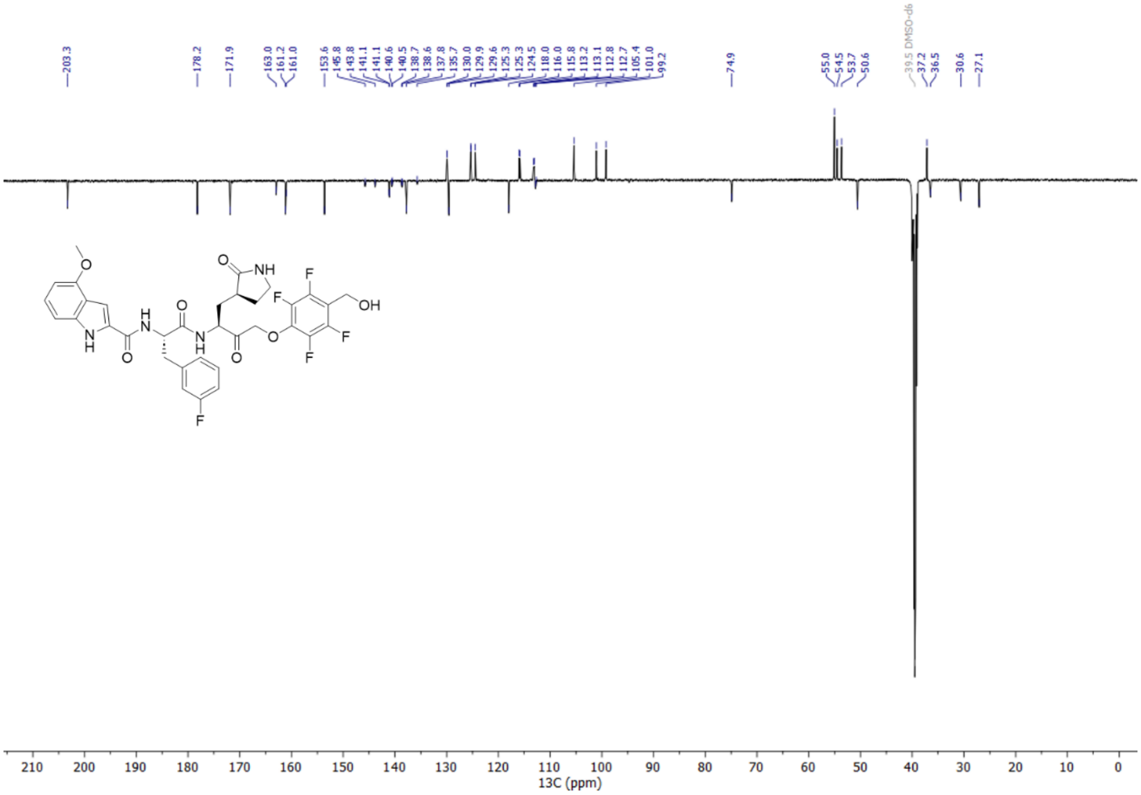

Figure S5. ^{13}C-NMR spectrum of **1**.

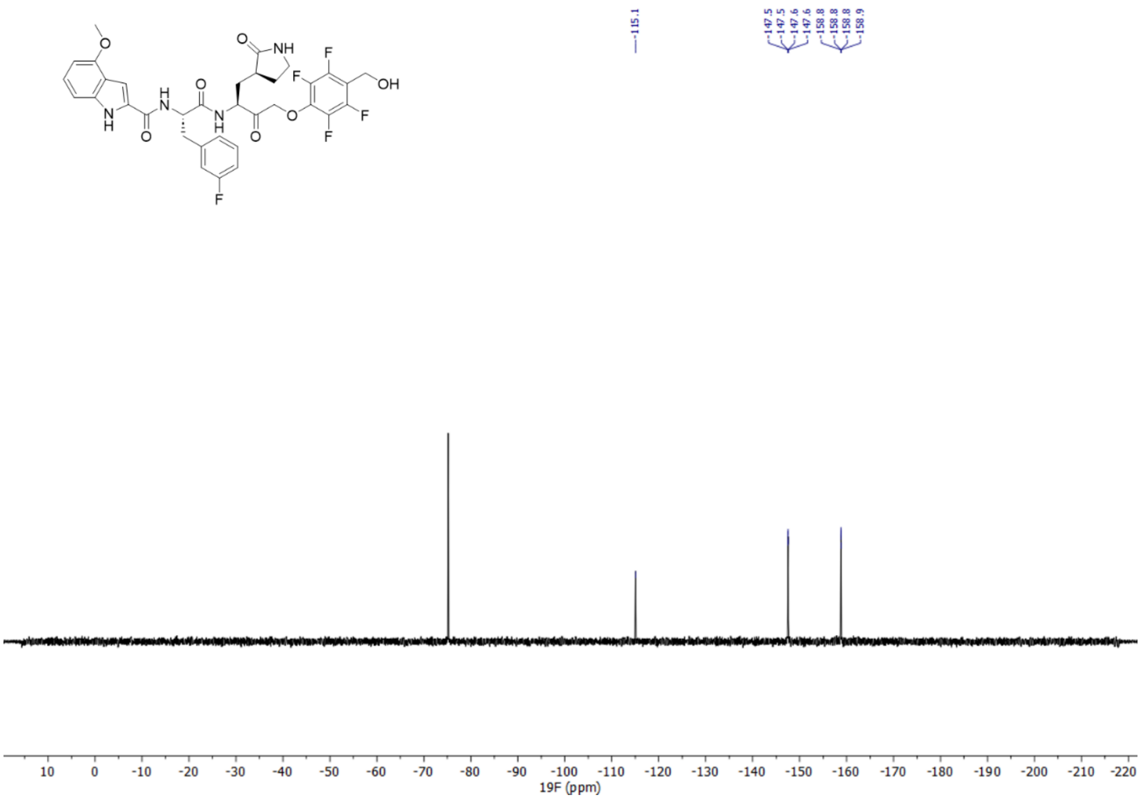

Figure S6. ^{19}F-NMR spectrum of **1**.

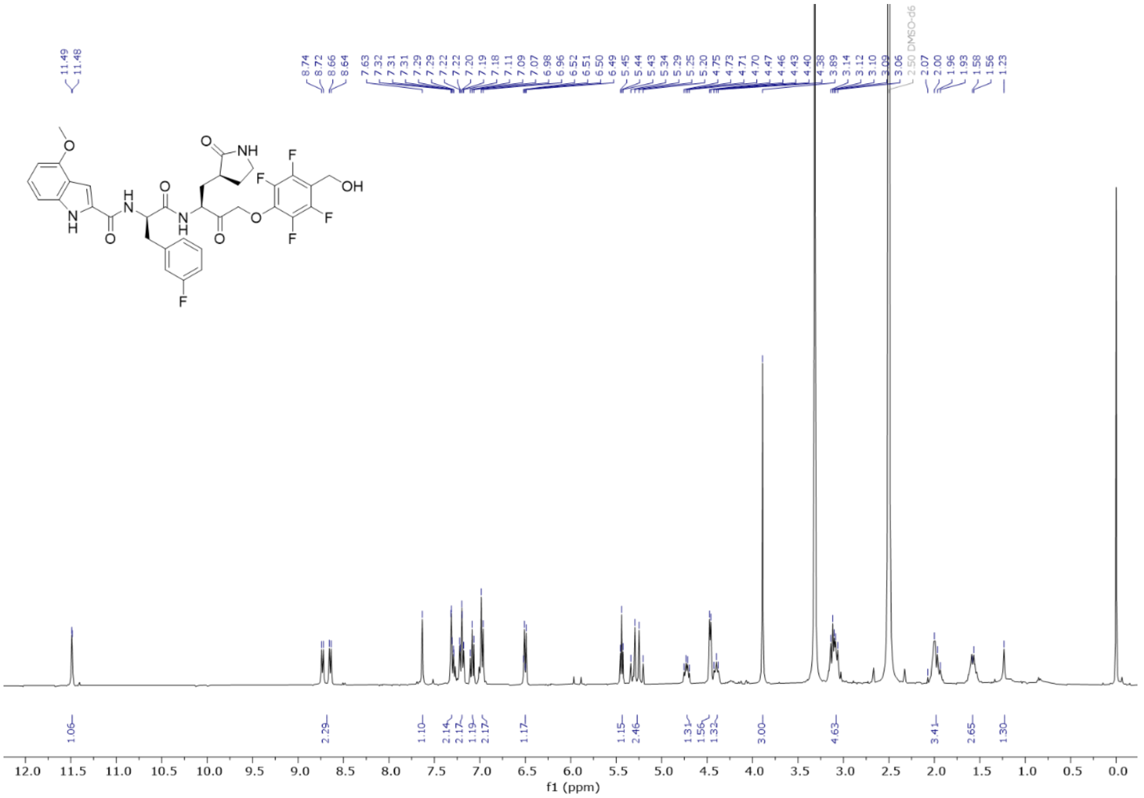

Figure S7. ^{1}H-NMR spectrum of **2**.

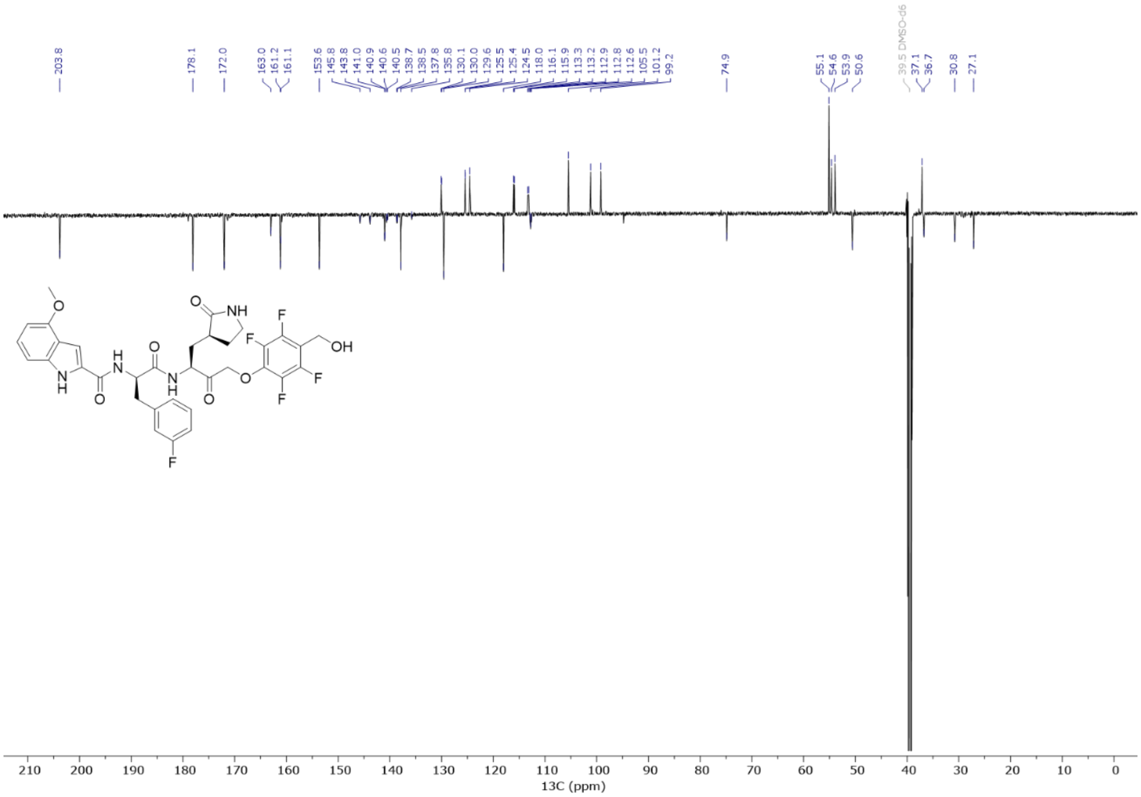

Figure S8. ^{13}C-NMR spectrum of **2**.

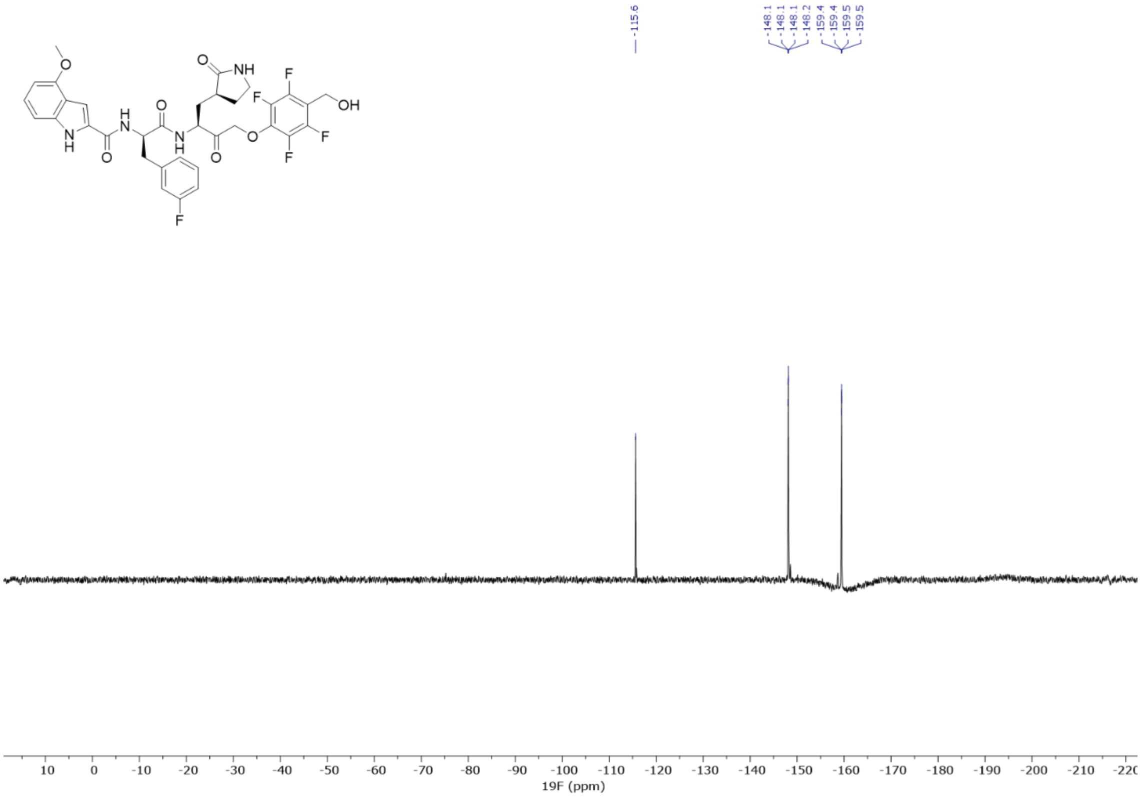

Figure S9. ^{19}F-NMR spectrum of **2**.

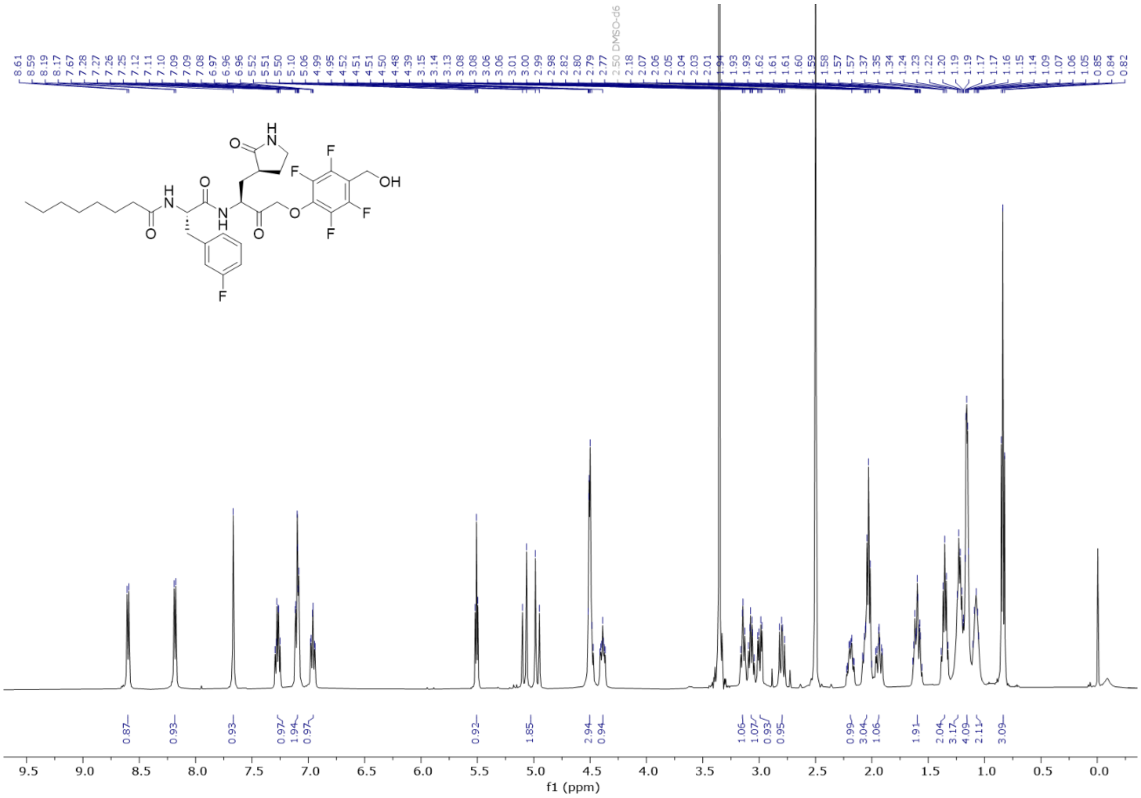

Figure S10. ^{1}H-NMR spectrum of **3**.

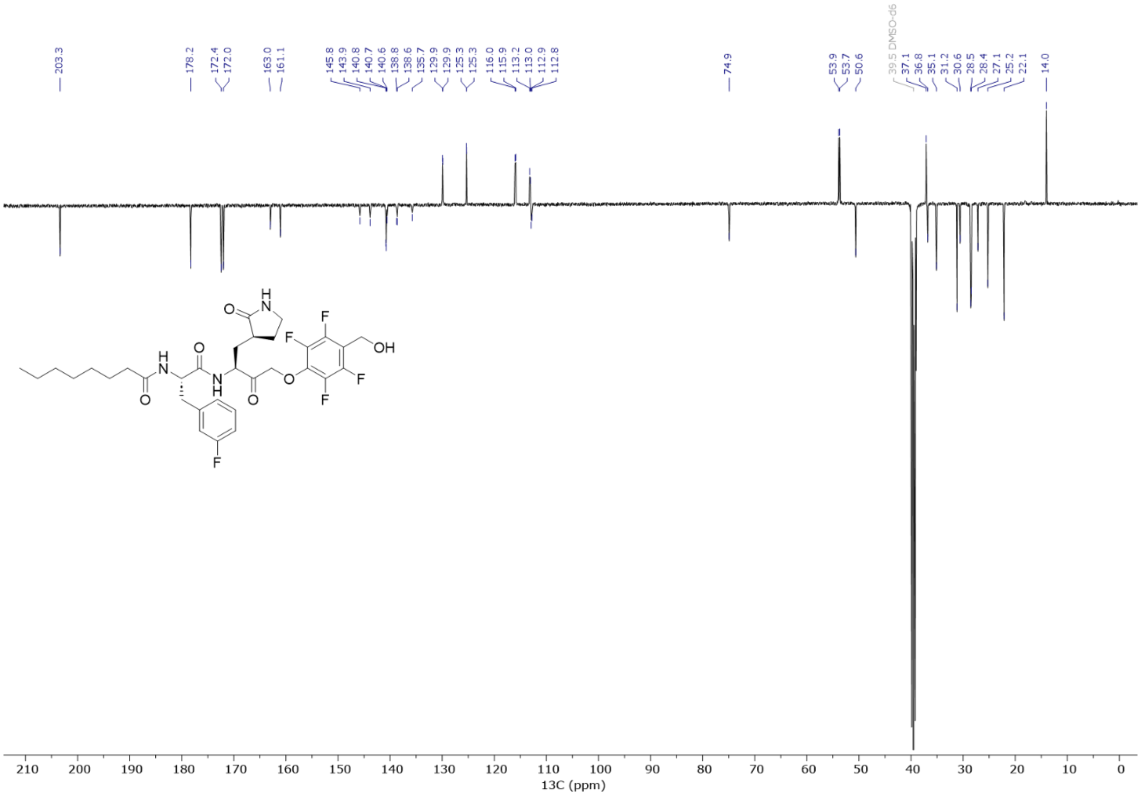

Figure S11. ^{13}C-NMR spectrum of **3**.

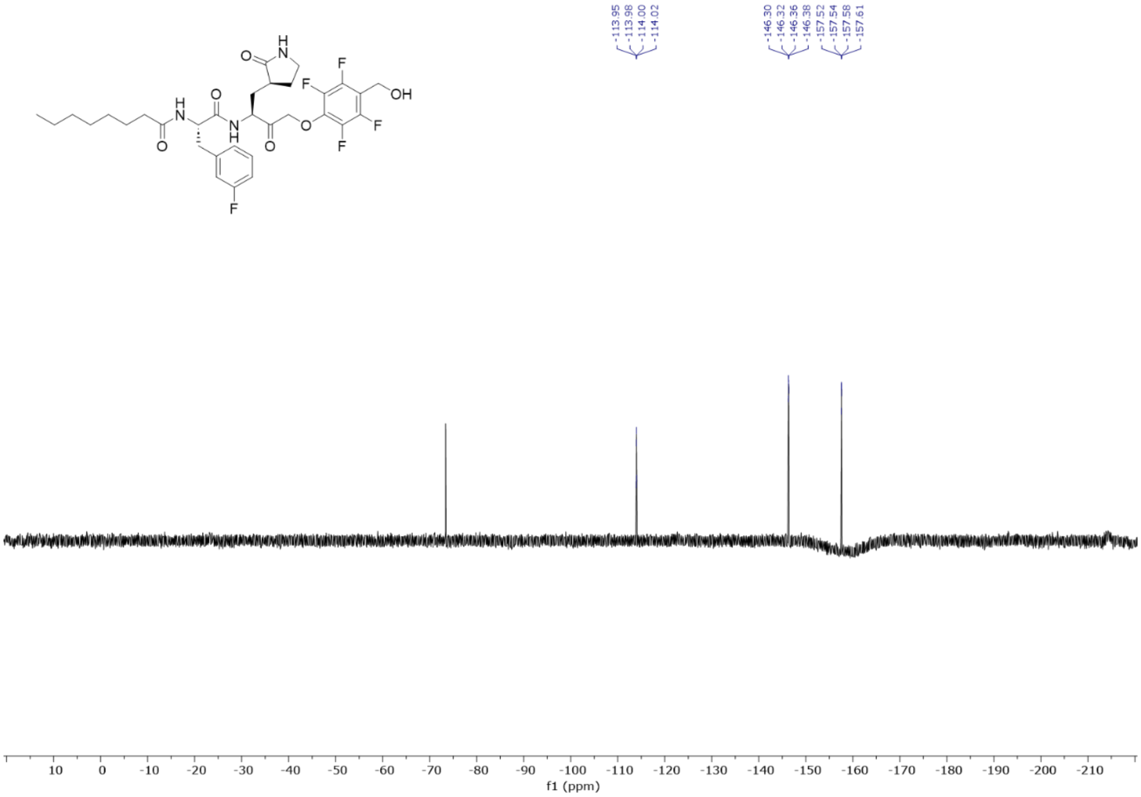

Figure S12. ^{19}F-NMR spectrum of **3**.

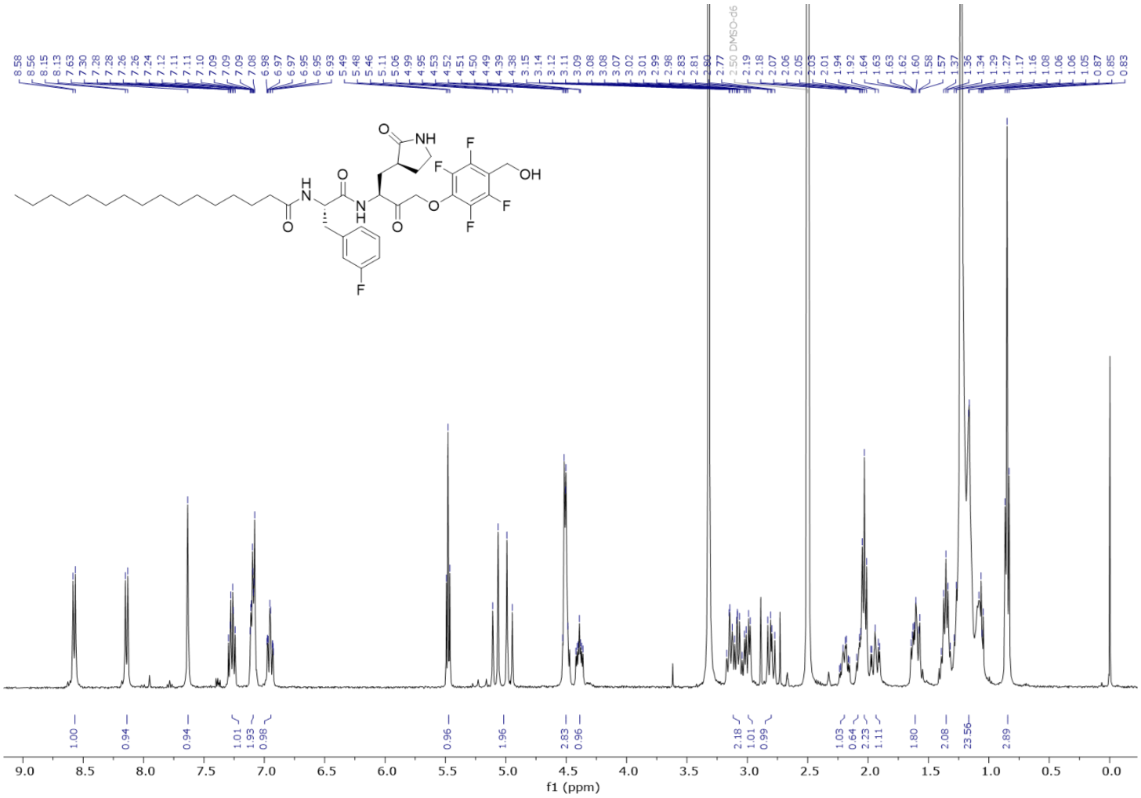

Figure S13. ^{1}H-NMR spectrum of **4**.

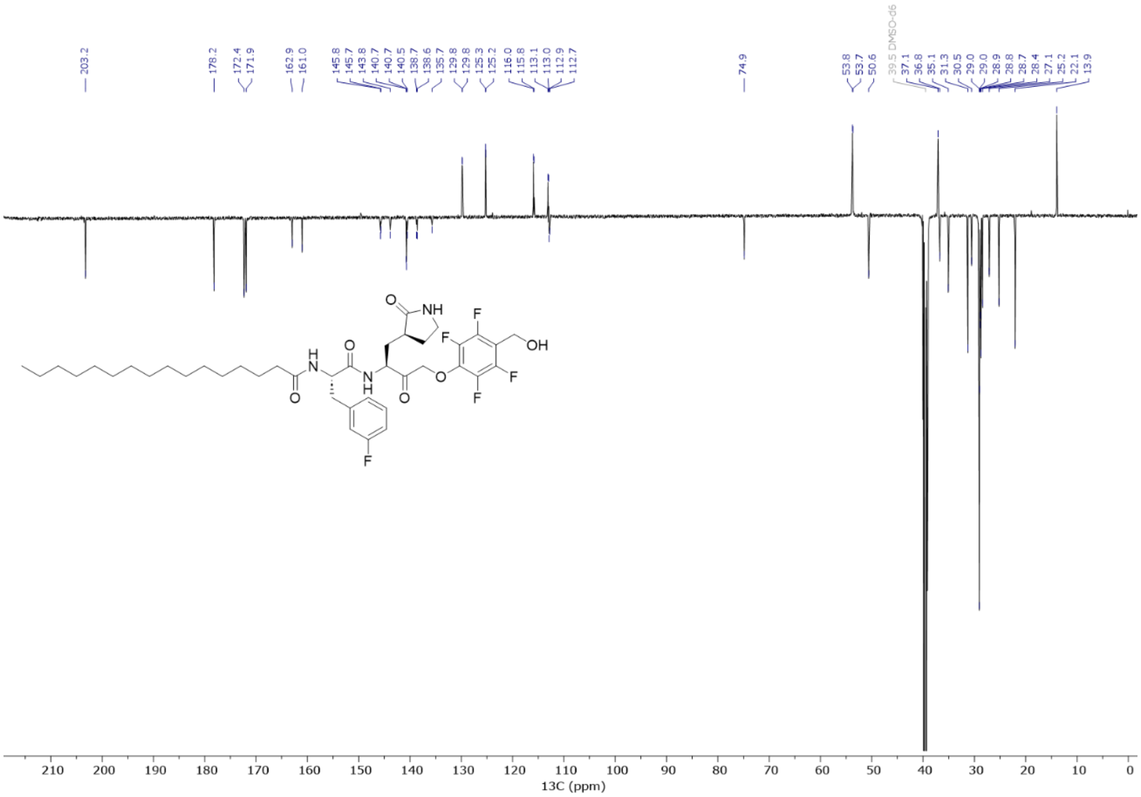

Figure S14. ^{13}C-NMR spectrum of **4**.

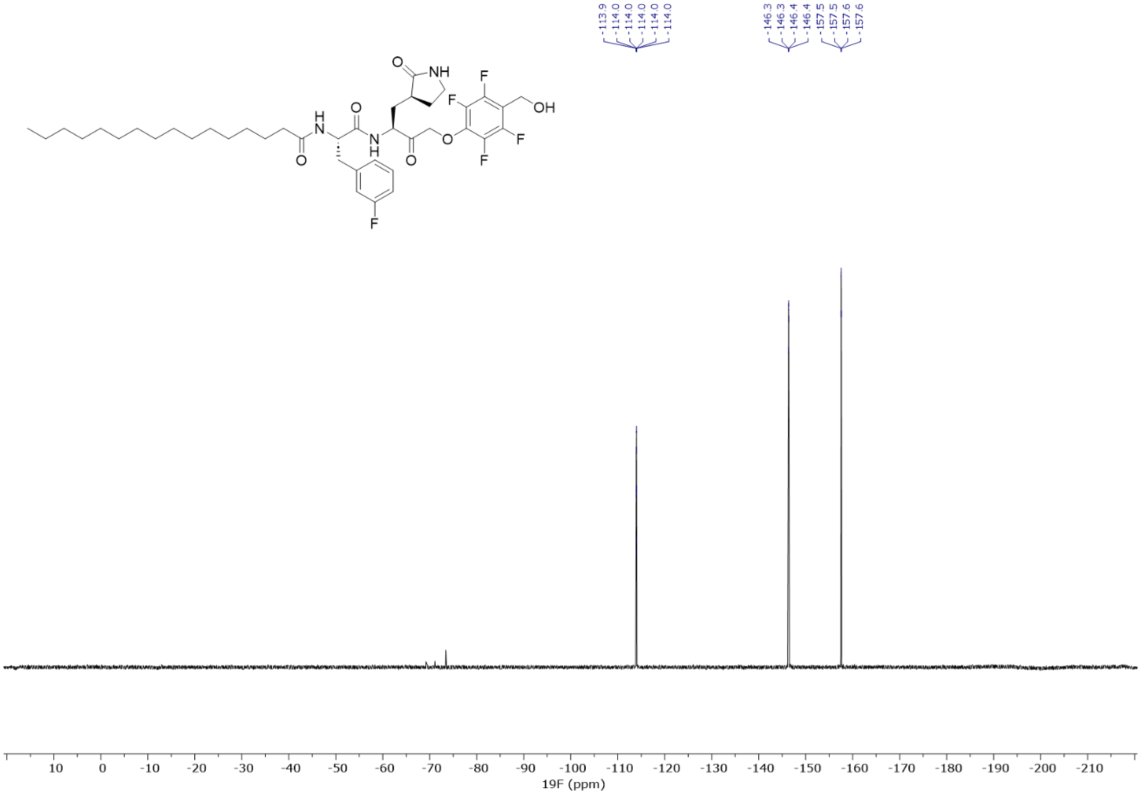

Figure S15. ^{19}F-NMR spectrum of **4**.

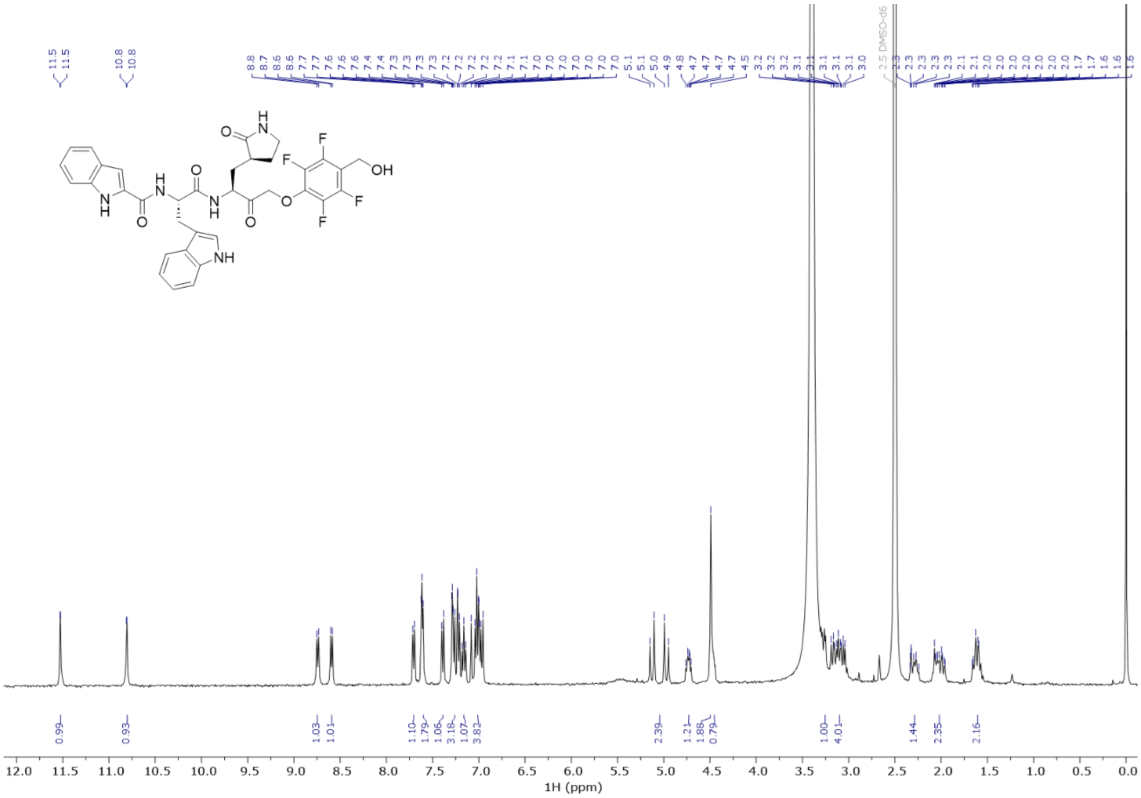

Figure S16. ^{1}H-NMR spectrum of **5**.

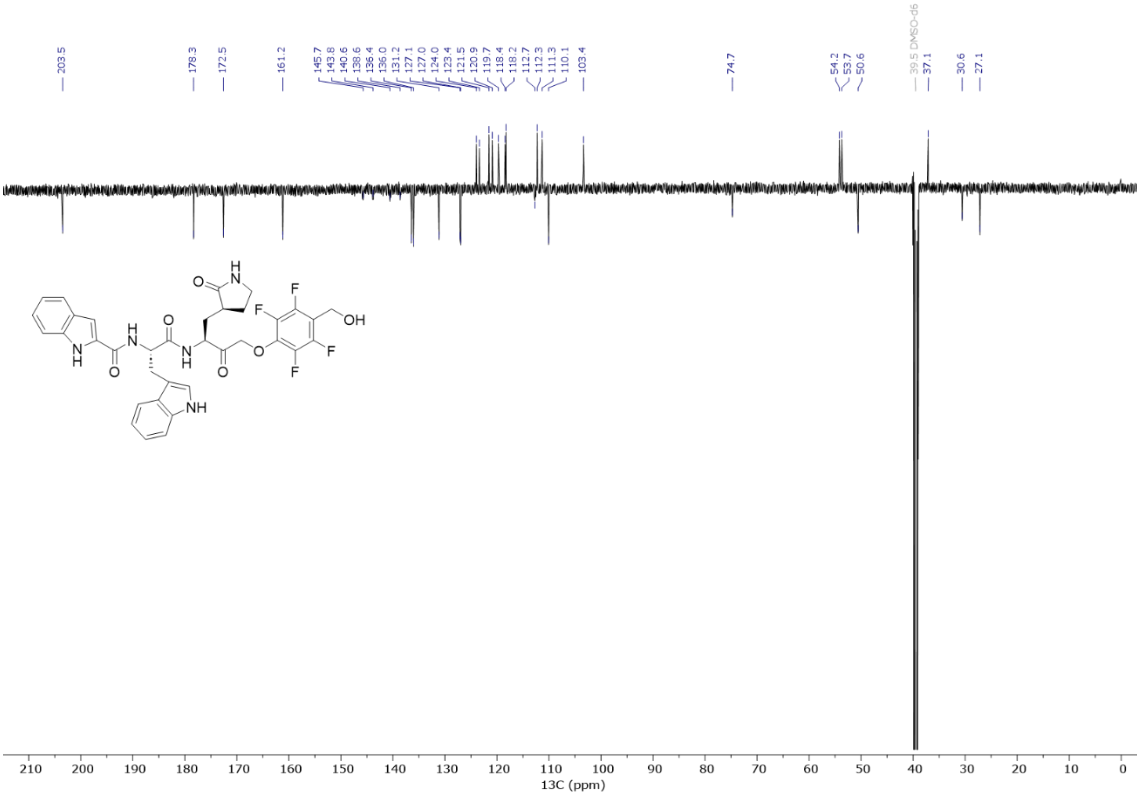

Figure S17. ^{13}C-NMR spectrum of **5**.

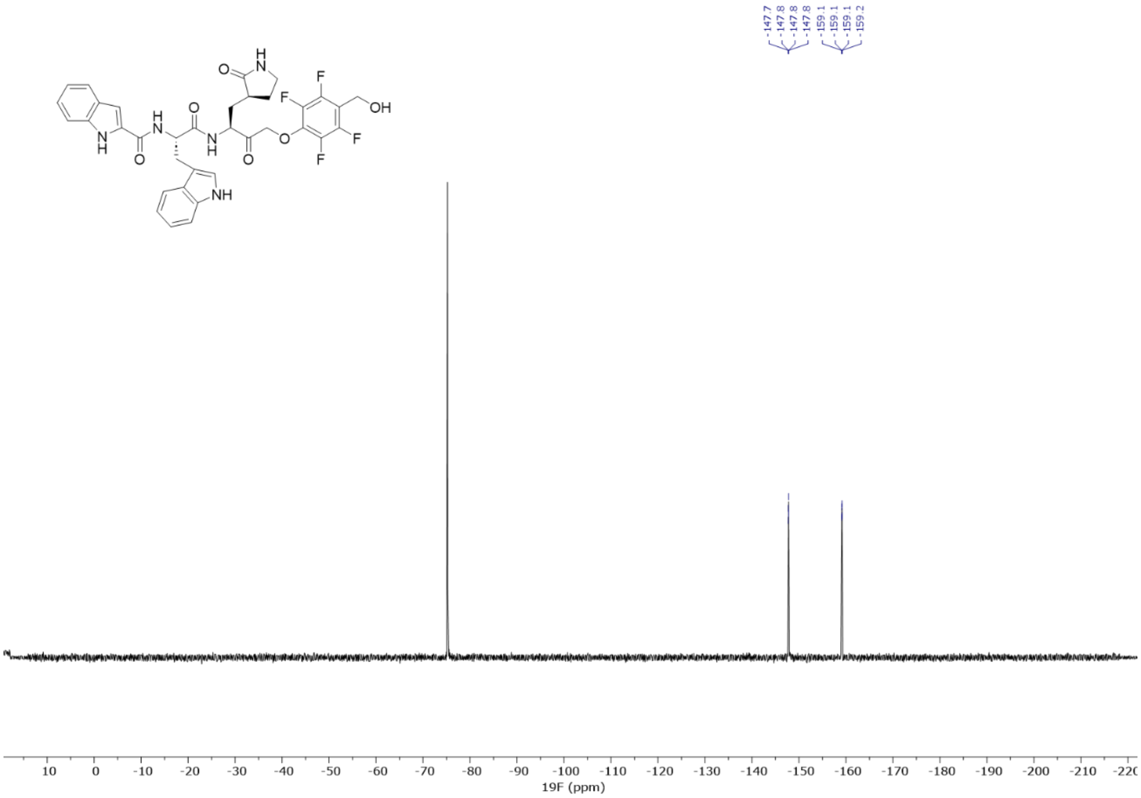

Figure S18. ^{19}F-NMR spectrum of **5**.

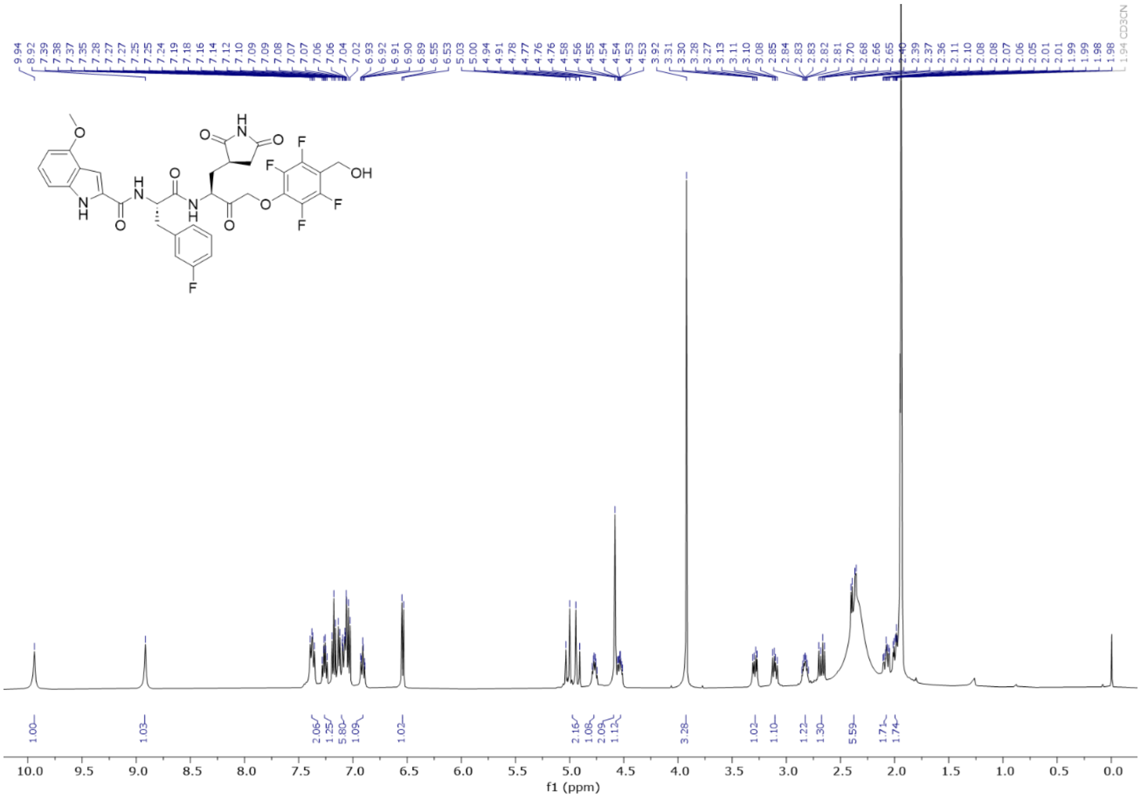

Figure S19. ^{1}H-NMR spectrum of **6**.

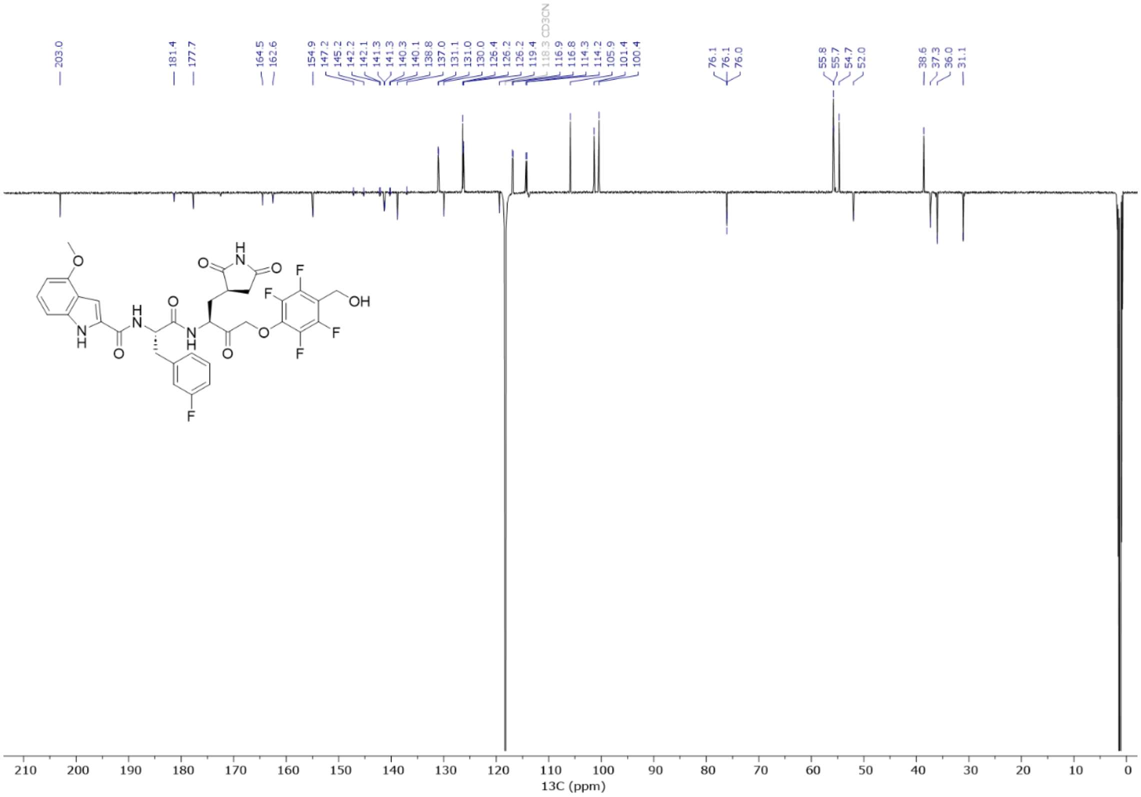

Figure S20. ^{13}C-NMR spectrum of **6**.

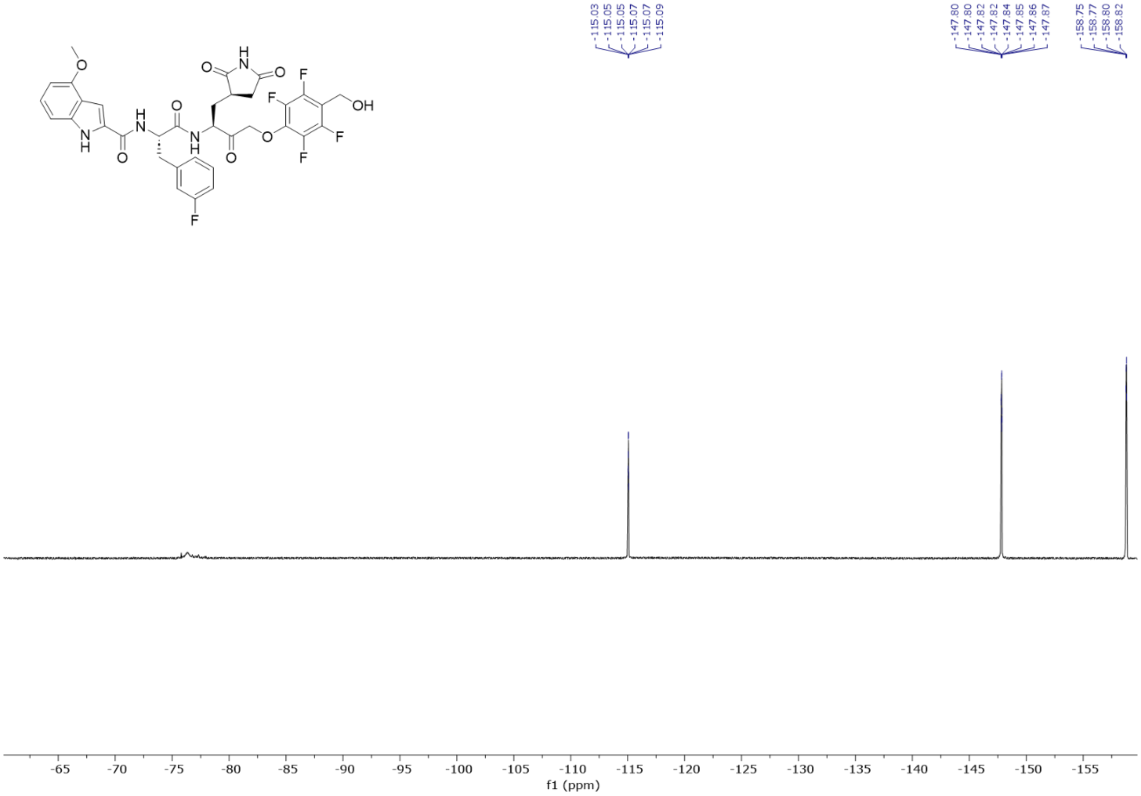

Figure S21. ^{19}F-NMR spectrum of **6**.

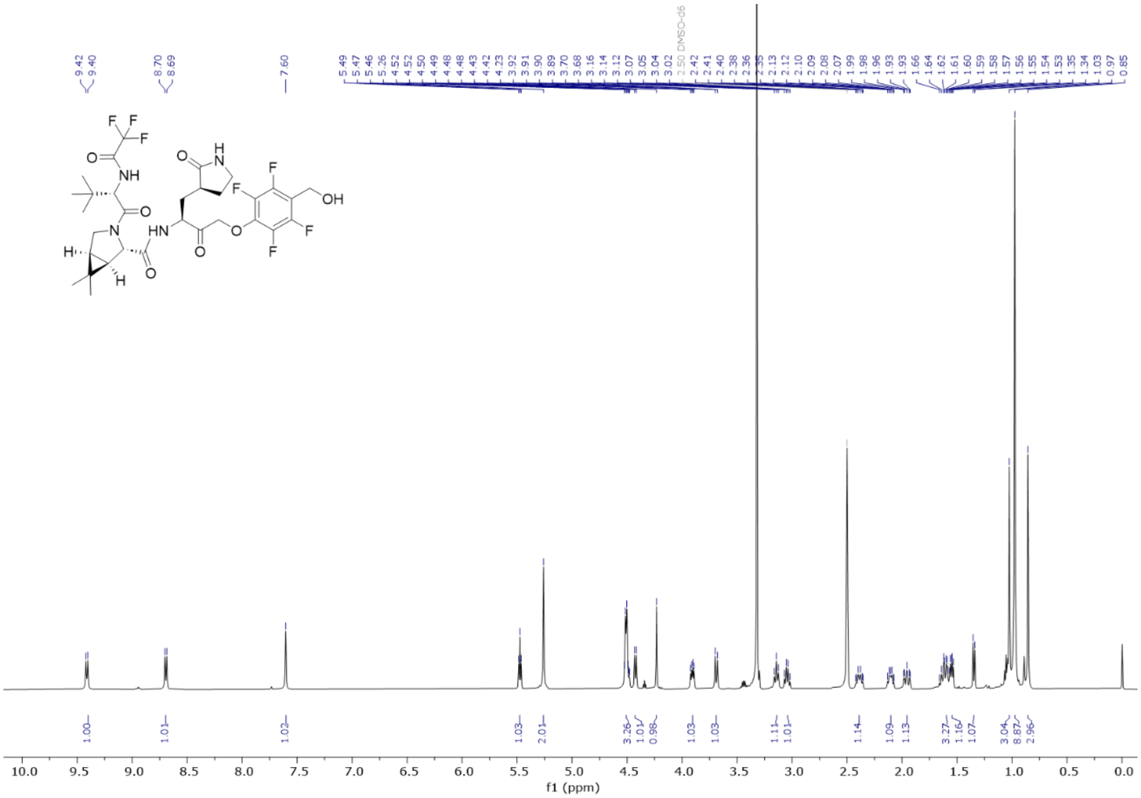

Figure S22. ^{1}H-NMR spectrum of **7**.

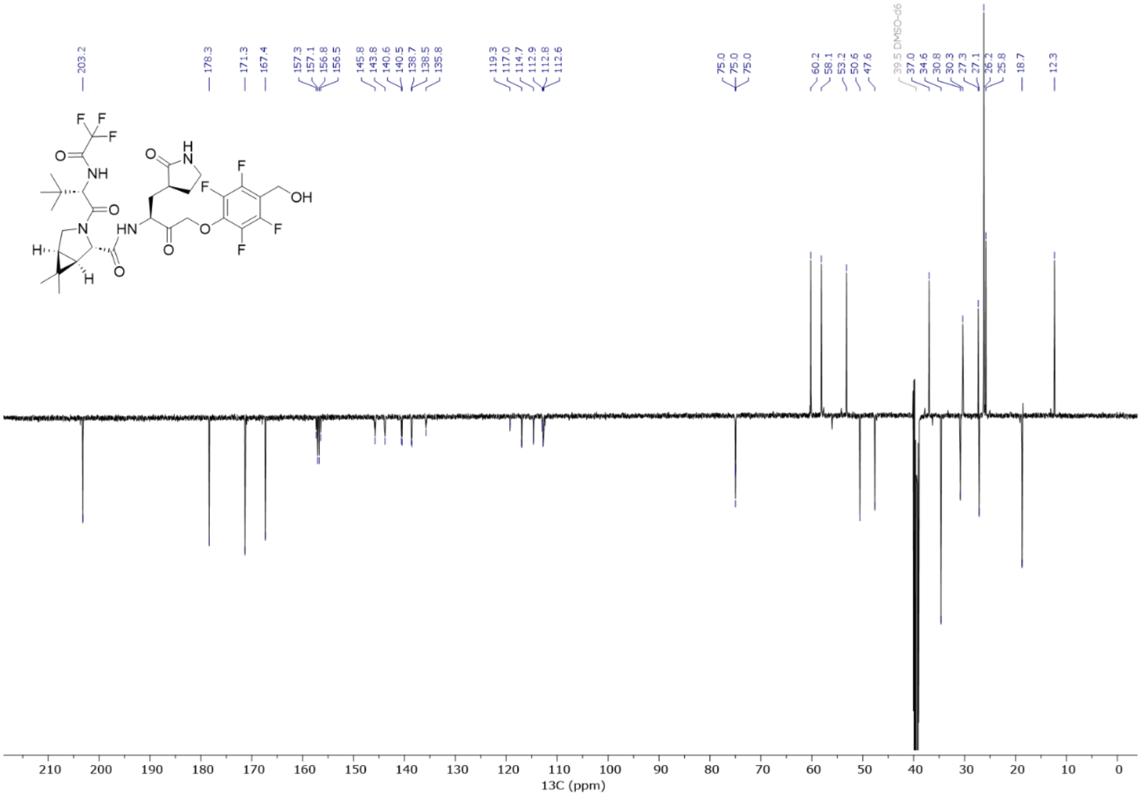

Figure S23. ^{13}C-NMR spectrum of **7**.

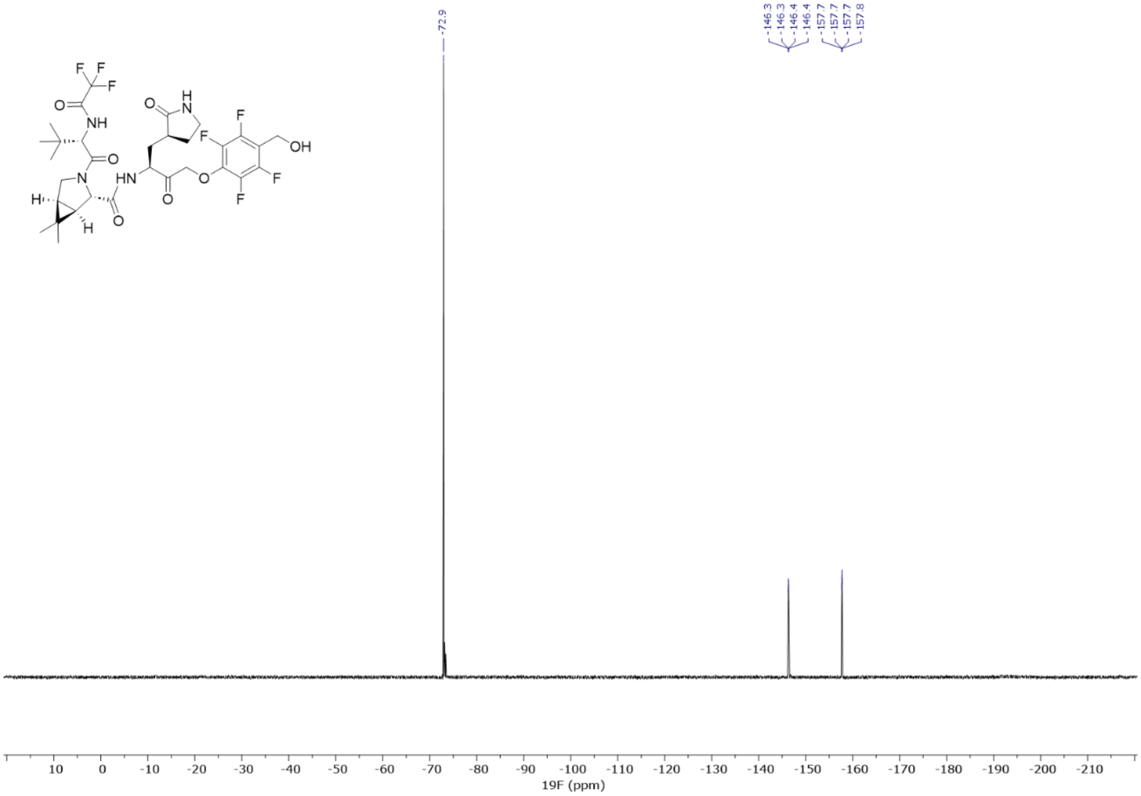

Figure S24. ^{19}F-NMR spectrum of **7**.

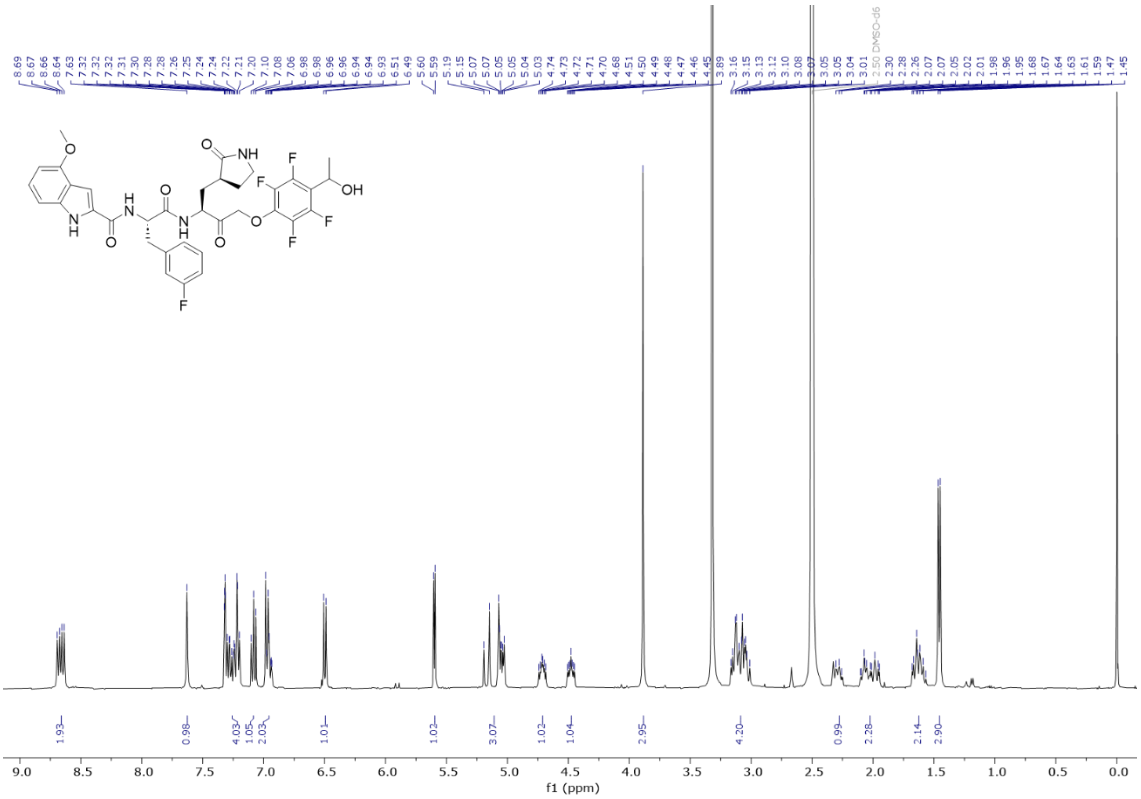

Figure S25. ^{1}H-NMR spectrum of **8**.

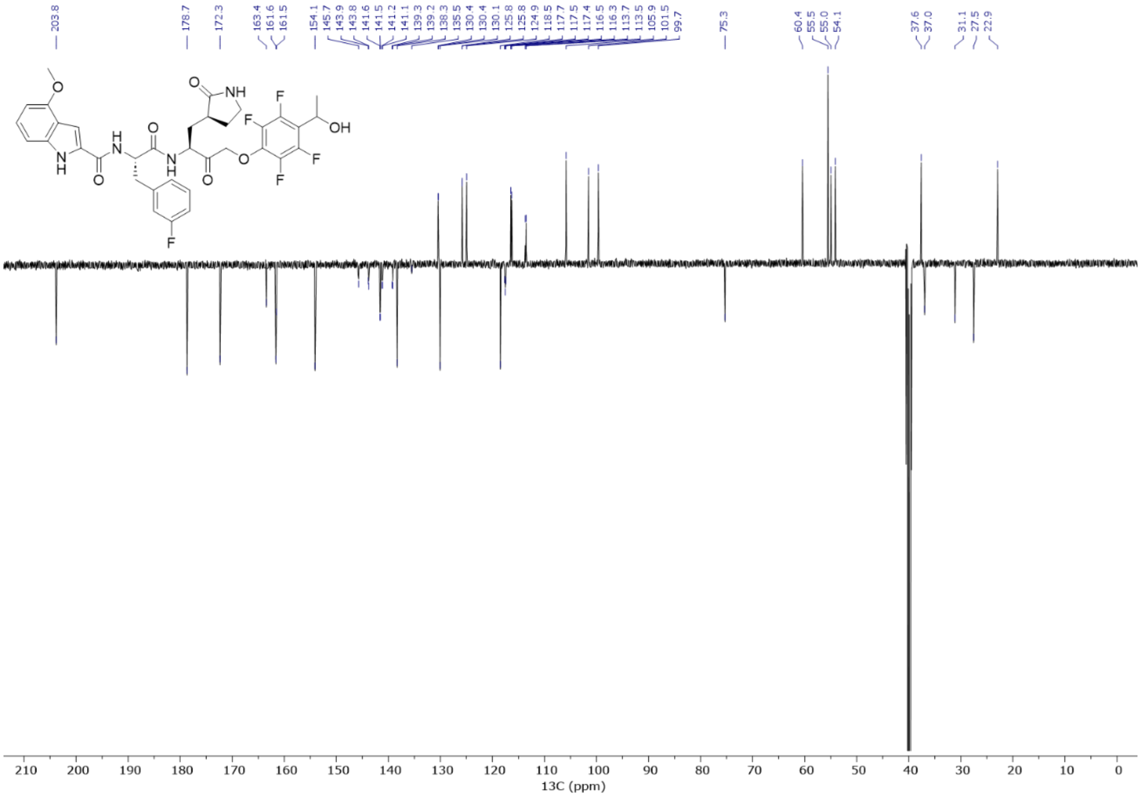

Figure S26. ^{13}C-NMR spectrum of **8**.

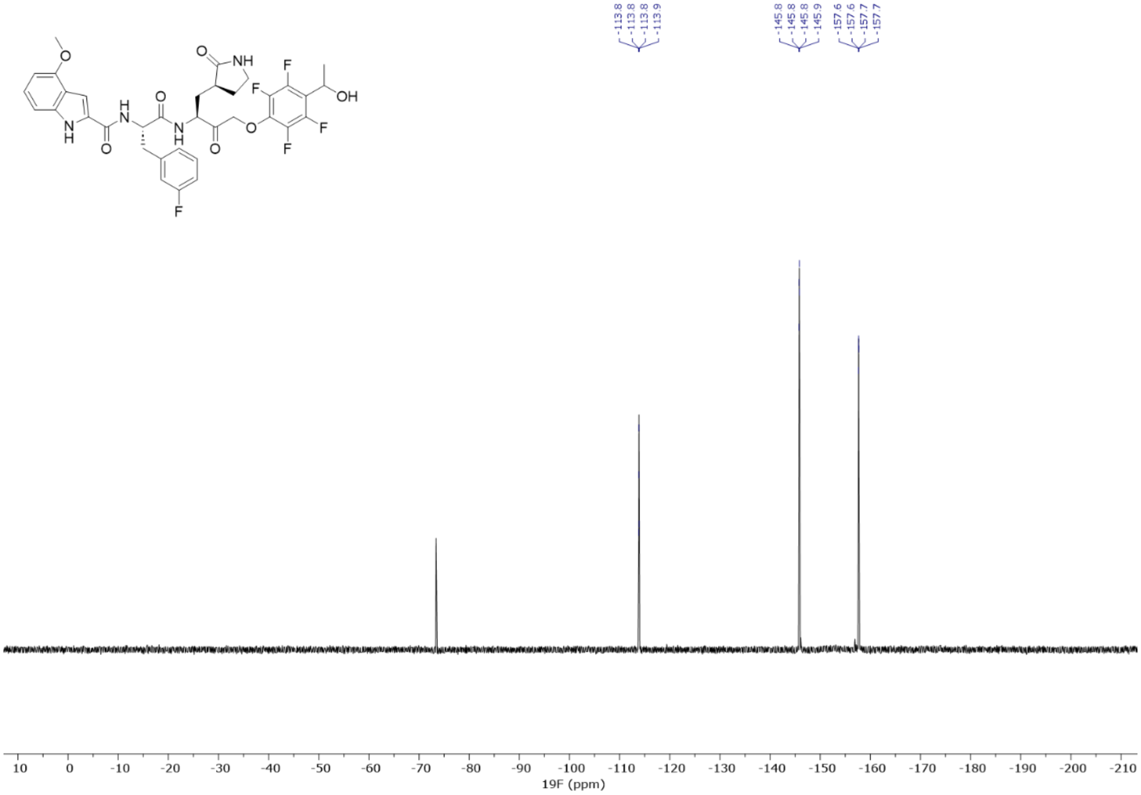

Figure S27. ^{19}F-NMR spectrum of **8**.

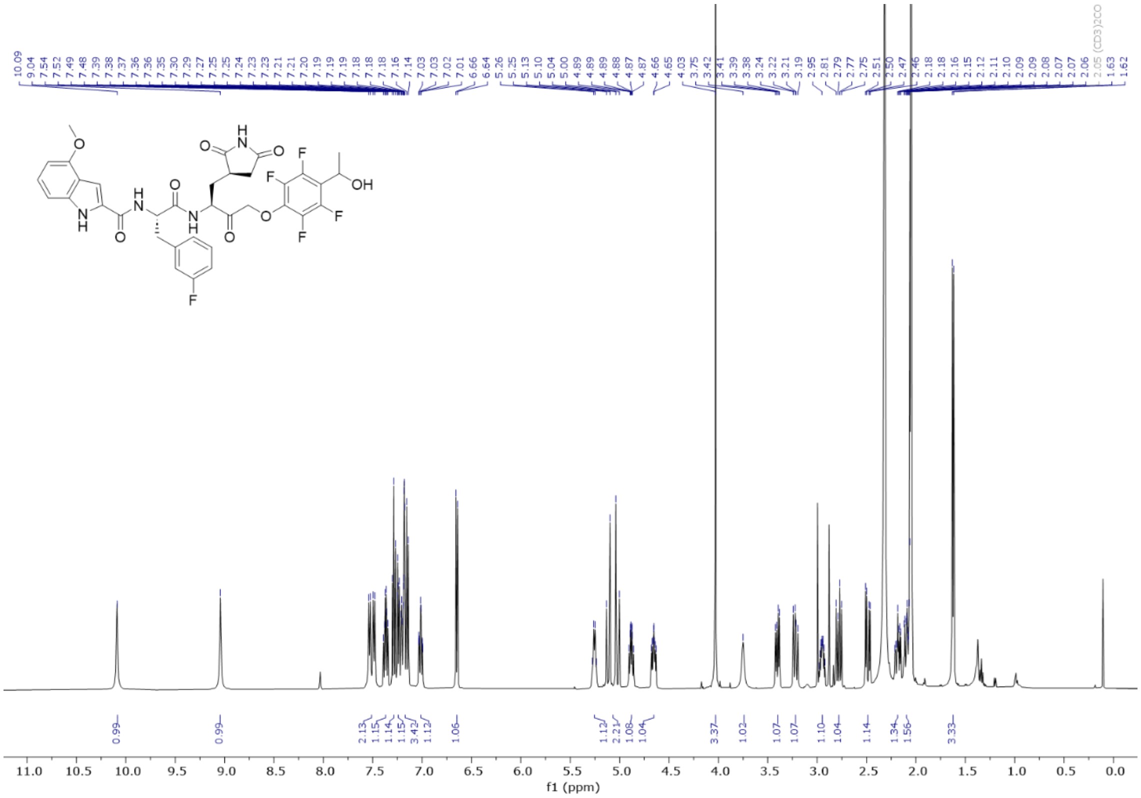

Figure S28. ^{1}H-NMR spectrum of **9**.

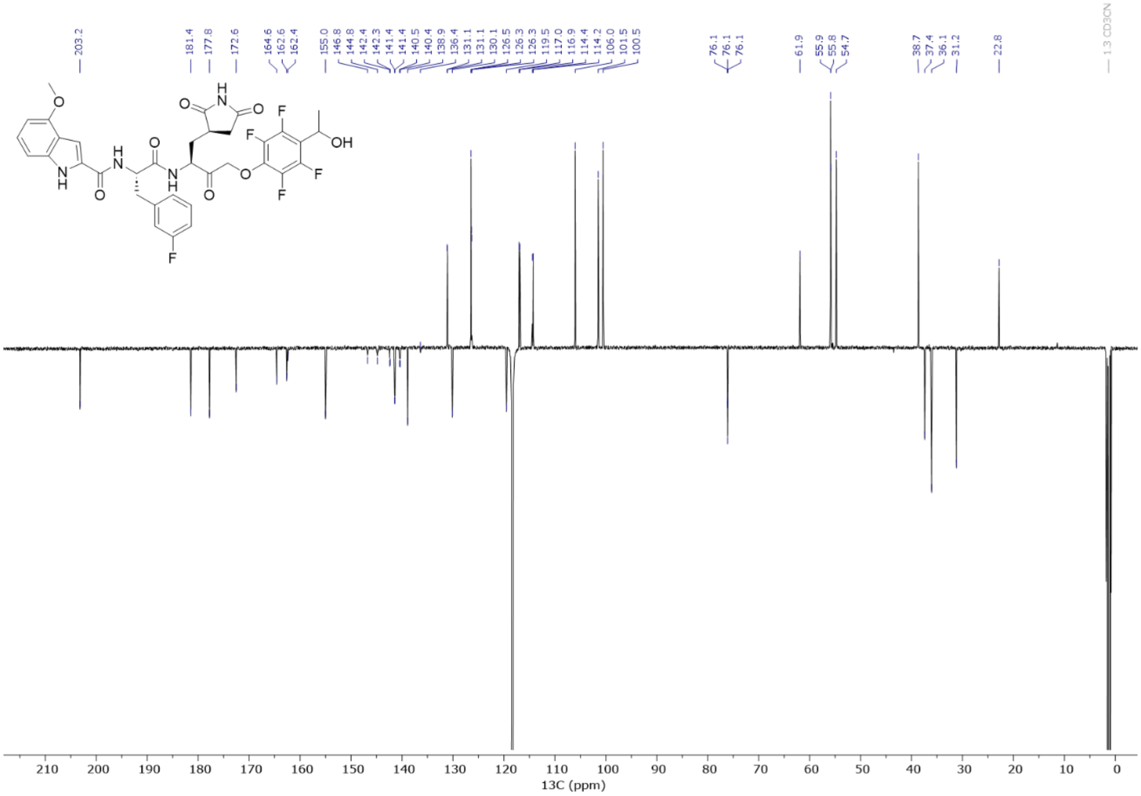

Figure S29. ^{13}C-NMR spectrum of **9**.

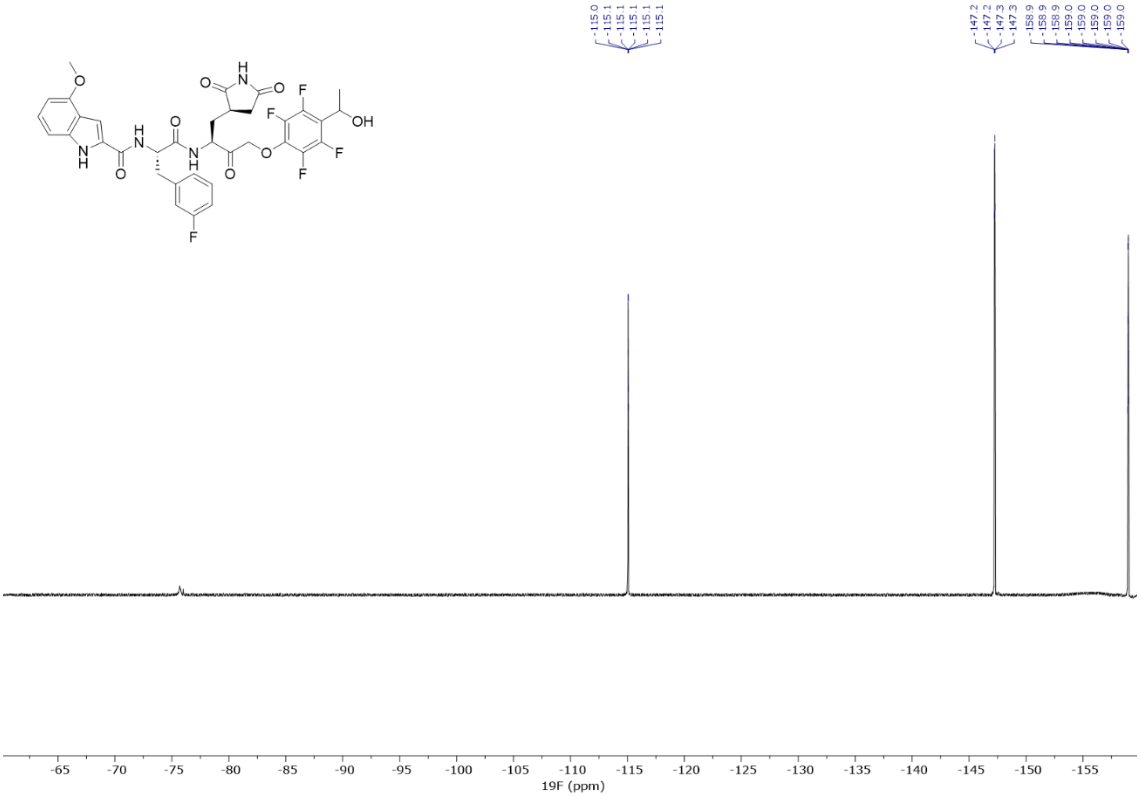

Figure S30. ^{19}F-NMR spectrum of **9**.

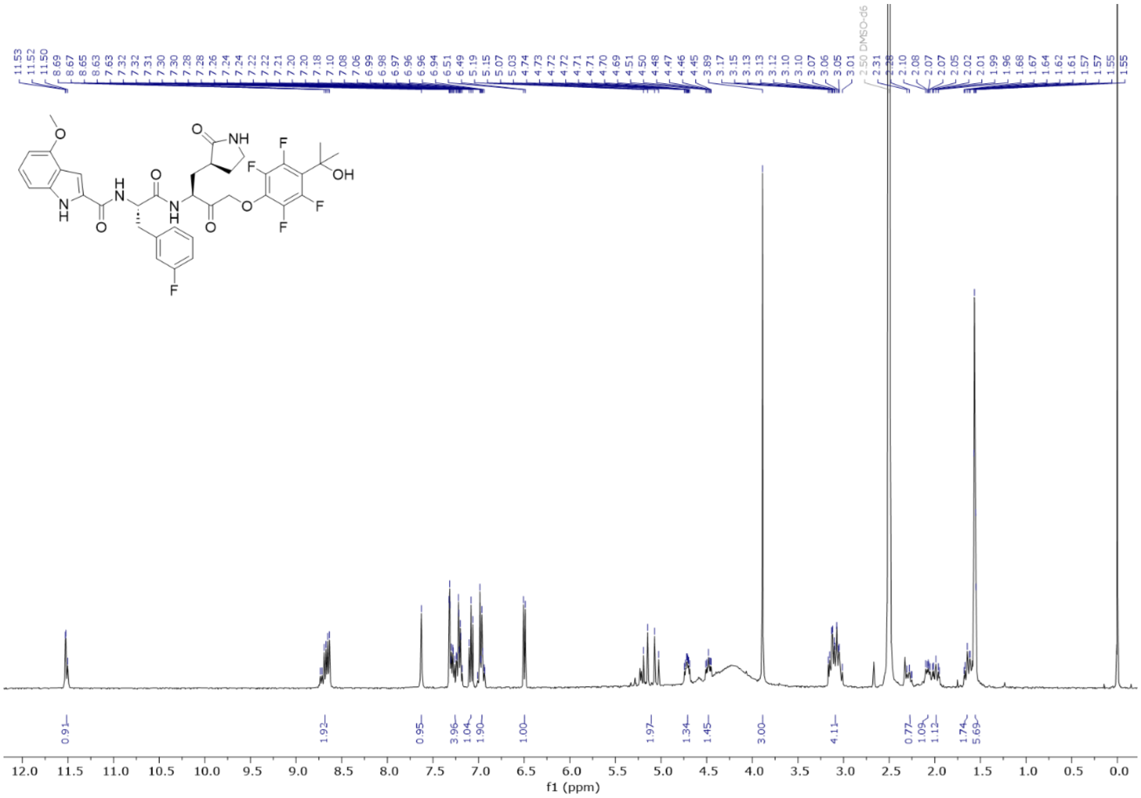

Figure S31. ^{1}H-NMR spectrum of **10**.

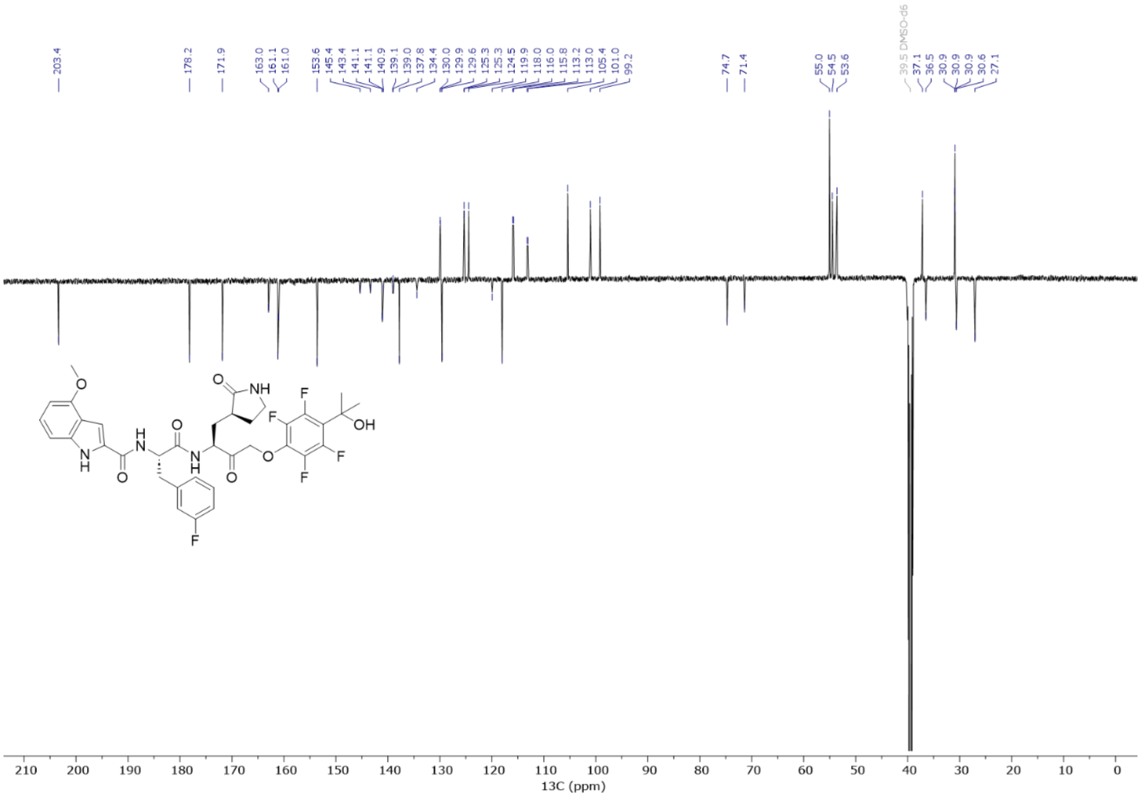

Figure S32. ^{13}C-NMR spectrum of **10**.

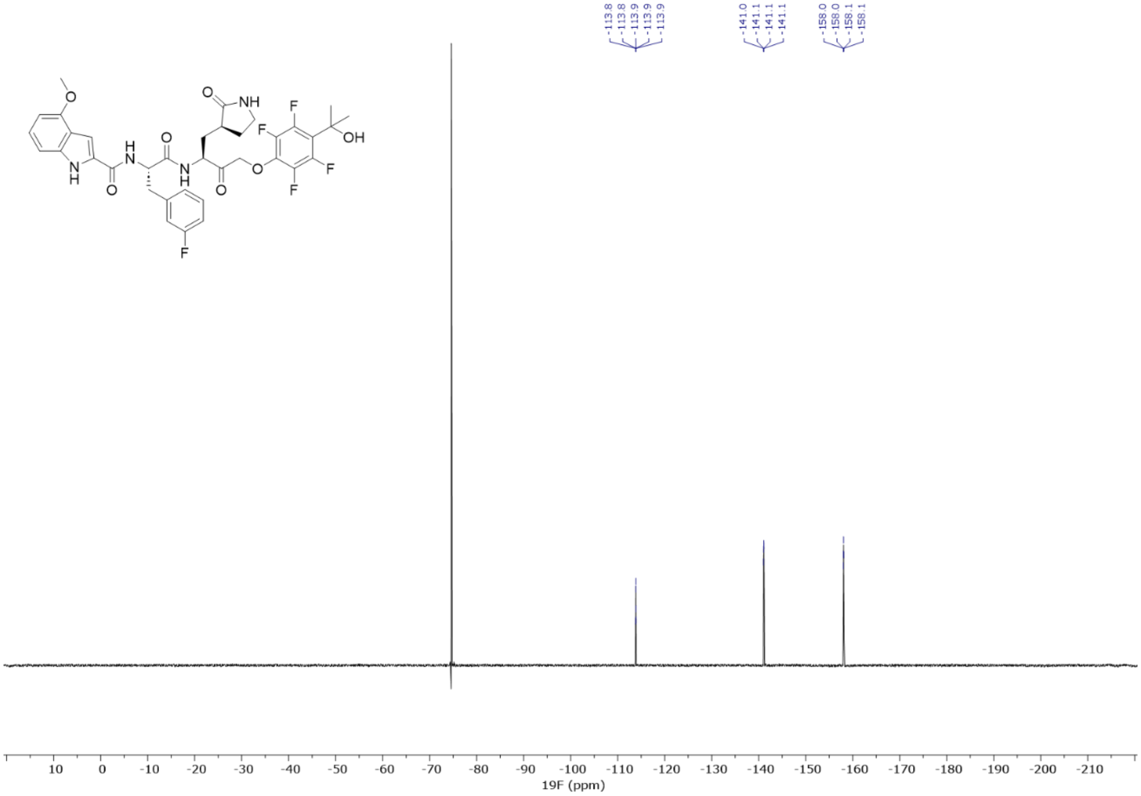

Figure S33. ^{19}F-NMR spectrum of **10**.

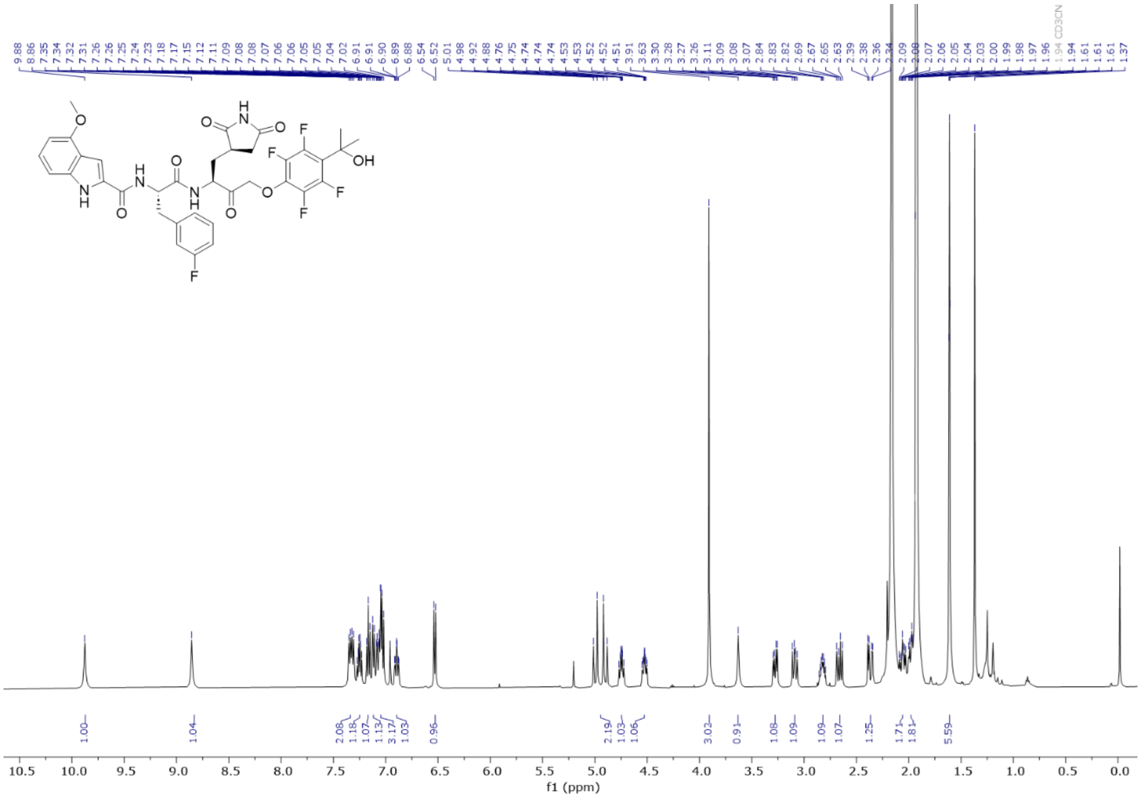

Figure S34. ^{1}H-NMR spectrum of **11**.

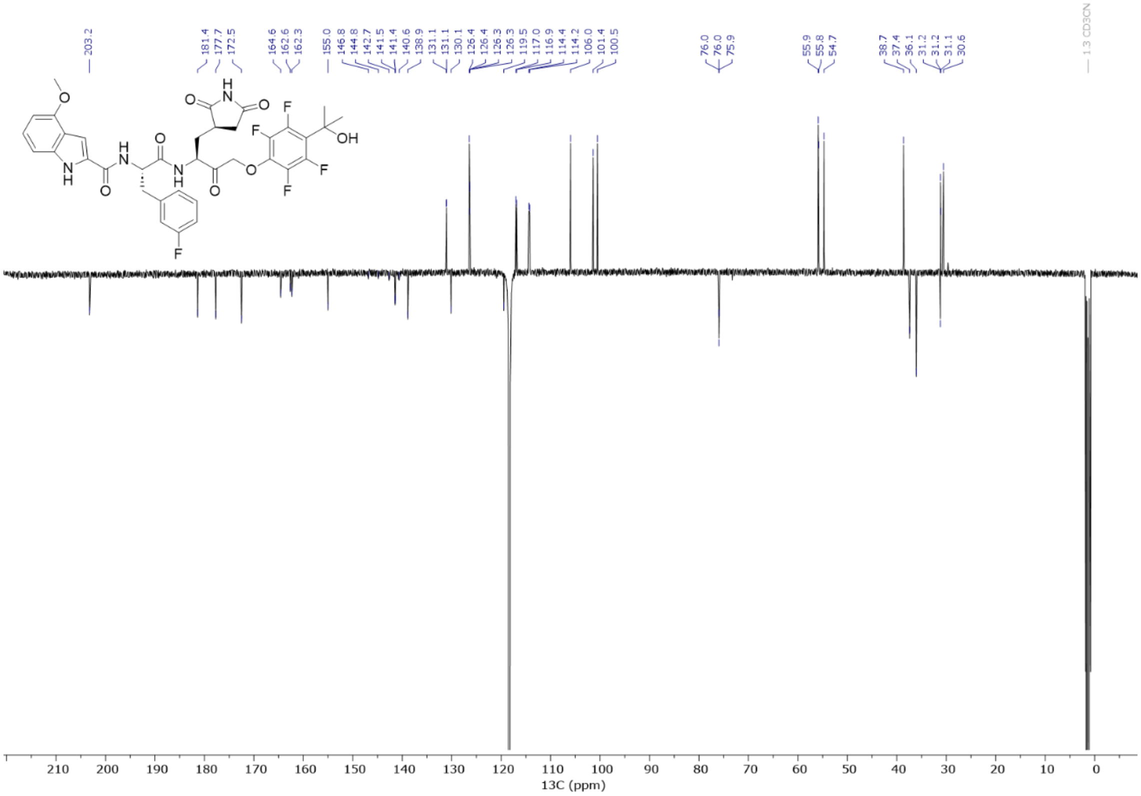

Figure S35. ^{13}C-NMR spectrum of **11**.

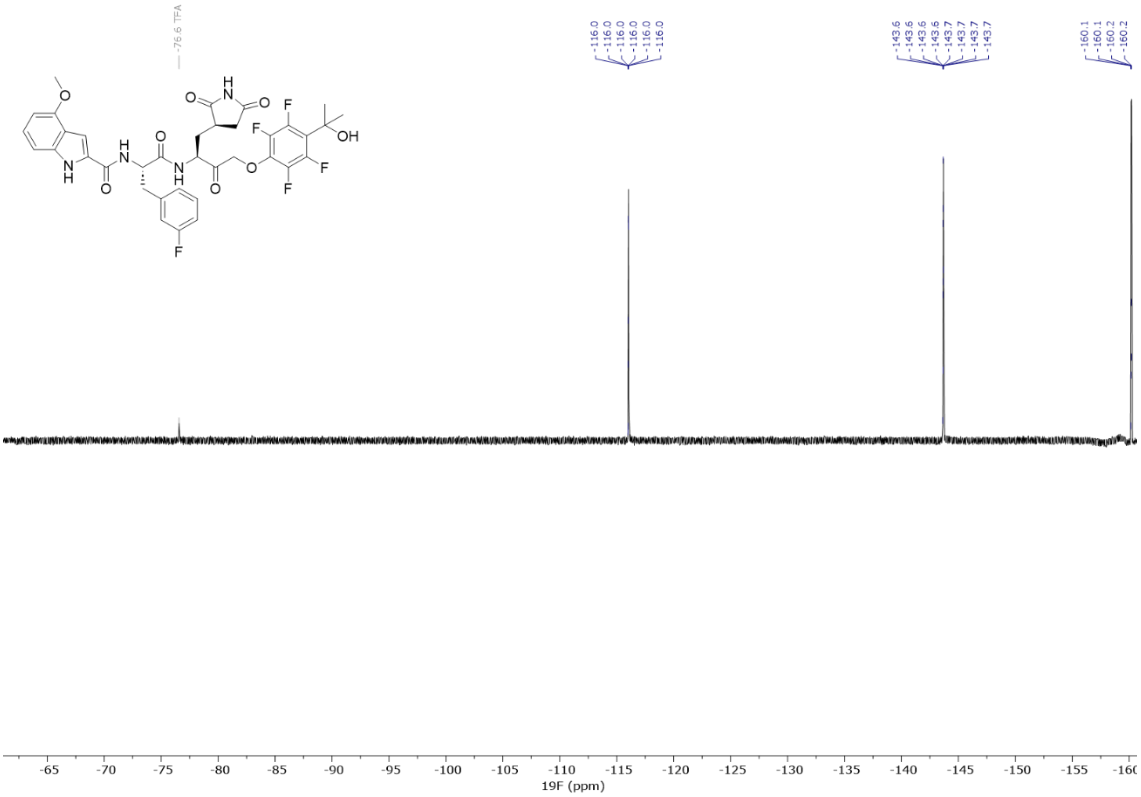

Figure S36. ^{19}F-NMR spectrum of **11**.

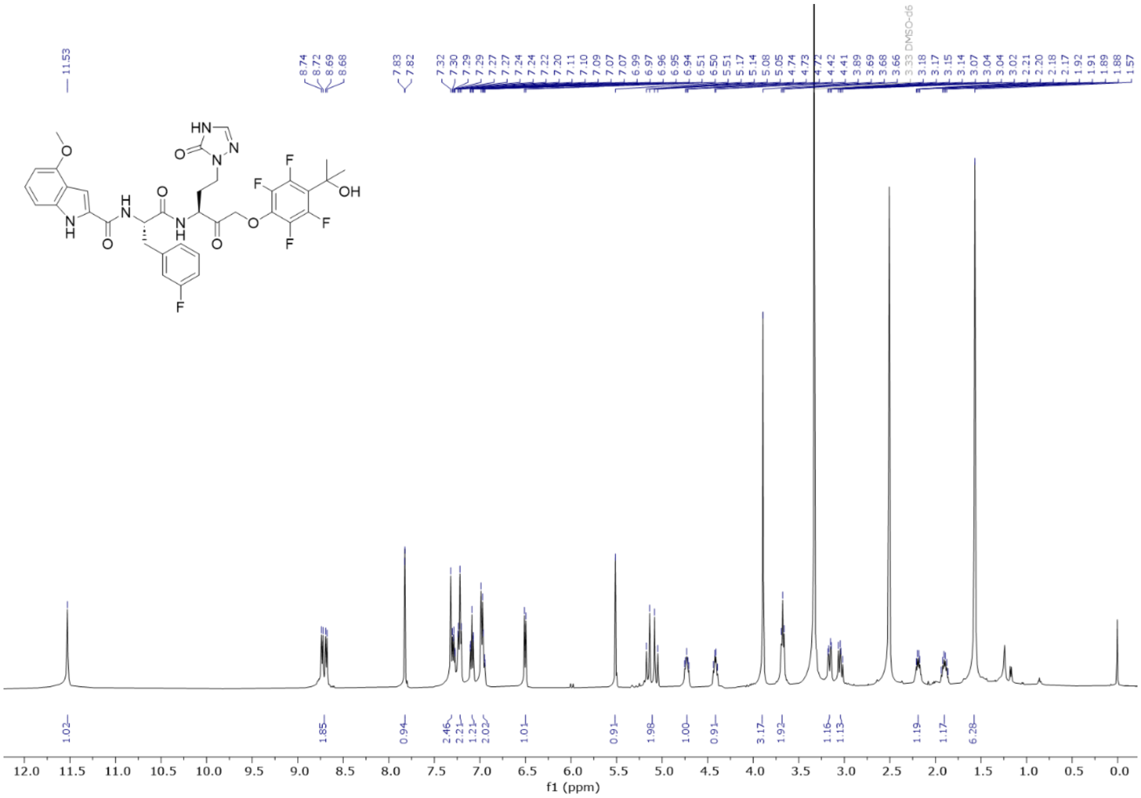

Figure S37. ^{1}H-NMR spectrum of **12**.

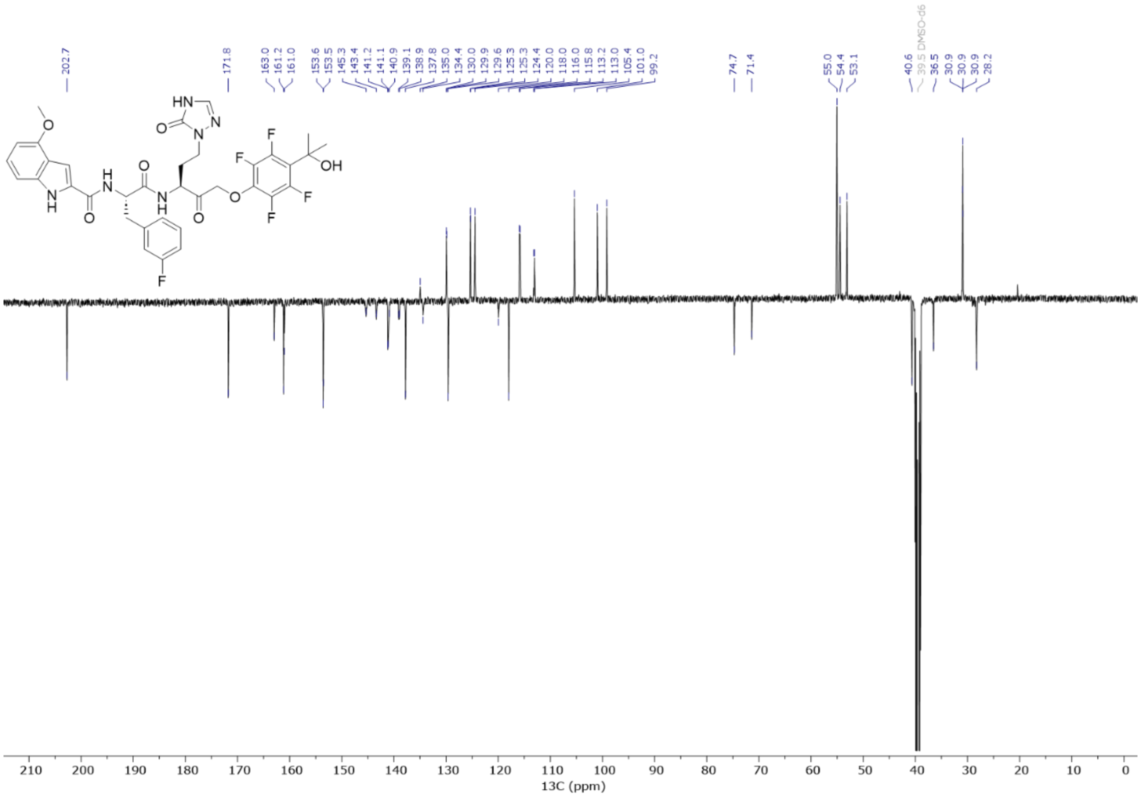

Figure S38. ^{13}C-NMR spectrum of **12**.

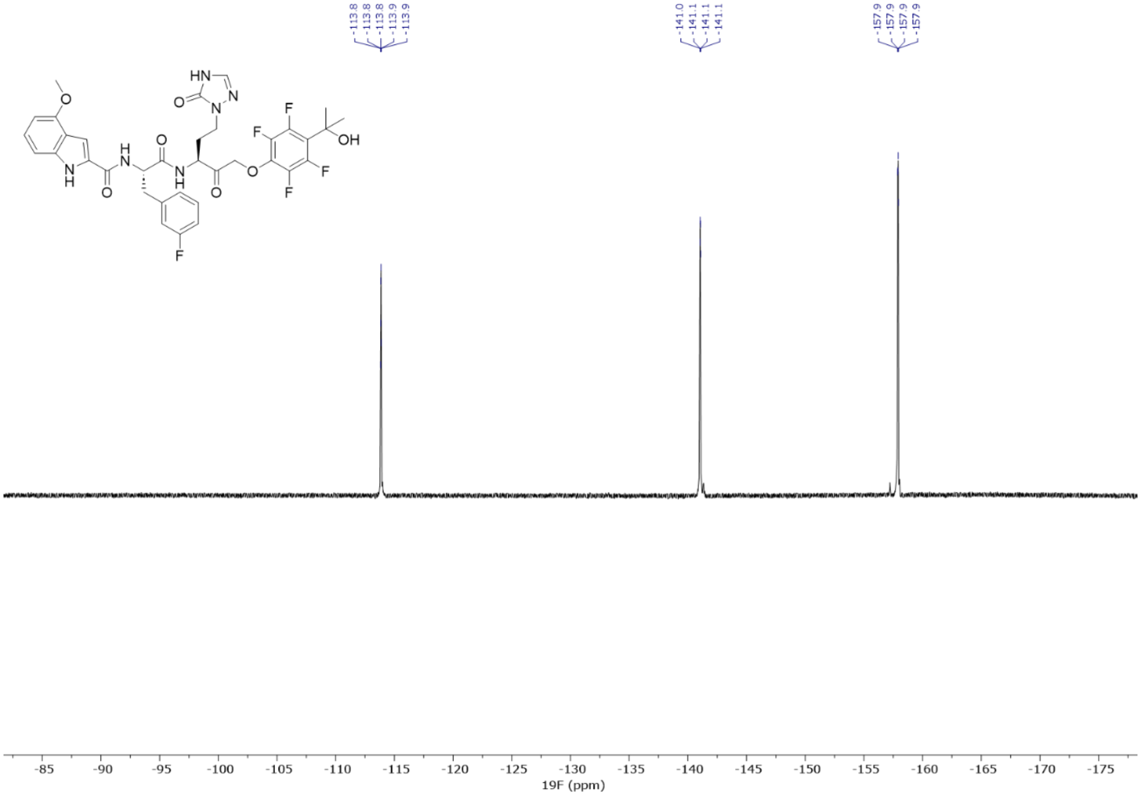

Figure S39. ^{19}F-NMR spectrum of **12**.

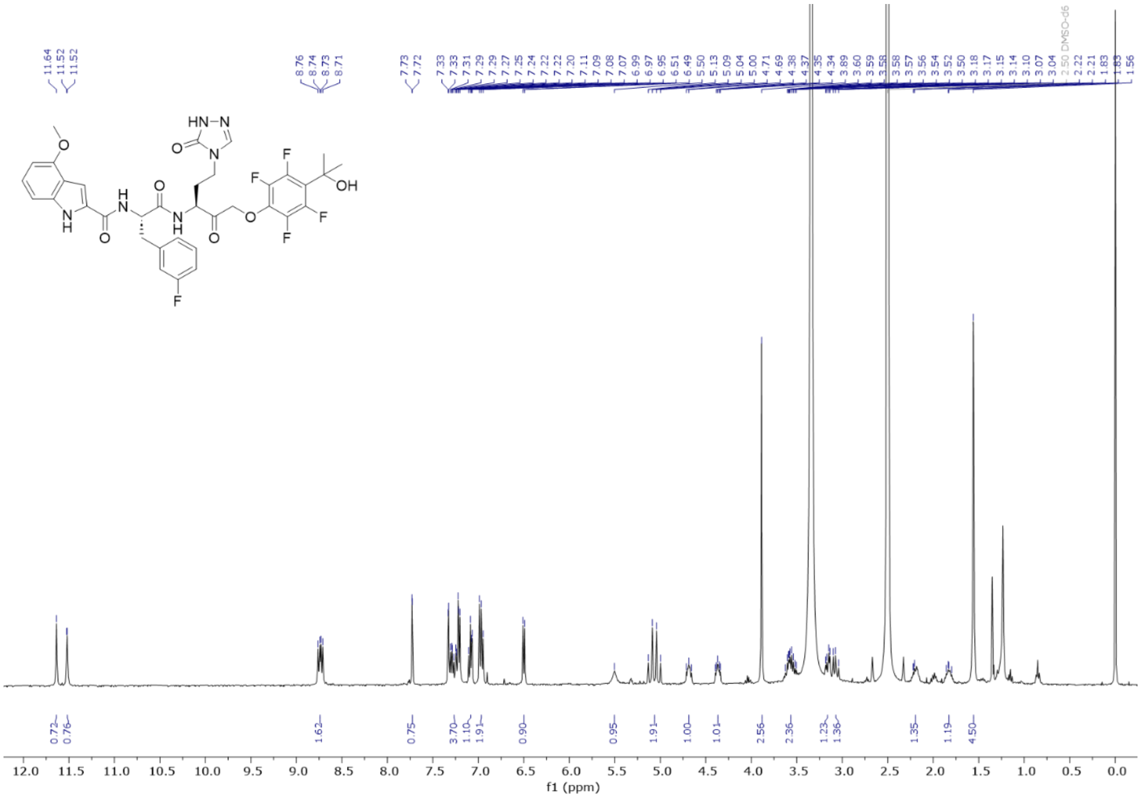

Figure S40. ^{1}H-NMR spectrum of **13**.

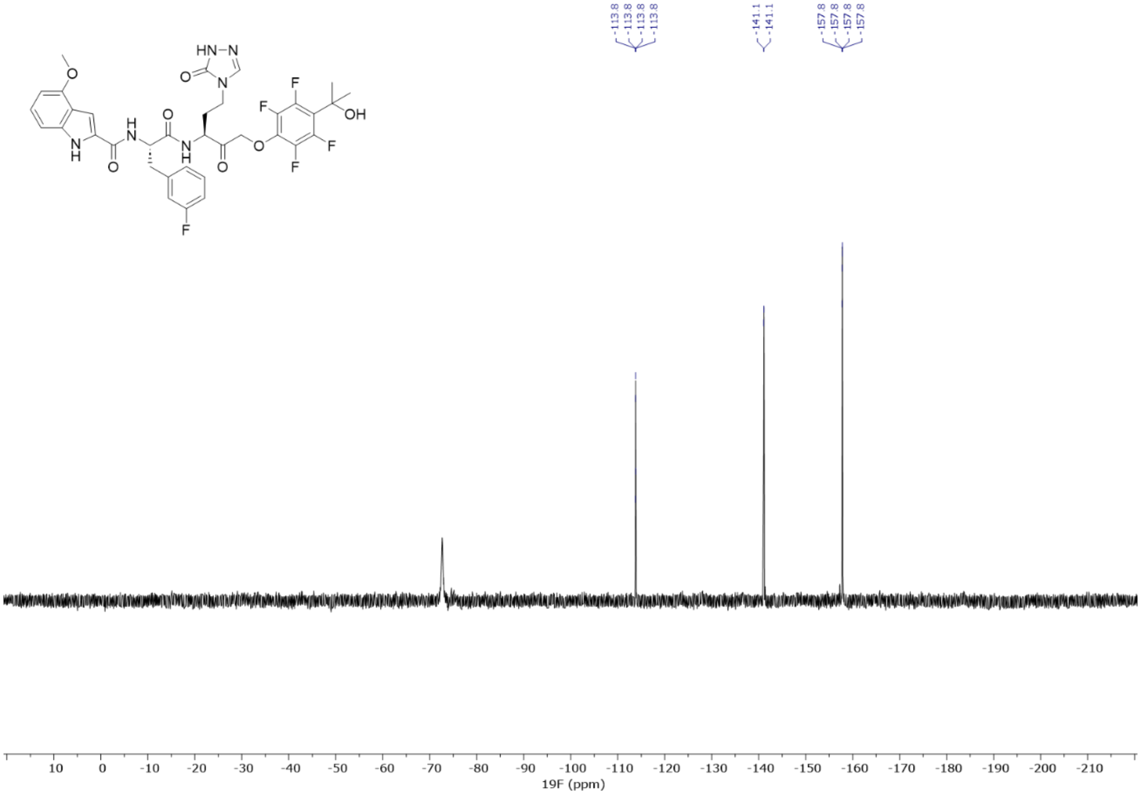

Figure S41. ^{19}F-NMR spectrum of **13**.

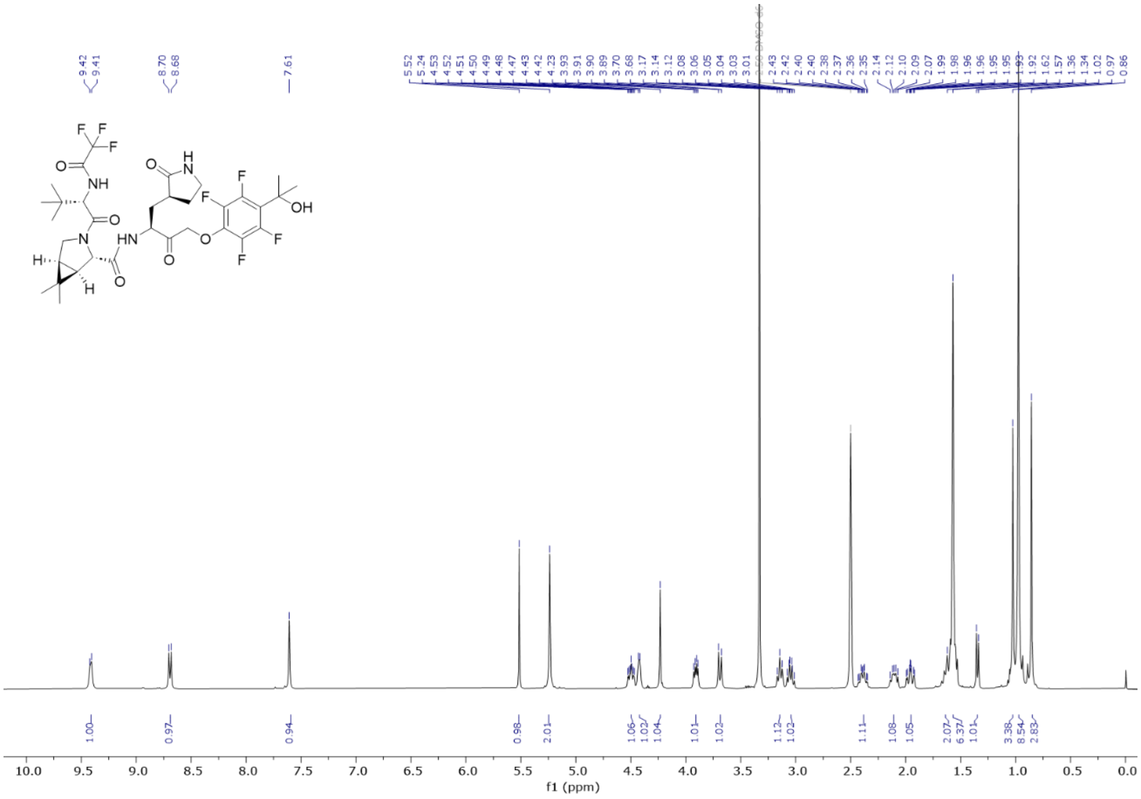

Figure S42. ^{1}H-NMR spectrum of **14**.

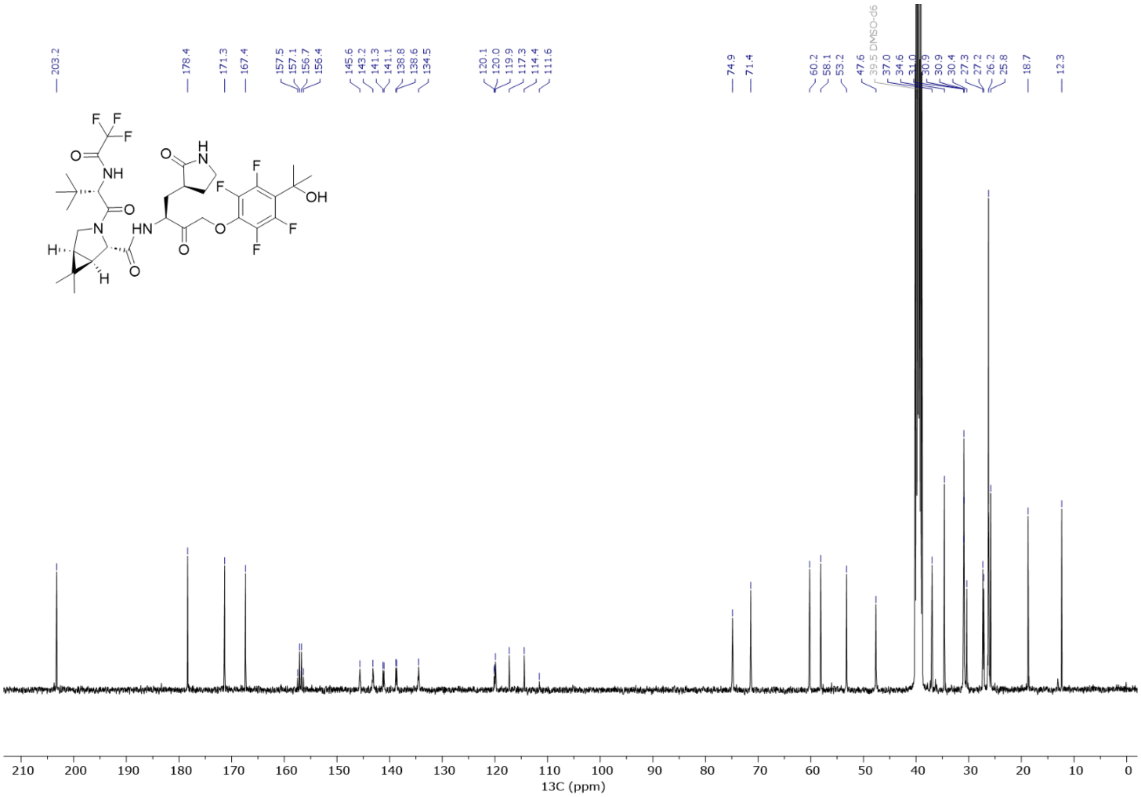

Figure S43. ^{13}C-NMR spectrum of **14**.

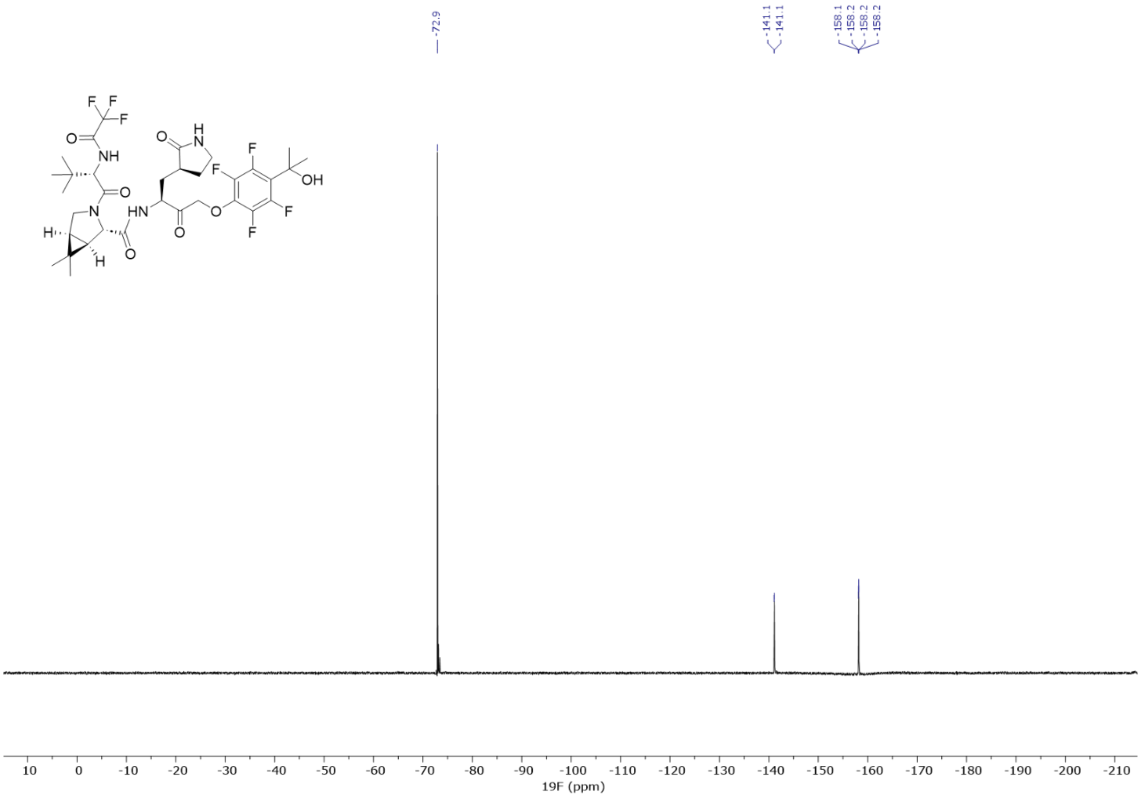

Figure S44. ^{19}F-NMR spectrum of **14**.

1.4 HPLC spectra

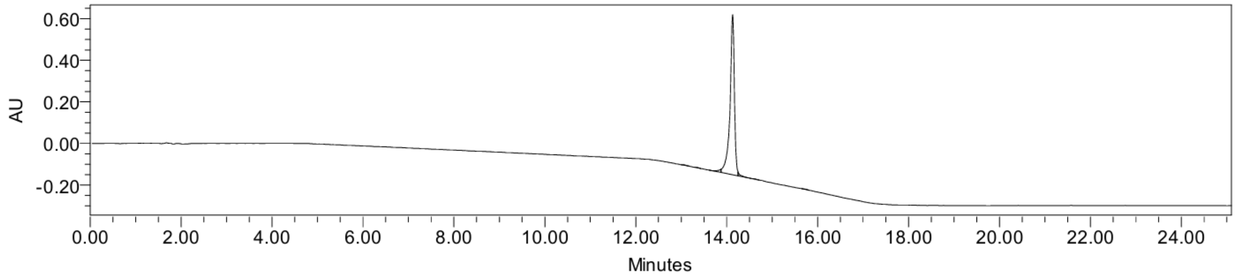

Figure S45. Analytical HPLC spectrum of **1** (97.1% pure, rt = 14.13 min).

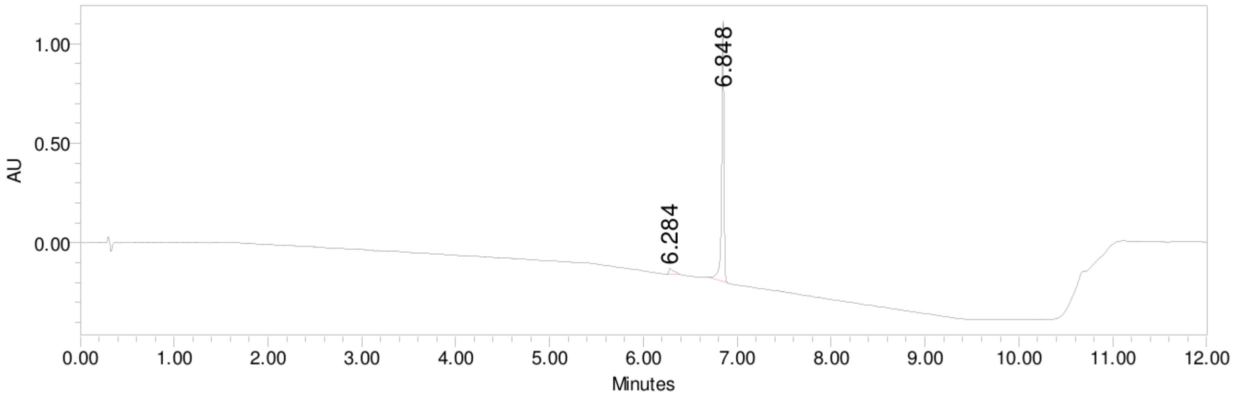

Figure S46. Analytical HPLC spectrum of **2** (95.3% pure, rt = 6.85 min).

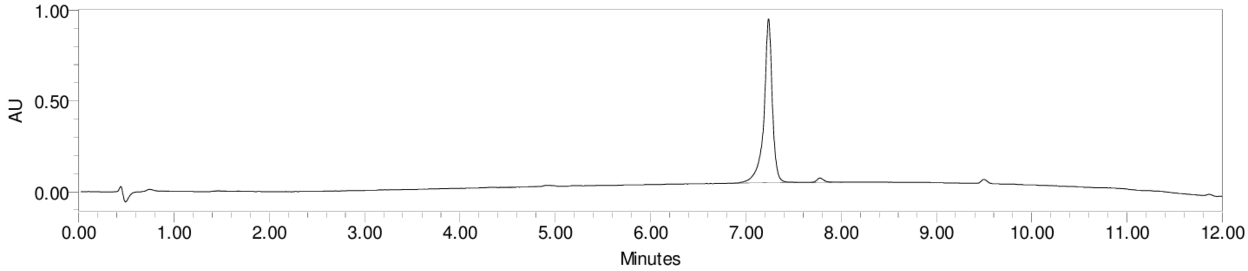

Figure S47. Analytical HPLC spectrum of **3** (97.3% pure, rt = 7.24 min).

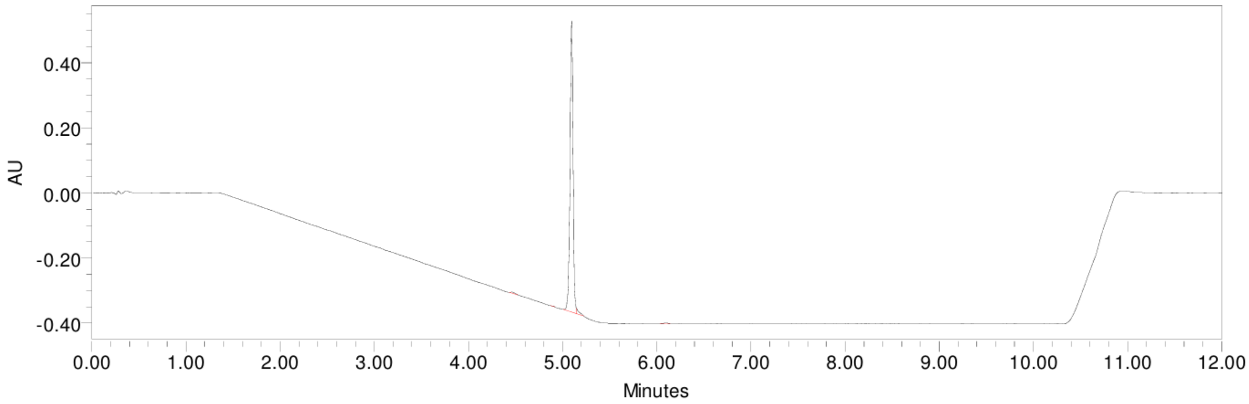

Figure S48. Analytical HPLC spectrum of **4** (97.7% pure, rt = 5.15 min).

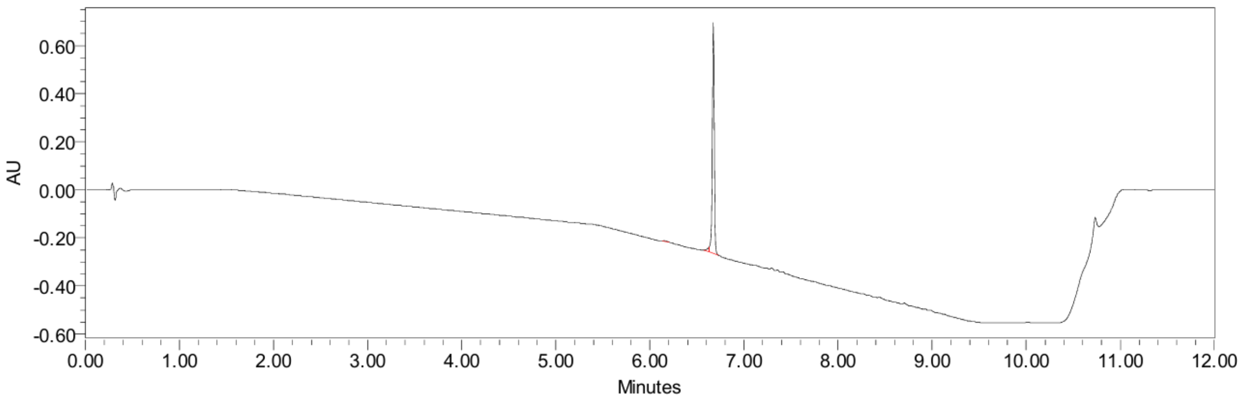

Figure S49. Analytical HPLC spectrum of **5** (97.4% pure, rt = 6.67 min).

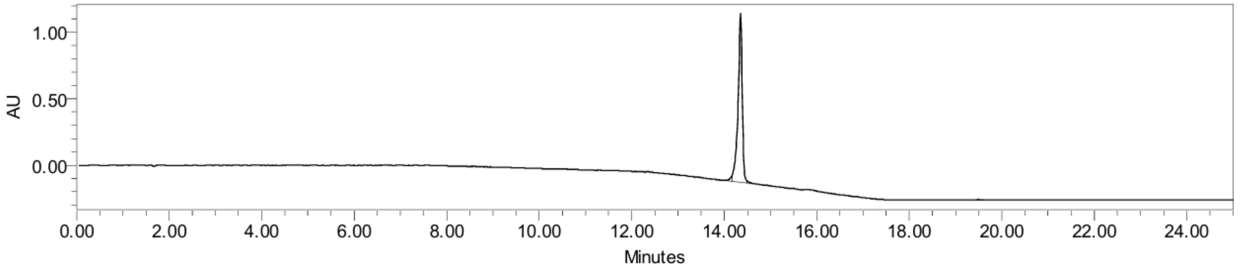

Figure S50. Analytical HPLC spectrum of **6** (97.9% pure, rt = 14.4 min).

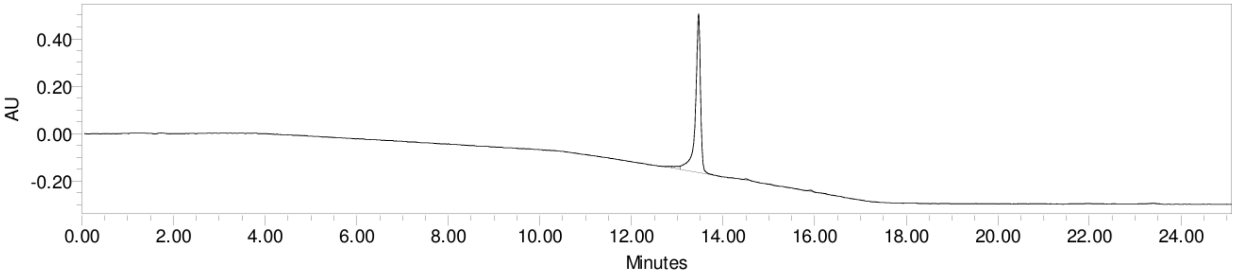

Figure S51. Analytical HPLC spectrum of **7** (96.1% pure, rt = 13.5 min).

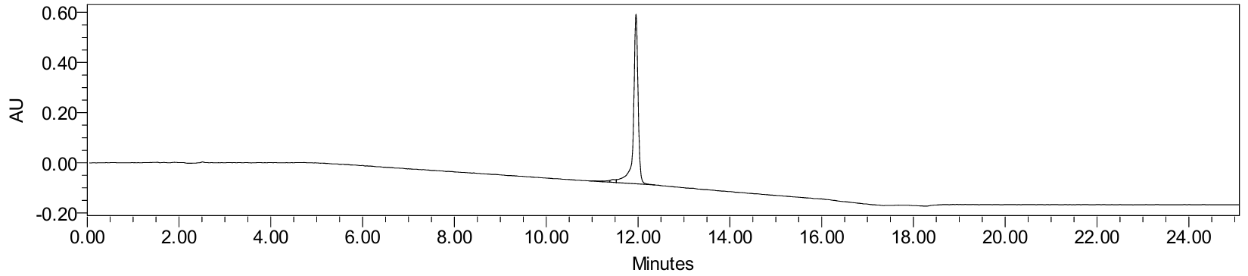

Figure S52. Analytical HPLC spectrum of **8** (96.8% pure, rt = 12.0 min).

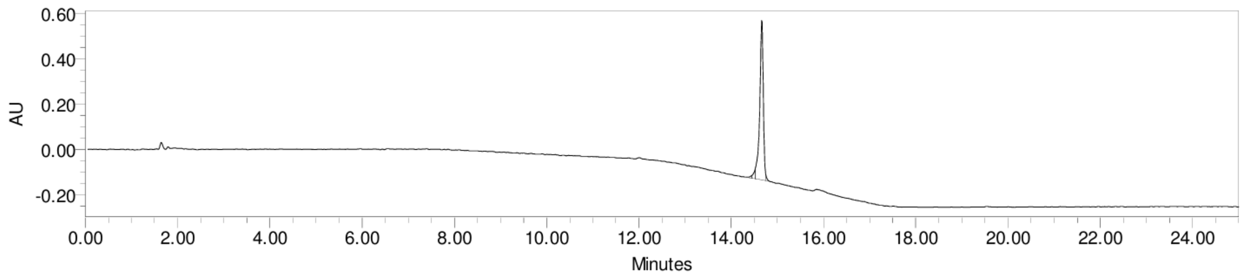

Figure S53. Analytical HPLC spectrum of **9** (95.7% pure, rt = 14.7 min).

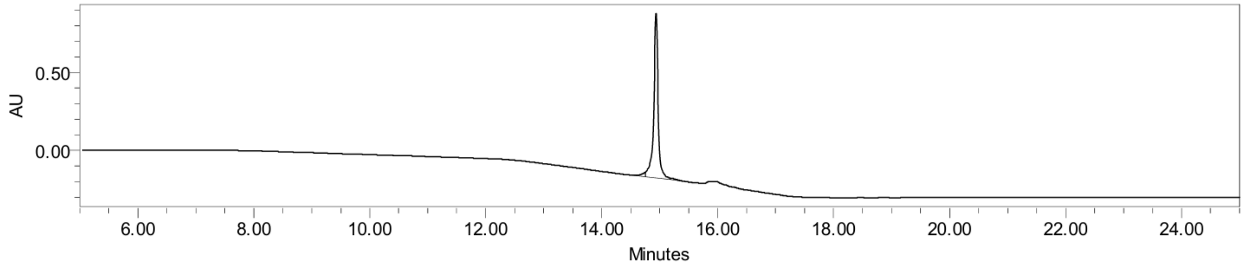

Figure S54. Analytical HPLC spectrum of **10** (96.7% pure, rt = 14.9 min).

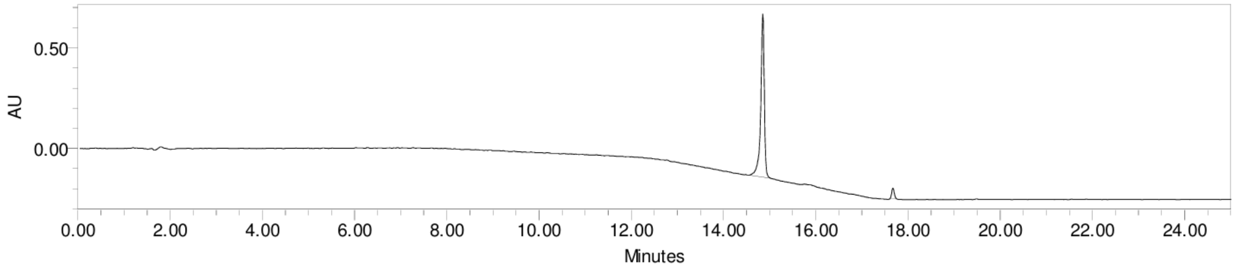

Figure S55. Analytical HPLC spectrum of **11** (95.1% pure, rt = 14.9 min).

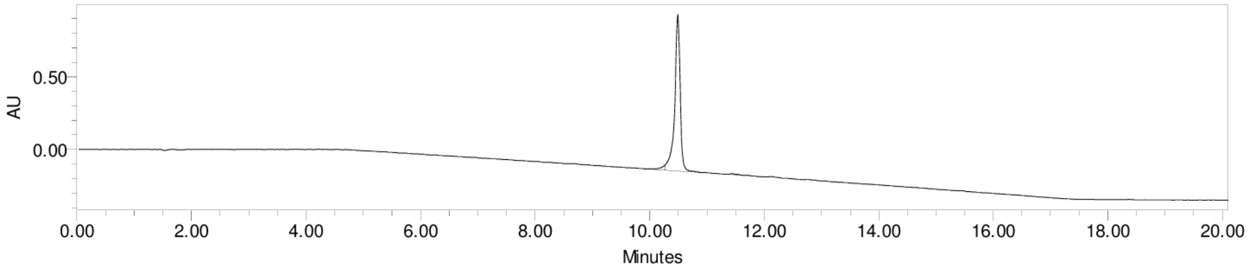

Figure S56. Analytical HPLC spectrum of **12** (96.3% pure, rt = 10.5 min).

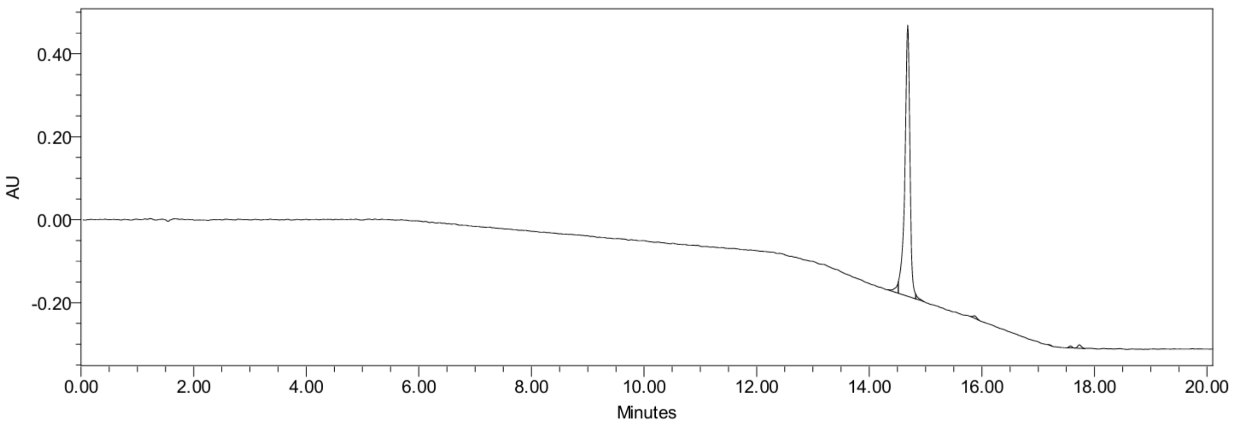

Figure S57. Analytical HPLC spectrum of **13** (95.3% pure, rt = 14.7 min).

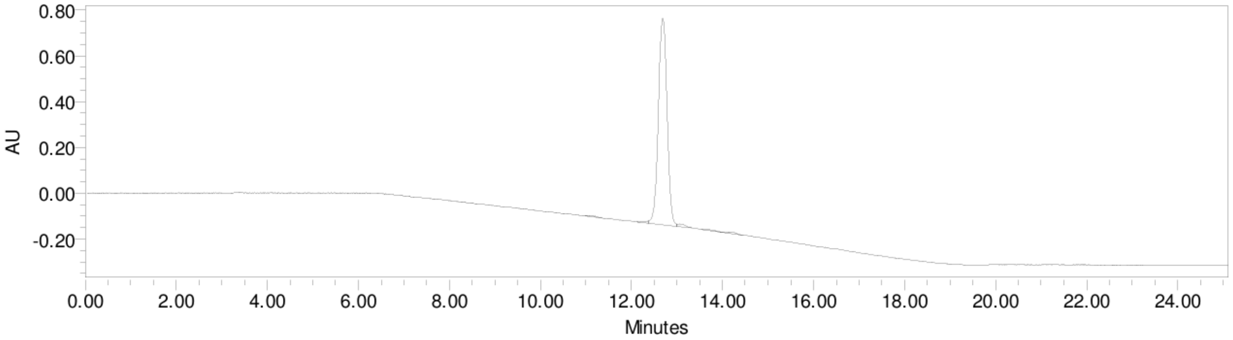

Figure S58. Analytical HPLC spectrum of **14** (96.5% pure, rt = 12.7 min).

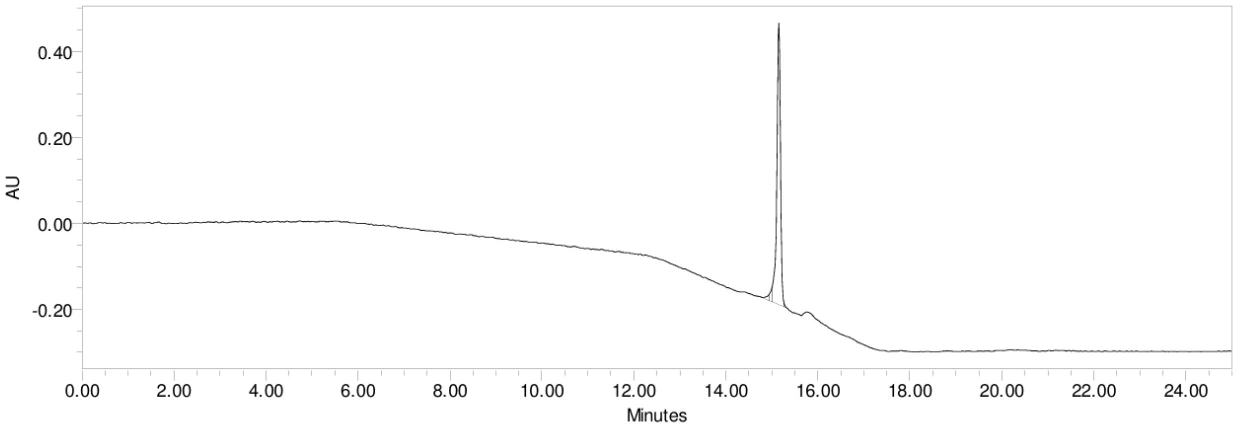

Figure S59. Analytical HPLC spectrum of **15** (95.6% pure, rt = 15.2 min).

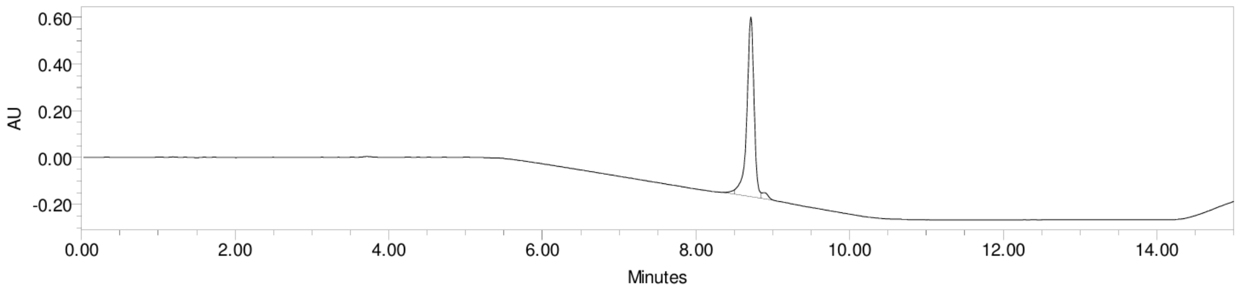

Figure S60. Analytical HPLC spectrum of **16** (96.1% pure, rt = 8.72 min).

1.5 References

1. Salgado-Benvindo, C.; Leijs, A.A.; Thaler, M.; Tas, A.; Arbiser, J.L.; Snijder, E.J.; van Hemert, M.J. Honokiol Inhibits SARS-CoV-2 Replication in Cell Culture at a Post-Entry Step. *Microbiol Spectr* **2023**, *11* (3), e0327322.
2. Thaler, M.; Salgado-Benvindo, C.; Leijs, A.; Tas, A.; Ninaber, D.K.; Arbiser, J.L.; Snijder, E.J.; van Hemert, M.J. R-Propranolol Has Broad-Spectrum Anti-Coronavirus Activity and Suppresses Factors Involved in Pathogenic Angiogenesis. *Int J Mol Sci* **2023**, *24* (5).
3. van der Linden, L.; Ulferts, R.; Nabuurs, S.B.; Kusov, Y.; Liu, H.; George, S.; Lacroix, C.; Goris, N.; Lefebvre, D.; Lanke, K.H.; De Clercq, K.; Hilgenfeld, R.; Neyts, J.; van Kuppeveld, F.J. Application of a cell-based protease assay for testing inhibitors of picornavirus 3C proteases. *Antiviral Res* **2014**, *103*, 17-24.
4. Wang, C.; Li, W.; Drabek, D.; Okba, N.M.A.; van Haperen, R.; Osterhaus, A.; van Kuppeveld, F.J.M.; Haagmans, B.L.; Grosveld, F.; Bosch, B.J. A human monoclonal antibody blocking SARS-CoV-2 infection. *Nat Commun* **2020**, *11* (1), 2251.
5. Nitsche, C.; Schreier, V.N.; Behnam, M.A.M.; Kumar, A.; Bartenschlager, R.; Klein, C.D. Thiazolidinone-Peptide Hybrids as Dengue Virus Protease Inhibitors with Antiviral Activity in Cell Culture. *Journal of Medicinal Chemistry* **2013**, *56* (21), 8389-8403.
6. Kühl, N.; Leuthold, M.M.; Behnam, M.A.M.; Klein, C.D. Beyond Basicity: Discovery of Nonbasic DENV-2 Protease Inhibitors with Potent Activity in Cell Culture. *Journal of Medicinal Chemistry* **2021**, *64* (8), 4567-4587.
7. Dai, W.; Zhang, B.; Jiang, X.M.; Su, H.; Li, J.; Zhao, Y.; Xie, X.; Jin, Z.; Peng, J.; Liu, F.; Li, C.; Li, Y.; Bai, F.; Wang, H.; Cheng, X.; Cen, X.; Hu, S.; Yang, X.; Wang, J.; Liu, X.; Xiao, G.; Jiang, H.; Rao, Z.; Zhang, L.K.; Xu, Y.; Yang, H.; Liu, H. Structure-based design of antiviral drug candidates targeting the SARS-CoV-2 main protease. *Science* **2020**, *368* (6497), 1331-1335.
8. ULC, C.C.G., Molecular Operating Environment (MOE), in *2022.02*. 2022, Chemical Computing Group ULC: 910-1010 Sherbrooke St. W., Montreal, QC H3A 2R7.
9. Maier, J.A.; Martinez, C.; Kasavajhala, K.; Wickstrom, L.; Hauser, K.E.; Simmerling, C. ff14SB: Improving the Accuracy of Protein Side Chain and Backbone Parameters from ff99SB. *Journal of Chemical Theory and Computation* **2015**, *11* (8), 3696-3713.
10. Gerber, P.R.; Muller, K. Mab, a Generally Applicable Molecular-Force Field for Structure Modeling in Medicinal Chemistry. *Journal of Computer-Aided Molecular Design* **1995**, *9* (3), 251-268.
11. Cui, H.; Divakaran, A.; Hoell, Z.J.; Ellingson, M.O.; Scholtz, C.R.; Zahid, H.; Johnson, J.A.; Griffith, E.C.; Gee, C.T.; Lee, A.L.; Khanal, S.; Shi, K.; Aihara, H.; Shah, V.H.; Lee, R.E.; Harki, D.A.; Pomerantz, W.C.K. A Structure-based Design Approach for Generating High Affinity BRD4 D1-Selective Chemical Probes. *J Med Chem* **2022**, *65* (3), 2342-2360.
12. Falke, S.; Lieske, J.; Herrmann, A.; Loboda, J.; Karnicar, K.; Gunther, S.; Reinke, P.Y.A.; Ewert, W.; Usenik, A.; Lindic, N.; Sekirnik, A.; Dretnik, K.; Tsuge, H.; Turk, V.; Chapman, H.N.; Hinrichs, W.; Ebert, G.; Turk, D.; Meents, A. Structural Elucidation and Antiviral Activity of Covalent Cathepsin L Inhibitors. *J Med Chem* **2024**, *67* (9), 7048-7067.
13. Wei, B.; Gunzner-Toste, J.; Yao, H.; Wang, T.; Wang, J.; Xu, Z.; Chen, J.; Wai, J.; Nonomiya, J.; Tsai, S.P.; Chuh, J.; Kozak, K.R.; Liu, Y.; Yu, S.F.; Lau, J.; Li, G.; Phillips, G.D.; Leipold, D.; Kamath, A.; Su, D.; Xu, K.; Eigenbrot, C.; Steinbacher, S.; Ohri, R.; Raab, H.; Staben, L.R.; Zhao, G.; Flygare, J.A.; Pillow, T.H.; Verma, V.; Masterson, L.A.; Howard, P.W.; Safina, B. Discovery of Peptidomimetic Antibody-Drug Conjugate Linkers with Enhanced Protease Specificity. *J Med Chem* **2018**, *61* (3), 989-1000.
14. Wang, H.; Yang, Q.; Liu, X.; Xu, Z.; Shao, M.; Li, D.; Duan, Y.; Tang, J.; Yu, X.; Zhang, Y.; Hao, A.; Wang, Y.; Chen, J.; Zhu, C.; Guddat, L.; Chen, H.; Zhang, L.; Chen, X.; Jiang, B.; Sun, L.; Rao, Z.; Yang, H. Structure-based discovery of dual pathway inhibitors for SARS-CoV-2 entry. *Nat Commun* **2023**, *14* (1), 7574.
